# Supplementary material for: Analysis of systemic effects of dioxin on human health through template-and-anchor modeling
Source: PLoS Comput Biol. 2025 Mar 27;21(3):e1012840. doi: 10.1371/journal.pcbi.1012840 (PMC12005559; doi:10.1371/journal.pcbi.1012840)
Supplement: S1 Text — All relative sensitivities are below 1 in magnitude. Fig B. Results of a local sensitivity analysis for steroidogenesis pathway anchor model. All parameters have a sensitivity coefficient below 1 in magnitude. Fig C. Results of a sensitivity analysis of the template model. All parameters values exhibit changes in the steady-state values of less than 10% in magnitude, corresponding to absolute values of relative sensitivities <1. Fig D. Generic PBPK model as a multiscale structure. The primary scale of granularity reflects organs and tissues, but the drug concentrations in these organs are subject to molecular and cellular events, such as sequestration and degradation in the liver. In this illustration, the drug or toxicant enters the body per inhalation. (DOCX) [file pcbi.1012840.s001.docx]

**Supplements**

Analysis of Systemic Effects of Dioxin on Human Health

through Template-and-Anchor Modeling

**Carla M. Kumbale, Qiang Zhang, and Eberhard O. Voit**

**1. Design of a Template-and-Anchor (T&A) Model**

The primary focus of the paper is the analysis of organism-wide effects of dioxin exposure on cholesterol handling. We use for this analysis a new instantiation of the T&A modeling paradigm [1], as discussed in the Text and elsewhere [2].

The implementation of the T&A concept mandates the choice of a mathematical representation, for which we selected Mass-Action Kinetics (MAK) and Biochemical Systems Theory (BST) [3-7] because their tenets constitute a very effective and essentially unbiased compromise between biomedical realism and mathematical tractability. BST, along with its prominent special case MAK, always represents systems as sets of nonlinear ordinary differential equations (ODEs), in which each process *P_i_* is formulated as a product of power-law functions that contain all variables *X_j_* directly contributing to this process, each raised to a power $a_{ikj}$, called a *kinetic order*. In addition, a non-negative rate constant $v_{ik}$ determines the turnover rate of the process. Thus, each process representation has the format

$P_{i}=v_{ik}\cdot X_{1}^{a_{ik1}}\cdot X_{2}^{a_{ik2}}\cdot\ldots\cdot X_{n}^{a_{ikn}}$.

If a kinetic order is positive, it signifies a positive effect on the process, while a negative value signifies an inhibitory or diminishing effect. Typical kinetic orders vary between -1 and +2, while the rate constants may take any non-negative values. A rich literature has documented the theoretical underpinnings of BST as well as numerous applications. For a comprehensive review, see [6].

Representative models discussed in the Text are available in Matlab and PLAS implementation at GitHub.com/LBSA-VoitLab/TCDD_Chol. PLAS is proposed because it is a free ODE analyzing software that is very easy to learn even for readers with only a modest computational background. It can be found at:

<https://arquivo.pt/wayback/20230321162314/http://enzymology.fc.ul.pt/software/plas/>.

2. Model Equations

*2.1. Cholesterol Biosynthesis*

The equations of this model were discussed in [2]. They can be found at GitHub.com/LBSA-VoitLab/TCDD_Chol.

*2.2. Lipoprotein Transport Anchor Model*

**Hepatic Cholesterol:**

$$\dot{HC}= Diet+ c_{1}\cdot HC^{k_{1}}\cdot{DR}_{1}- c_{2}\cdot HC\cdot ACAT^{k_{1}}\cdot{DR}_{2}- c_{3}\cdot HC \cdot{DR}_{3} - c_{5}\cdot HC\cdot{DR}_{4} + c_{6}\cdot VLDL*{DR}_{5} + c_{7}\cdot IDL \cdot{DR}_{6}+ c_{8}\cdot LDL \cdot{DR}_{7}+ c_{9}\cdot HDL\cdot{DR}_{8}$$

$$+ c_{4}\cdot Cstor$$

**VLDL:**

$$\dot{VLDL}= c_{5}\cdot HC\cdot{DR}_{4}- c_{6}\cdot VLDL\cdot{DR}_{5} - c_{10}\cdot VLDL\cdot{LPL}^{k_{2}}\cdot{DR}_{9}+ c_{12}\cdot HDL$$

**IDL:**

$$\dot{IDL}= c_{10}\cdot VLDL\cdot{LPL}^{k_{2}}\cdot{DR}_{9}- c_{7}*IDL \cdot{DR}_{6} - c_{21}\cdot IDL\cdot{DR}_{10}$$

**LDL:**

$$\dot{LDL}= c_{11}\cdot HDL - c_{8}\cdot LDL \cdot{DR}_{7}+ c_{21}\cdot IDL\cdot{DR}_{10}- c_{22}\cdot LDL$$

**HDL:**

$$\dot{HDL}= c_{13}\cdot LCAT^{k_{3}}\cdot{DR}_{11}- c_{9}\cdot HDL\cdot{DR}_{8} - c_{11}\cdot HDL - c_{12}\cdot HDL$$

**Cholesterol Storage:**

$$\dot{Cstor}= c_{2}\cdot HC\cdot ACAT^{k_{1}}\cdot{DR}_{2}- c_{4}\cdot Cstor$$

Parameter Values of the Lipoprotein Transport Anchor Model

The quantities *DR_i_* are dioxin-dependent modulators discussed in the text. They have values of 1 at the baseline without dioxin exposure.

*2.3. Steroidogenesis Anchor Model*

**Pregnenolone (P5):**

$\dot{P5}=p_{k2}\left( p_{k2a}\cdot AF+p_{k2b}\cdot CL \right)\cdot Chol-p_{k3}\cdot P5-p_{k11}\cdot P5$

**Progesterone (P4):**

$\dot{P4}=p_{k3}\cdot P5-p_{k4}\cdot CL\cdot P4-p_{k5}\cdot P4$

**Androstenedione (A4):**

$\dot{A4}=p_{k5}\cdot P4-p_{k6}\cdot A4+p.k7\cdot T-p_{k12}\cdot A4$

**Testosterone (T):**

$\dot{T}=p_{k6}\cdot A4-p.k7\cdot T-p_{k8}\cdot T-p_{k13}\cdot T$

**Estradiol (E2):**

$\dot{E2}=p_{k8}\cdot T-p_{k}\cdot E2-p_{k22} \cdot\frac{LHp^{p.n22}}{J{22}^{p.n22}+LHp^{p.n22}}\cdot E2$

**Estradiol in Plasma (E2p):**

$\dot{E2}p=p_{E2p} \cdot p_{k9} \cdot E2-p_{k10} \cdot E2p$

**Progesterone in Plasma (P4p):**

$\dot{P4p}=p_{P4p}\cdot p_{k4}\cdot CL\cdot P4-p_{k14}\cdot P4p$

**LH in Pituitary (LH):**

$\dot{\mathrm{LH}}=p_{k15}-p_{k25}\cdot\frac{S^{p.n25}}{{p_{j25}}^{p.n25}+S^{p.n25}}\cdot LH-p_{k18}\cdot LH$

**LH in Plasma (LHp):**

$\dot{\mathrm{LHp}}=p_{k25}\cdot\frac{S^{p_{n25}}}{p.J{25}^{p_{n25}}+S^{p_{n25}}}\cdot LH+p_{k18}\cdot LH-p_{k17}\cdot LHp$

**Corpus Luteum (CL):**

$\dot{\mathrm{CL}}=p_{k29}\cdot\frac{S1^{p_{n29}}}{p.J{29}^{p_{n25}}+S1^{p_{n29}}}-p_{k20}\cdot CL-p_{k32}\cdot\frac{S2^{p_{n32}}}{p.J{32}^{p_{n32}}+S2^{p_{n32}}}\cdot CL$

**Growing Antral Follicles (AF):**

$\dot{\mathrm{AF}}=p_{k21}\cdot AF^{2}\left( 1-\frac{AF}{p_{AFmax}} \right)-p_{k29}\cdot\frac{S1^{p.n29}}{p.J{29}^{p.n29}+S1^{p.n29}}\cdot AF$

**Bistable Signal S Mediating a Positive Effect of E2p onto LH Release (S):**

$\dot{S}=p_{k160}+p_{k16}\cdot\frac{S^{p.n16}}{\left( \frac{p.J16}{p.k24+E2p} \right)^{p.n16}+S^{p.n16}}-p_{k23}\cdot S$

**Bistable Signal S1 Mediating the LH Surge-Triggered AF Collapse and CL Formation (S1):**

$\dot{S1}=p_{k190}+p_{k19}\cdot\frac{S1^{p.n19}}{\left( \frac{p.J19}{p.k26+p.k28\cdot LHp} \right)^{p.n19}+S1^{p.n19}}-p_{k27}\cdot S1$

**Intermediate Promoting CL Atresia (S2):**

$\dot{S2}=p_{k30}\cdot\frac{S1^{p.n30}}{p.J{30}^{p.n30}+S1^{p.n30}}-p_{k31}\cdot S2$

**FSH in Pituitary (FSH):**

$\dot{\mathrm{FSH}}=p_{k34}\cdot\frac{p.J{34}^{p.n34}}{p.J{34}^{p.n34}+P4p^{p.n38}}\cdot p_{k38}\cdot\frac{p.J{38}^{p.n38}}{p.J{38}^{p.n38}+E2p^{p.n38}}-p_{k37}\cdot\frac{S^{p.n37}}{p.J{37}^{p.n37}+S^{p.n37}}\cdot FSH-p_{k35}\cdot FS$

**FSH in Plasma (FSHp):**

$\frac{dFSHp}{dt}=p_{k37}\cdot\frac{S^{p.n37}}{p.J{37}^{p.n37}+S^{p.n37}}\cdot FSH+p_{k35}\cdot FSH-p_{k36}\cdot FSHp$

Parameter Values of the Steroidogenesis Anchor Model

(all parameters in the equations above are indicated with the prefix “p”)

**Population-Based Model:**

*Details of the Monte-Carlo simulation for parameter randomization*

**

3. Simulating Dioxin Exposure in PLAS

The effect of dioxin is coded either as a positive or negative modulator. Specifically, dioxin is coded as DEP or DEN, which corresponds to DR_i_ in the ODEs.

DR_i_ = 1 // no effect of TCDD

DR_i_  > 1 // “DEP,” positive effect of TCDD

DR_i_  < 1 // “DEN,” negative effect of TCDD

Specifically, DEP codes for a positive dioxin effect on some process and is implemented by an incremental increase in value, *i.e.*, 1.1, 1.2, 1.3… DEN codes for a negative dioxin effect on some process and is implemented by an incremental decrease in value, *i.e.*, 0.9, 0.8, 0.7… DEP and DEN maybe in effect simultaneously. As an example, the effects of dioxin affects on generic rate constants is modeled as:

*v*_11_ = rate constant at baseline $\cdot$ DEN

*v*_21_ = rate constant at baseline $\cdot$ DEP

For the mevalonate anchor model, we matched our simulation results to the cholesterol synthesis rate found in the literature [8] in order to determine dioxin dosage and the resulting concentration of hepatic cholesterol [2]. The synthetic rate was calculated as percent of control. For instance, given that our control is 11,200 μM hepatic cholesterol, 80% of our control corresponds to 8,960 μM hepatic cholesterol. Similarly, Lakshman *et al.* [8] introduced various doses of dioxin in mice and determined the consequent synthetic rate of cholesterol in the liver.

4. Simulating Dioxin Exposure and Diet in PLAS

The default dietary intake of cholesterol is set at the recommended value of 200 mg/day, which corresponds to 517.26 µmol/day. High cholesterol diet is coded as 400 mg/day or 1,030 µmol/day, while extremely high cholesterol diet is modeled as 600 mg/day = 1,552 µmol/day. To account for diet and dioxin exposure simultaneously, one changes both the parameter value accounting for dietary change, ***Diet***, and the parameter values for the dioxin effects, ***DR_i_*.**

Dietary parameters are set in the computer code as:

- Normal cholesterol diet: 200mg or 0.2g / day ~ 517.26, which is implemented as

dD= (517.26/SF)/ VL

- High cholesterol diet: 0.4g/day ~ 1030
- Extremely high cholesterol diet: 0.6g/day ~ 1552

5. Sensitivity Analysis

### 5.1. Sensitivity analysis for the lipoprotein anchor model

Sensitivity analysis for the lipoprotein anchor model, comprising the transfer of cholesterol between VLDL, IDL, LDL and HDL, and the transport of lipoproteins between hepatocytes, plasma and peripheral tissue, was conducted to quantify the influence of model parameters for LDL, HDL and total plasma cholesterol.

Total plasma cholesterol was calculated as the sum of VLDL, IDL, LDL, and HDL cholesterol steady-state concentrations. The sensitivity analysis was performed numerically by increasing and decreasing one parameter at a time by 10% from the default value and calculating the percentage change of LDL HDL, and total plasma cholesterol. Relative sensitivity coefficients were calculated by averaging the ratios of the percentage change of the cholesterol concentrations to 10% in both directions. The results are shown in (**Fig A**).

Fig A. Sensitivities of plasma cholesterol, LDL, and HDL. All relative sensitivities are below 1 in magnitude.

All parameters have a sensitivity coefficient below 1 in magnitude, with four parameters being notably more sensitive than others: v65, reflecting the transfer of cholesterol from HDL to LDL; v62, reflecting the transfer from HDL to VLDL; v10, reflecting the hepatic removal of cholesterol for excretion; and v57, reflecting the transport of LDL cholesterol to peripheral tissues. These parameters can be considered as comparatively influential, although the relative sensitivities are still below 1 in magnitude. The low sensitivities indicate that inaccuracies in parameter determination are not particularly influential.

### 5.2. Sensitivity analysis of the steroidogenesis pathway model

As described in the body of the article, the anchor model of steroid hormone production addresses estradiol production and utilization throughout the menstrual cycle. For the purposes of an overall long-term health risk assessment, we replaced the oscillations with an average level of estradiol obtained +/- 3 days around the time of ovulation [9], quasi as a long-term steady-state level. This average level represents a value consistent with concentrations commonly referenced in clinical practice [10]. One could use the oscillating model, but we did not see this granularity as necessary. Nonetheless, the substitution with averages demonstrates how different anchor models of a subsystem may be swapped without affecting the operation of other anchors.

Local sensitivity analysis revealed clear differences in the sensitivity of the model to various parameters, although all relative sensitivities were found to be below 1 in magnitude. In particular, we conducted a sensitivity analysis to quantify the influence of model parameters on the length of the follicular phase (**Fig B**). The length of the follicular phase is defined as the duration from the start of the follicular phase to the time of the peak of the LH surge. The sensitivity analysis was performed by increasing and decreasing one parameter at a time by 10% from the default value and calculating the percentage change of the follicular phase length. Relative sensitivity coefficients were calculated by averaging the ratios of the percentage change of the follicular phase length to 10% in both directions.


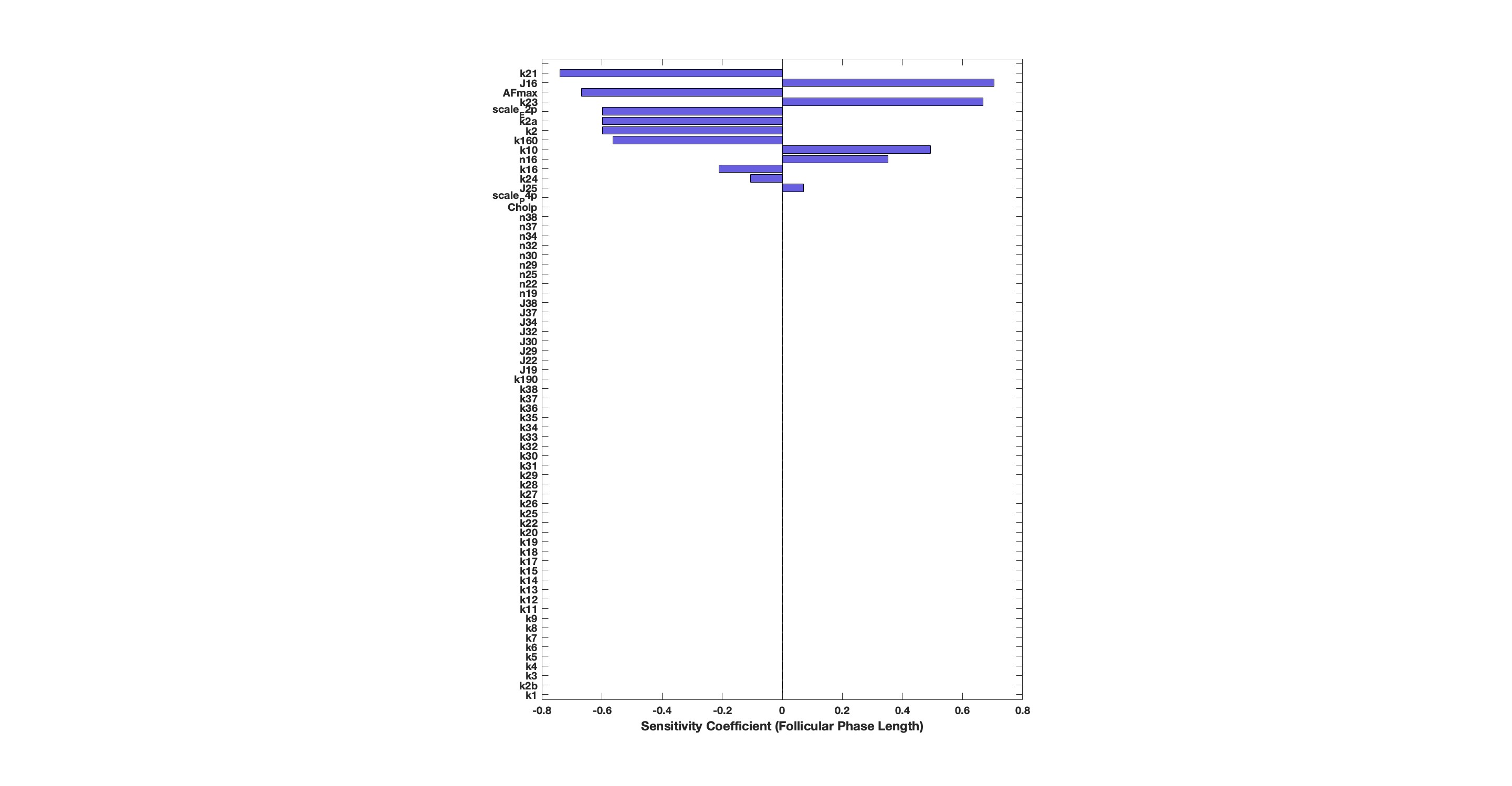


Fig B. Results of a local sensitivity analysis for steroidogenesis pathway anchor model. All parameters have a sensitivity coefficient below 1 in magnitude.

All parameters exhibit sensitivity coefficients of less than ± 0.8. Therefore, the parameters can be considered to exhibit low sensitivity, indicating that inaccuracies in parameter determination are not particularly influential. Of the 66 parameters, 13 parameters have a sensitivity coefficient of greater than ± 0.1. The parameters that exert the most influence on the model are k21, the rate constant reflecting the antral follicle growth rate, and J16, the affinity constant reflecting the E2 threshold that triggers the LH surge.

### 5.3. Sensitivity analysis of the template model

Sensitivity analysis of the template model did not reveal troubling results: All relative parameter sensitivities were below 1 in magnitude, indicating robustness of the model.

As an example, consider the parameters for hepatic cholesterol and plasma cholesterol, which show high connectivity within the template model. The sensitivity analysis was performed by increasing and decreasing one parameter at a time by 10% from the default value and calculating the percentage change of LDL HDL, and total plasma cholesterol. Relative sensitivity coefficients were calculated by averaging the ratios of the percentage change of the cholesterol concentrations to 10% in both directions. The parameters exerting the strongest influence include the rate constant v_01, which quantifies the influx of cholesterol into the liver. The analysis revealed that variations of ±10% in each of the 16 parameters yielded changes in the steady-state values of less than 10% in magnitude (absolute values of relative sensitivity <1; **Fig C**). Therefore, all parameters were deemed to exhibit low sensitivity, thus indicating a robust system response to parameter fluctuations.


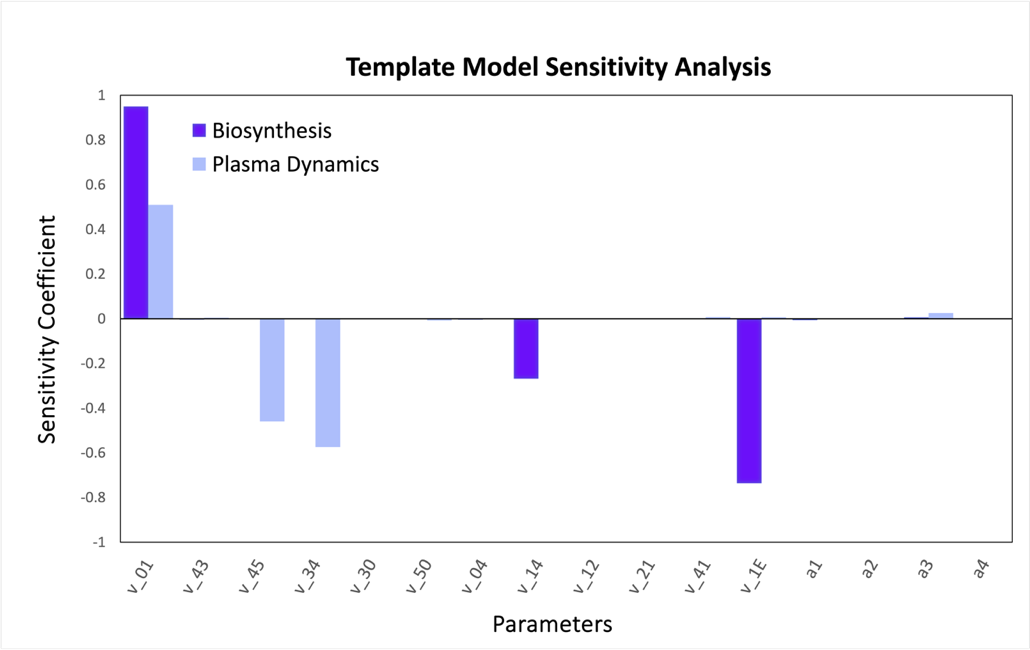


Fig C. Results of a sensitivity analysis of the template model. All parameters values exhibit changes in the steady-state values of less than 10% in magnitude, corresponding to absolute values of relative sensitivities <1.

6. Examples of T&A-like models with processes as variables

### PBPK models. Physiologically based pharmacokinetic (PBPK) models are used to assess the fate of a foreign organic compound within the body. Specifically, they facilitate the simulation of the absorption, distribution, metabolism, and excretion of drugs or toxicants and allow extrapolations from one species to another, typically from rodents to humans [11-13]. PBPK models are structured to represent in a somewhat simplistic manner the actual physiological architecture of the organism and take the format of a hybrid between a compartment model at the organismal level and a set of kinetic models within the compartments. In the example of Fig D, for instance, a drug is inhaled and subsequently migrates throughout the body via the bloodstream.

### What renders PBPK models inherently multiscale is their ability to bridge different levels of biological organization. At the molecular level, they address interactions like drug partitioning into lipids, which are prevalent in fatty tissue, while they account at the cellular level for processes like drug uptake and metabolism, for instance, in the liver. Moving up the scale, they define compartments for specific organs or tissues, each with its distinct drug-related parameters. Collectively, the integrative modeling of these different components enables a holistic view of how a substance behaves across the entire organism, from initial drug administration to eventual elimination, thus offering a comprehensive perspective that spans multiple biological scales and—on the practical side—offers guidance regarding drug dosing and administration regimens.

**Fig D. Generic PBPK model as a multiscale structure.** The primary scale of granularity reflects organs and tissues, but the drug concentrations in these organs are subject to molecular and cellular events, such as sequestration and degradation in the liver. In this illustration, the drug or toxicant enters the body per inhalation.

As an example, consider a typical PBPK model (Fig D), where the variables reflect specific organs or tissues. After inhalation and transport, the drug enters the liver from the bloodstream, is partially degraded by enzymes, partially stored, and partially released into bile or again into the bloodstream. Suppose now that some perturbation, such as exposure to some toxicant, alters the enzymatic activities within the liver. This perturbation results in an altered distribution between degradation, storage, and release. The overall change in output, that is, release of the drug into the bloodstream, caused by the toxicant, informs the PBPK model under exposure.

Considering the set-up and functionality of a PBPK model, one realizes that it has, in some sense, the structure of a T&A model, even though it seems that this connection has not yet been made in the literature. Namely, at first glance, the variables appear to be organs, but a moniker like “Liver” is misleading: The variable “Liver” in truth means the concentration of a drug or toxicant being processed by the liver at some point in time. Importantly, the quantity (numerical value) of this variable is not only driven by the influx of the drug from the arterial and portal blood and its efflux into the venous blood but also by the enzymatic, transport, and storage activities that alter a portion of this drug within the organ by means of degradation, excretion, or binding. Without accounting for these processes within the given box (*i.e.*, variable), there is no valid flux balance at a steady state. This situation is reconciled if one recognizes that the variable “Liver” represents a system of processes in which the observed influx is converted into the observed efflux. Thus, this situation of a PBPK model is typical for a template model, whose anchor “Liver” is a lower-level template that contains subsystems of uptake, degradation, excretion, storage, and other processes. At the same time, each apparent flux (edge) in the PBPK model in truth represents the concentration of the drug or toxicant being transported from one organ to another.

*Cellular Physiology.* Suppose a high-level template represents some aspect of cellular physiology. Its anchors might represent mitochondrial energy handling, protein synthesis, generation of reactive oxygen species, and a feature like cell contraction. Interestingly, these anchors have the structure of template models, as they represent systems of processes, rather than processes whose variables are biological entities like metabolites. Thus, the T&A model is extended to more thatn two levels. In typical T&A models, the variables of a template models might be true anchor models, which are usually not further reducible to sub-models, or they are themselves lower-level template models with variables that may either be further templates or true anchors. For some psychological reasons, we tend to think downward, that is, from a template to its anchors. Here, it might be more instructive to start with pure anchors at the lowest level of interest, which become the variables of templates at a higher level, which in turn are integrated in template models at even higher levels until one reaches an overarching umbrella template.

*Flux Balance Analysis (FBA).* FBA is widely used in metabolic engineering to increase the microbial production of some organic compound that it is either valuable and difficult to obtain (insulin; some amino acids) or needs to be produced cheaply in large quantities (industrial ethanol; citric acid) [14]. The specific goal of FBA is a targeted redistribution of metabolic fluxes through constrained optimization. Of note is that the variables of FBA are metabolic fluxes, that is, processes of converting one metabolite into another, or transport processes. The entire optimization analysis is executed with fluxes, and the results do not state anything regarding metabolite concentrations.

**References**

1. Full, R.J. and D.E. Koditschek, *Templates and anchors: neuromechanical hypotheses of legged locomotion on land.* J Exp Biol, 1999. **202**(Pt 23): p. 3325-32.

2. Kumbale, C.M., Q. Zhang, and E.O. Voit, *Hepatic cholesterol biosynthesis and dioxin-induced dysregulation: A multiscale computational approach.* Food and Chemical Toxicology, 2023: p. 114086.

3. Savageau, M.A., *Biochemical systems analysis. I. Some mathematical properties of the rate law for the component enzymatic reactions.* J Theor Biol, 1969. **25**(3): p. 365-9.

4. Voit, E., *A First Course in Systems Biology (2nd ed.). Garland Science*. 2017.

5. Voit, E.O., *Computational Analysis of Biochemical Systems: A Practical Guide for Biochemists and Molecular Biologists*. 2000, Cambridge, U.K.: Cambridge University Press.

6. Voit, E.O., *Biochemical Systems Theory: A Review.* ISRN Biomathematics, 2013. **2013**: p. 897658.

7. Voit, E.O., H.A. Martens, and S.W. Omholt, *150 years of the mass action law.* PLoS Comput Biol, 2015. **11**(1): p. e1004012.

8. Lakshman, M.R., et al., *Effects of 2,3,7,8-tetrachlorodibenzo-p-dioxin (TCDD) on de novo fatty acid and cholesterol synthesis in the rat.* Lipids, 1988. **23**(9): p. 904-6.

9. Guber HA, O.M., Russell YX, *Evaluation of endocrine function.*, in *Henry's Clinical Diagnosis and Management by Laboratory Methods.*, P.M. McPherson RA, eds., Editor. 2022, Elsevier: St Louis, MO.

10. Mount Sinai. *Estradiol blood test*. 2023; Available from: <https://www.mountsinai.org/health-library/tests/estradiol-blood-test>.

11. Andersen, M.E., *Physiologically-based pharmacokinetic modeling.* Drug Information Journal, 1994. **28**(1): p. 247-254.

12. Campbell JL Jr, et al., *Physiologically based pharmacokinetic/toxicokinetic modeling.* Computational Toxicology - Methods in Molecular Biology, 2012. **929**: p. 439–499.

13. Liu, R., et al., *A Physiologically Based Pharmacokinetic (PBPK) Modeling Framework for Mixtures of Dioxin-like Compounds.* Toxics, 2022. **10**(11).

14. Orth, J.D., I. Thiele, and B.Ø. Palsson, *What is flux balance analysis?* Nature Biotechnology, 2010. **28**(3): p. 245-248.

**Figure Legends**

**Fig A. Sensitivities of plasma cholesterol, LDL, and HDL.** All relative sensitivities are below 1 in magnitude.

**Fig B. Results of a local sensitivity analysis for steroidogenesis pathway anchor model.** All parameters have a sensitivity coefficient below 1 in magnitude.

**Fig C. Results of a sensitivity analysis of the template model.** All parameters values exhibit changes in the steady-state values of less than 10% in magnitude, corresponding to absolute values of relative sensitivities <1.

**Fig D. Generic PBPK model as a multiscale structure.** The primary scale of granularity reflects organs and tissues, but the drug concentrations in these organs are subject to molecular and cellular events, such as sequestration and degradation in the liver. In this illustration, the drug or toxicant enters the body per inhalation.
