# Supplementary material for: Analysis of systemic effects of dioxin on human health through template-and-anchor modeling
Source: PLoS Comput Biol. 2025 Mar 27;21(3):e1012840. doi: 10.1371/journal.pcbi.1012840 (PMC12005559; doi:10.1371/journal.pcbi.1012840)
Supplement: Attachment — Submitted filename: PCOMPBIOL-D-24-00128_reviewer.pdf [file pcbi.1012840.s002.pdf]

PLOS Computational Biology

Multiscale Biomedical Systems Analysis through Template-and-Anchor Modeling

--Manuscript Draft--

**Manuscript Number:**	PCOMPBIOL-D-24-00128
**Full Title:**	Multiscale Biomedical Systems Analysis through Template-and-Anchor Modeling
**Short Title:**	Multiscale Biomedical Systems Analysis
**Article Type:**	Research Article
**Keywords:**	Dioxin; Dose-Response-Curve; Multiscale Modeling; PBPK Model; Template-and-Anchor Model; Time Scale Separation
**Abstract:**	We address the well-known challenge of biomedical multiscale analysis with a novel adaptation of the Template-and-Anchor (T&A) modeling paradigm. We consider a template as a high-level, coarse-grained model that focuses exclusively on the main physiological components of a system and involves correspondingly few variables, processes, and parameters. Anchor models are modules of component sub-systems that provide more elaborate descriptions of specific biological details. This conceptual framework does not attempt to capture simultaneously all details within a single computable structure as many other multiscale models do. Instead, the often-overwhelming multilevel task is dissected into smaller, stand-alone models that are analyzed separately. This new adaptation of the T&A approach offers substantial advantages without losing resolution. First, the divide-and-conquer method enhances computational efficiency. Second, unlike other methods, each anchor is analyzed individually, producing a record of crucial input-output relationships that are ultimately used in the template model. Third, anchor models can be interchanged without affecting the structure of the template or other anchors. Fourth, the T&A model provides flexible guidelines for its setup and for defining variables across scales. Using this approach, we investigate the health implications of exposure to 2,3,7,8-tetrachlorodibenzo-p-dioxin (TCDD), a persistent organic pollutant which can severely affect health, depending on the magnitude of exposure. We present a template capturing the overall effects of TCDD on the dynamics of cholesterol, a prominent target of TCDD. We then briefly highlight one representative anchor model representing cholesterol handling by hepatocytes. The eventual creation of all anchor models and the template will enable a holistic evaluation of dioxin's impact on the liver, which can be translated into a comprehensive computational health risk assessment tool. Overall, multiscale modeling, and more specifically T&A modeling, holds promise for a deeper understanding of complex systems and for advancing personalized medicine, interspecies translation, and virtual clinical trials.
**Additional Information:**	
**Question**	**Response**
**Government Employee**<br><br>Are you or any of the contributing authors an employee of the United States government?<br><br>Manuscripts authored by one or more US Government employees are not copyrighted, but are licensed under a CC0 Public Domain Dedication, which allows unlimited distribution and reuse of the article for any lawful purpose. This is a legal requirement for US Government	No - No authors are employees of the U.S. government.

Powered by Editorial Manager® and ProduXion Manager® from Aries Systems Corporation

employees.<br><br>This will be typeset if the manuscript is accepted for publication.	
**Financial Disclosure**<br><br>Enter a financial disclosure statement that describes the sources of funding for the work included in this submission. Review the submission guidelines for detailed requirements. View published research articles from *PLOS Computational Biology* for specific examples.<br><br>This statement is required for submission and **will appear in the published article** if the submission is accepted. Please make sure it is accurate.<br><br>**Funded studies**<br>Enter a statement with the following details:<br>• Initials of the authors who received each award<br>• Grant numbers awarded to each author<br>• The full name of each funder<br>• URL of each funder website<br>• Did the sponsors or funders play any role in the study design, data collection and analysis, decision to publish, or preparation of the manuscript?<br><br>Did you receive funding for this work?	Yes
Please add funding details.<br>as follow-up to "**Financial Disclosure**<br><br>Enter a financial disclosure statement that describes the sources of funding for the work included in this submission. Review the submission guidelines for detailed requirements. View published research articles from *PLOS Computational Biology* for specific examples.<br><br>This statement is required for submission and **will appear in the published article** if the submission is accepted. Please make	NIEHS HERCULES grant P30ES019776-05 (PI: Carmen Marsit)<br><br>NIEHS Superfund Research grant P42ES04911 (PI: Norbert Kaminski)

sure it is accurate.<br><br>**Funded studies**<br>Enter a statement with the following details:<br>• Initials of the authors who received each award<br>• Grant numbers awarded to each author<br>• The full name of each funder<br>• URL of each funder website<br>• Did the sponsors or funders play any role in the study design, data collection and analysis, decision to publish, or preparation of the manuscript?<br><br>Did you receive funding for this work?"	
Please select the country of your main research funder (please select carefully as in some cases this is used in fee calculation).<br>as follow-up to "**Financial Disclosure**<br><br>Enter a financial disclosure statement that describes the sources of funding for the work included in this submission. Review the submission guidelines for detailed requirements. View published research articles from *PLOS Computational Biology* for specific examples.<br><br>This statement is required for submission and **will appear in the published article** if the submission is accepted. Please make sure it is accurate.<br><br>**Funded studies**<br>Enter a statement with the following details:<br>• Initials of the authors who received each award<br>• Grant numbers awarded to each author<br>• The full name of each funder<br>• URL of each funder website<br>• Did the sponsors or funders play any role in the study design, data collection and analysis, decision to publish, or preparation of the manuscript?<br><br>Did you receive funding for this work?"	UNITED STATES - US

Powered by Editorial Manager® and ProduXion Manager® from Aries Systems Corporation

**Competing Interests**<br><br>On behalf of all authors, disclose any competing interests that could be perceived to bias this work.<br><br>This statement will be typeset if the manuscript is accepted for publication.<br><br>Review the instructions link below and PLOS Computational Biology's competing interests policy to determine what information must be disclosed at submission.	The authors declare no competing interests.
**Data and Code Availability**<br><br>From the time of publication, Authors are required to make fully available and without restriction all data and computational code underlying their findings. Please see our PLOS Data Policy page for detailed policy information, and our Code Sharing page for specific information on code sharing.<br><br>A **Data Availability Statement**, detailing where the data (and code, if applicable) can be accessed, is required at first submission. Insert your Data Availability Statement in the box below. The statement you provide **will be published in the article**, if accepted.<br><br>Please see the Data Reporting section of our submission guidelines for instructions on what you need to include in your Data Availability Statement.<br><br>If the data and code are all contained in your submission files, please state: *All relevant data are within the manuscript and its Supporting Information files.*<br><br>**PLOS allows rare exemptions to address legal and ethical concerns.** If you have legal or ethical restrictions, please detail these in your Data Availability Statement	No data used.<br>Code will be made available on GitHub.

Powered by Editorial Manager® and ProduXion Manager® from Aries Systems Corporation

below for the Journal team to consider.	

Powered by Editorial Manager® and ProduXion Manager® from Aries Systems Corporation

Click here to access/download;Manuscript;TA PLOS.111.docx

Multiscale Biomedical Systems Analysis through Template-and-Anchor Modeling

Carla M. Kumbale*, Qiang Zhang, and Eberhard O. Voit*,*****

* Department of Biomedical Engineering, Georgia Institute of Technology, Atlanta, GA 30332, USA

** Gangarosa Department of Environmental Health, Rollins School of Public Health, Emory University, Atlanta, GA 30322, USA

*** Corresponding author

Email Addresses

Ckumbale3@gatech.edu

Qiang.zhang@emory.edu

Eberhard.voit@bme.gatech.edu

Key Words: Agent-Based Models, Dioxin, Dose-Response-Curve, Multiscale Modeling, PBPK Model, Template-and-Anchor Model, Time Scale Separation

Abstract

We address the well-known challenge of biomedical multiscale analysis with a novel adaptation of the *Template-and-Anchor* (T&A) modeling paradigm. We consider a template as a high-level, coarse-grained model that focuses exclusively on the main physiological components of a system and involves correspondingly few variables, processes, and parameters. Anchor models are modules of component sub-systems that provide more elaborate descriptions of specific biological details. This conceptual framework does not attempt to capture simultaneously all details within a single computable structure as many other multiscale models do. Instead, the often-overwhelming multilevel task is dissected into smaller, stand-alone models that are analyzed separately. This new adaptation of the T&A approach offers substantial advantages without losing resolution. First, the divide-and-conquer method enhances computational efficiency. Second, unlike other methods, each anchor is analyzed individually, producing a record of crucial input-output relationships that are ultimately used in the template model. Third, anchor models can be interchanged without affecting the structure of the template or other anchors. Fourth, the T&A model provides flexible guidelines for its setup and for defining variables across scales. Using this approach, we investigate the health implications of exposure to 2,3,7,8-tetrachlorodibenzo-p-dioxin (TCDD), a persistent organic pollutant which can severely affect health, depending on the magnitude of exposure. We present a template capturing the overall effects of TCDD on the dynamics of cholesterol, a prominent target of TCDD. We then briefly highlight one representative anchor model representing cholesterol handling by hepatocytes. The eventual creation of all anchor models and the template will enable a holistic evaluation of dioxin's impact on the liver, which can be translated into a comprehensive computational health risk assessment tool. Overall, multiscale modeling, and more specifically T&A modeling, holds promise for a deeper understanding of

complex systems and for advancing personalized medicine, interspecies translation, and virtual clinical trials.

Author Summary

Biological systems are organized in distinct but connected layers, which makes their analysis a challenge. The higher layers usually correspond to a “big picture” of physiological events, whereas the lower levels account for increasing granularity and detail. It is infeasible to carry along all details from lower levels, partly for technical reasons, but also because they would overwhelm insights at the higher level due to their sheer numbers and the fact that they typically run on much faster time scales. We propose to address this situation with an innovative Template-and-Anchor (T&A) modeling approach, where the template model represents a system of processes at some fixed level of biological organization and its variables serve as more finely grained anchor models. As an illustrating example, we analyze the effects of dioxin on human health and, especially, on the dynamics of cholesterol handling. At first glance, the structures of template and anchor models seem to be simple variations of the same theme, but they relate to each other somewhat like primal and dual graphs: The variables of the anchor models are regular variables, such as cholesterol, whereas the variables of the template are processes, such as cholesterol biosynthesis. We discuss the consequences of this difference.

Introduction

Biological phenomena occur and interact across multiple levels of organization, from molecules and cells to tissues, organs, organisms, and ecosystems. Each level is intricately connected and

may influence others, making the study of these multiscale systems complex and challenging. As a generic example, molecular interactions govern the behavior of proteins, nucleic acids, lipids, and other biological macromolecules. These molecular interactions manifest in cellular functions such as metabolic processes, ligand binding and signal transduction. At the tissue level, cells interact through specific characteristics and play crucial roles in phenomena such as hormonal signaling, which governs many aspects of physiology. Thus, the collective behaviors of the various molecular processes within and among cells affect tissues, organs, organ systems and ultimately organisms that respond adequately to their environments.

Further complexity arises if one considers the interplay among different cell types, tissues, and organs, where a solid understanding of these relationships is key to deciphering biological processes such as digestion, circulation, and disease, to name just a few [1]. The intricacies of these processes are not only manifest in individual multi-cellular organisms, but quickly arise if one considers the interactions between organisms, populations, and entire ecosystems, wherein interactions among different environments and species introduce even greater complexity. These interactions in turn influence numerous phenomena, from population growth to the spread of infectious diseases and environmental changes.

The often-encountered multilevel complexity implies that traditional reductionist methods alone do not provide a comprehensive understanding of biomedical phenomena and mandates an integrated approach that takes into account interactions and dependencies across multiple scales [1-3]. For example, unraveling diseases such as cancer requires knowledge of how molecular-level interactions translate into higher-level effects within the body. Accordingly, cancer is often rightfully described as a systemic disease, due to the intricate interplay of factors at multiple levels that contribute to its onset and progression [4]. Namely, while cancer is known to be

characterized by ill-regulated cell proliferation and the invasion into healthy tissues, its manifestation and behavior are influenced by a vast network of cellular and physiological interactions, such as immune responses. Furthermore, tumors are heterogeneous cellular assemblies, whose growth and metastatic potential depend upon dynamical interactions among the cancer cells themselves and their constantly changing microenvironment [5, 6]. Additional systemic factors, such as hormonal responses, have profound effects on the behavior of cancers [7]. This complex web of interactions, which spans from the molecular to the organismal level, necessitates an integration of diverse processes on multiple levels for a better understanding and treatment of this disease [8].

Not surprisingly, substantial challenges exist for the design and development of computational models describing multiscale phenomena [9]. The fundamental reason is the vastly increased number of components at various scales, both temporally and spatially, and the need to define, quantify, and often adjust numerous parameters of different types that possibly interrelate with one another across scales. As a part of this challenge, defining the linkage between different scales is a substantial hurdle. Often, these links are bidirectional, implying that variables, parameters, and functions characterizing the biomedical system influence one another [10]. The new complexities associated with multiscale models confound the challenges already occurring when large numbers of variables, nonlinear interactions and emergent behaviors are at play on each level [11]. These challenges are manifest in computational demands, validation, and standardization. Simulating the dynamics across various scales can be computationally intensive, requiring specialized algorithms [12, 13]. A separate issue is the validation of these models, which may need unique criteria at each level, thereby potentially leading to compounded

uncertainties. Finally, the lack of standardized methodologies across different domains complicates the analysis, comparison, and interpretation of multiscale systems [14].

Multiscale Modeling Approaches

In an overview on multiscale modeling in biology [1], the authors discuss the scientific progression and advances in scientific tools and technologies that have enabled the research community to explore complex biological systems that are inherently multi-scaled in nature. Future progress will require not only advancements of these methods, but also transdisciplinary collaboration among researchers in molecular and cellular biology, physiology, evolution, ecology, mathematics, bioinformatics, machine learning and what is now called data science [15, 16]. This collaboration is needed not just to obtain, organize, and analyze the massive volumes of data generated through experimentation, but for constructing, improving, and standardizing comprehensive models that can elucidate the functioning of life across different temporal and spatial scales. With this mandate for collaboration comes the need for improved transdisciplinary communication (Chapter 19 of [17]).

Numerous structured and *ad hoc* approaches to multiscale modeling have been proposed to capture the intricate interactions and phenomena occurring across different scales of biological systems. Two very different yet representative examples are physiologically based pharmacokinetic (PBPK) models and agent-based models (ABM).

Physiologically based pharmacokinetic (PBPK) models simulate the absorption, distribution, metabolism, and excretion of drugs or chemicals within living organisms (**Fig 1**). These models are structured to represent in a somewhat simplistic manner the actual physiological architecture

of the organism and take the format of a hybrid between a compartment model at the organismal level and a set of kinetic models within the compartments [18, 19]. What renders PBPK models inherently multiscale is their ability to bridge different levels of biological organization. At the molecular level, they address interactions like drug-lipid partitioning, as it occurs in fatty tissue, while they account at the cellular level for processes like drug uptake and metabolism, for instance, in the liver. Moving up the scale, they define compartments for specific organs or tissues, each with its distinct drug-related parameters. Collectively, the integrative modeling of these different components enables a holistic view of how a substance behaves across the entire organism, from initial drug administration to eventual elimination, thus offering a comprehensive perspective that spans multiple biological scales and—on the practical side—offers guidance regarding drug doses and administration regimens.

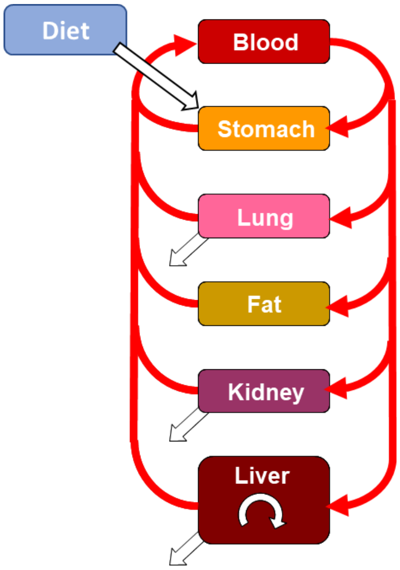

Fig 1: Generic PBPK model as a multiscale structure. The primary scale of granularity reflects organs and tissues, but the drug concentrations in these organs are subject to molecular and cellular events, such as sequestration and degradation in the liver.

The second example is an agent-based model (ABM), which is a generic stochastic approach that simulates the actions and interactions of autonomous agents, which are individual entities with distinct attributes and behavior[18, 20-22] The agents typically move on a grid and can represent a myriad of entities, ranging from molecules and cells in biological systems to individuals in social scenarios. Governed by predefined rules, ABM agents can adapt, learn, and evolve based on their experiences and interactions within their environment. This modeling approach is inherently multiscale because it can span a wide range from the micro-level where, for instance, agents might represent cells or individuals, to the macro-level, where tissue function or societal dynamics emerge. The temporal aspect of ABMs can capture both immediate agent decisions and long-term system evolutions. Further, ABMs can involve spatial differences and capture behaviors that arise from numerous individuals dynamically interacting according to preset collective properties [23, 24]. More generally, multiscale modeling often adopts a “hybrid” approach in which the actions of migrating agents may be governed by systems of differential equations, which are updated at every discrete step of the ABM [25-27]. Several other types of hybrid models have been discussed in the literature [28-30].

Technical Challenges of Multiscale Modeling

The organization of biological systems as distinct but connected layers poses one of the grand challenges for biomathematical modeling, because processes occurring at the various layers often have different time scales and almost always focus on different types of variables. The higher layers usually correspond to a “big picture” of physiological events, whereas the lower levels account for increasing granularity and detail. When investigating a system at a high level, it is often infeasible to carry along all details from lower levels, partly for technical reasons, but also because they would overwhelm insights at the higher level due to their sheer numbers and the

fact that they typically run on much faster time scales. For instance, the growth of an embryo from a fertilized oocyte throughout the phases of blastula, gastrula and further development may span several weeks, during which uncounted metabolic and cellular processes occur at a much more rapid pace.

A partial solution that was proposed a long time ago is time-scale separation, which yields a reduction in model size (*e.g.*, [31]). As an explanation for the validity of this reduction, it has been argued (*e.g.*, [32, 33]) that the processes at lower levels are so fast that they are essentially always in a quasi-steady state, which permits their conversion from differential equations to explicit algebraic equations that may be entered as constraints in a much smaller set of governing equations. As an example, the clouding of the human lens is a very slow, life-long process that is caused by clearly identifiable biochemical reactions that occur on an ongoing basis at the sub-second scale. In this case, the biochemical aspects may be analyzed separately from the slow clouding process during aging [34]. While separation of time scales is certainly an option for processes with time constants at different orders of magnitude, such a separation is not feasible if the layers of biological organization are not all that distant. For instance, the dynamics of calcium exchanges in the heart is fast in comparison to macroscopic heart physiology, but it is close to impossible to separate the two [35]. In another example, discussed later in detail, the direct effects of dioxin exposure on human health are manifest at the transcriptional and metabolic levels, which have similar time scales, although their consequences may, over the span of years, lead to liver disease, reproductive dysfunction, and possibly cancer.

Template and Anchor (T&A) Modeling:

To address the various challenges inherent in multiscale modeling, we propose a modeling paradigm suggested over two decades ago, but seldom actually implemented, namely Template-and-Anchor (T&A) modeling [36]. A template is a high-level model that focuses exclusively on the main physiological components of a system and involves correspondingly few variables, processes, and parameters. It provides a coarse-grained representation of the system under investigation. Anchor models provide more elaborate views of the specific biological details characterizing the mechanisms that govern the system and are represented in the template model as variables.

The original authors and proponents of T&A modeling, Full and Koditschek [36], considered this approach most useful not for multiscale modeling *per se*, but for the embedding of a generic design principle into a space of alternate implementations. Expressed differently, they considered the template model as a design principle and the anchor models as differently structured implementations of this common design. Their key example assessed the locomotion of terrestrial animals, which the authors demonstrated to have numerous, quite different manifestations (which they called “degrees of freedom”), such as the number and structure of legs. While distinctly different, all implementations had to comply with a common det of biomechanical design features. Kvalheim and Revzen [37] formally investigated this aspect of the T&A approach from a standpoint of control theory. They embedded all anchors in a high-dimensional space such that their intersection consisted of the low-dimensional template, which was thereby seen as the common design principle that all implementations had to satisfy. As the authors expressed it, “the template is not only a description of a typical subset of poses [of a

galloping horse], but also a low-dimensional dynamical model that captures features of the aggregate behavior of the body."

Our focus here is not on an embedding of different implementations of a design feature but on a method for reducing complexity by replacing anchor models with input-output relationships that later inform the template model. Specifically, we consider the template model as a representation of processes at some fixed level of biological organization. As is typical in dynamic modeling, this template contains variables that interact dynamically and are formulated as explicit functions with appropriate parameters. The model may be assessed with the usual steady-state analyses (*e.g.*, characterizing stability and sensitivities), as well as with computational simulations. Of importance for the T&A approach, the variables of the template model, when zoomed in, are in themselves more finely grained models, which serve as anchor models. The anchor models are analyzed separately across applicable scenarios, and the main outcome of each anchor analysis is a functional or tabulated record of all pertinent input-output relationships, which are subsequently used to connect to other anchors and to calibrate the template model. In the end, the template model can be simulated without simultaneously simulating the anchor models while retaining and utilizing pertinent results from all anchors. This strategy of "logistic level separation" is different from typical multiscale models that attempt to represent all information at once and carry it forward in some fashion.

Example

It is beneficial for further discussions to introduce the main example of this analysis, the effect of TCDD (2,3,7,8-tetrachlorodibenzo-p-dioxin) on human health and, especially, on the dynamics

of cholesterol and the sequelae of exposure. TCDD is known to affect the expression of several genes associated with cholesterol biosynthesis in a process mediated by the xenobiotic aryl hydrocarbon receptor AhR [38, 39]. The study of dioxin is important due to widespread processes generating dioxins as a by-product, such as municipal waste incineration and wildfires, which have resulted in about every person worldwide to be exposed to dioxins to some degree [40]. The problem is confounded by dioxin's long biological half-life, which causes it to remain and gradually accumulate in human tissues for several decades after exposure [41].

Several options are feasible to structure this problem space as a T&A system. A typical template, specifically focusing on the cholesterol handling by various organ systems in the human body, is shown in **Fig 2.** The boxes are the dependent variables of the template model, while the arrows indicate material fluxes among them. Ultimately, each box will be modeled separately as an anchor model.

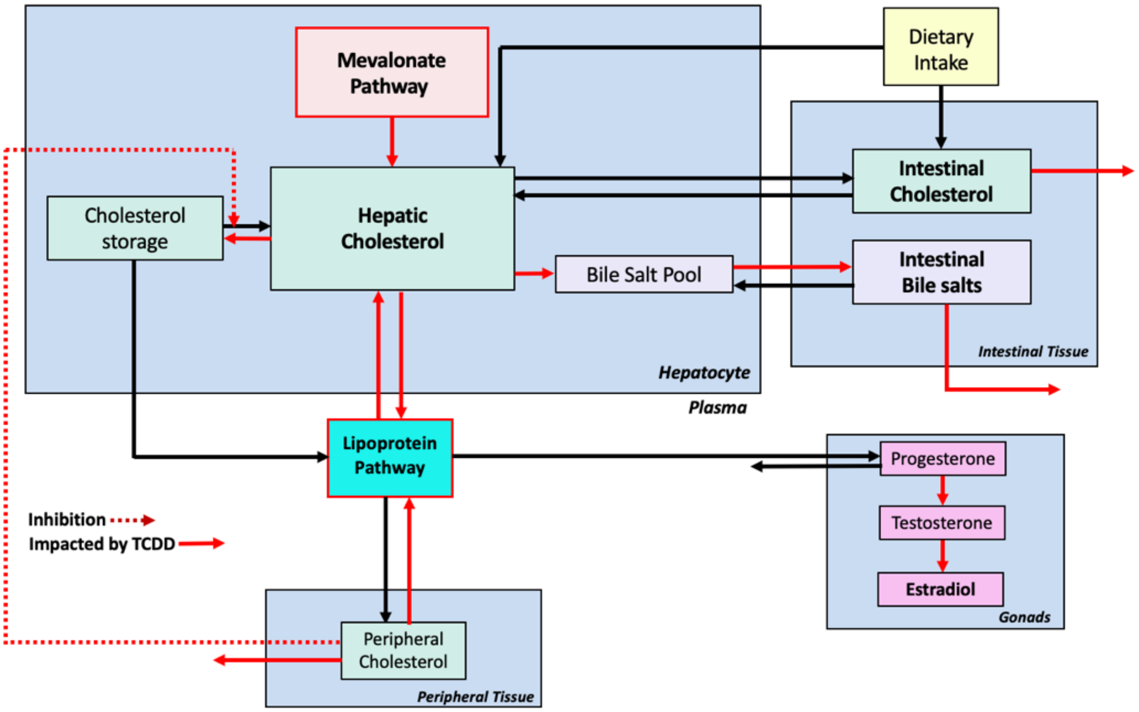

Fig 2: Template model of TCDD exposure affecting cholesterol dynamics. The main focus here is on the mevalonate pathway, which synthesizes cholesterol *de novo*. Processes affected by TCDD exposure are indicated by red arrows. Adapted from [42].

As an illustration, we consider one anchor model, namely the mevalonate pathway in the liver, which is the main source of endogenous cholesterol. It is depicted in **Fig 3.** This anchor is particularly important because several steps within the mevalonate pathway are affected by dioxin exposure.

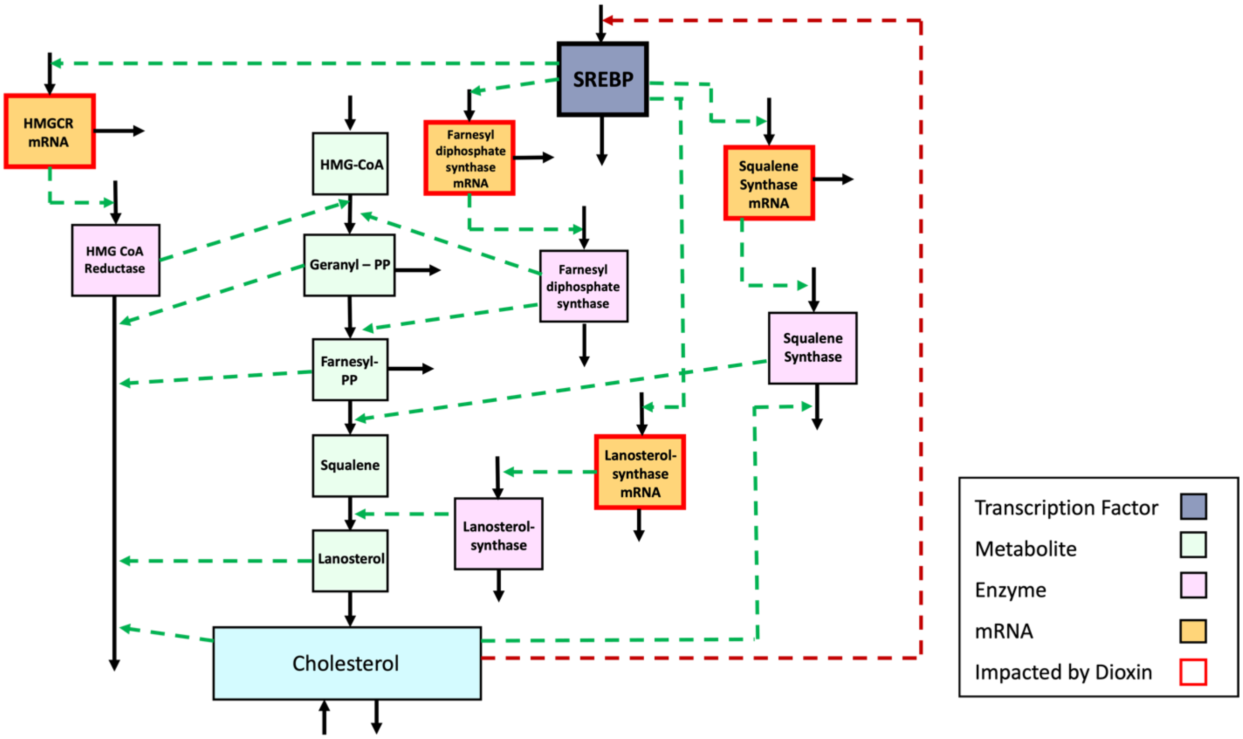

Fig 3: Hepatic cholesterol biosynthesis serves as a representative anchor model. The various components of the biosynthetic mevalonate pathway system are color-coded by type, as indicated. Metabolic reactions, transport steps, and the production of mRNAs and proteins are shown as solid lines, while regulatory signals, representing feedback inhibition (red) and modulation of enzyme activity [43], are given as dotted lines; mRNAs whose transcription is affected by dioxin, are indicated by boxes outlined in red. The key transcription factor SREBP binds to sterol regulatory elements (SREs) in the promoter regions of its target genes. It controls the activity of mevalonate pathway together with HMG CoA (3-hydroxy-3-methyl-glutaryl-coenzyme A) reductase, which is considered the rate-limiting step of the pathway, even though control of the pathway is distributed. Adapted from [42].

This anchor model contains as variables the metabolites of the pathway, as well as pertinent mRNAs and the important transcription factor SREBP, which is controlled by the amount of hepatic cholesterol through feedback inhibition. It is straightforward to set up a metabolic model for this system (see, *e.g.*, [44-46]). Literature searches directly or indirectly yielded parameter values, as

well as information quantifying the effect of TCDD. The details of this model design and calibration methods were presented elsewhere [45, 46] .

Various simulation scenarios are possible with T&A models. For instance, one can simulate different magnitudes of TCDD exposure, analyze the effects of varying dietary cholesterol intake on the system, and test how these variations, combined with TCDD exposure, impact hepatic and serum cholesterol levels [42]. As an illustration, we present below one model result of the analysis of different TCDD concentrations in the format of a dose-response relationship between TCDD and the anchor's output variable, hepatic cholesterol (**Fig 4**).

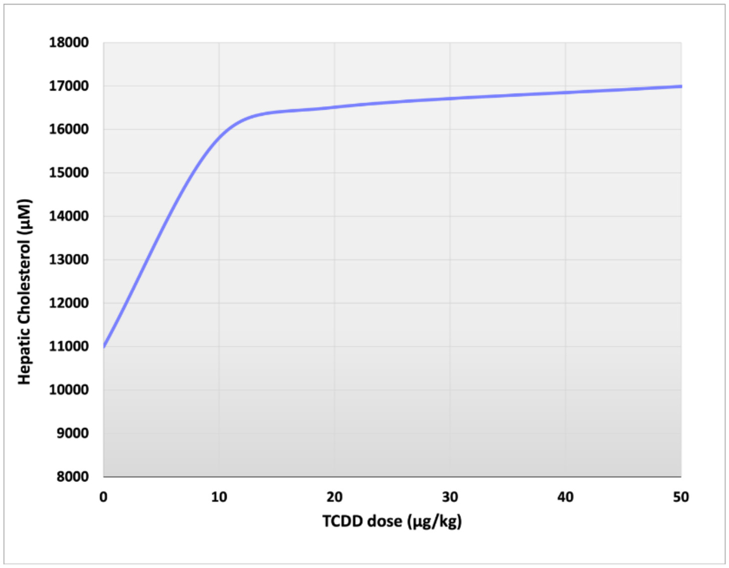

Fig 4: Simulation results with the model are formulated as a dose-response relationship characterizing the effect of TCDD on the amount of hepatic cholesterol produced by the mevalonate pathway. Adapted from [42].

The amount of cholesterol produced under continuous exposure to any relevant TCDD dose of interest can be extracted from this dose-response relationship and will eventually be handed off to

other variables of the template model. Specifically, the dose-response relationship will be used as a TCDD-dependent modulator of the healthy baseline flux (TCDD = 0) between the mevalonate entering the pathway and hepatic cholesterol level (see **Fig 2**).

In the future, other anchor models presented in the template model will be developed, such as a reproductive endocrine model. Substituting all variables of the template model with output information from these anchor models will ultimately allow us to assess the global effects of dioxin exposure on the human body, which has seldom been done before. Given adequate input data from individuals, the T&A approach will permit a personalized human health risk assessment.

Advantages of T&A Modeling

If sufficient data are available, the T&A approach offers substantial advantages without losing much resolution. First, the divide-and-conquer approach greatly increases computational efficiency, as the anchors are analyzed with methods devised for their specific levels; in other words, problems of data heterogeneity are minimized. Second, since anchor models can be designed and analyzed in isolation, the T&A structure can be developed gradually by involving output information from increasingly more anchors, while ill-defined components of the template remain as simple variables. Thus, the approach offers flexible guidance for setting up the model and for the definition of variables and processes at the different stages of the modeling process. Third, in contrast to other multiscale approaches, anchors are not repeatedly analyzed together with other anchors and the template. Instead, each anchor is analyzed separately for all applicable scenarios and the main output consists of a functional or tabular record of all pertinent input-output relationships, which are later

used to inform other anchors and to calibrate the template model. Fourth, the anchor models can be "hot-swapped" in a sense that they may be replaced, if necessary, with more detailed models, while other anchors and the template remain intact.

Theoretical Considerations Regarding the Structure of T&A Models

We have been using T&A models in a straightforward manner, according to the intuitive notion of a template model as a high-level, coarse-grained umbrella structure and the anchors as models elucidating parts of the template in greater detail. At first glance, the mathematical structures of template and anchor models seem to be simple variations of the same theme. Closer inspection, however, reveals an interesting combination of differences and correspondences between template and anchor models in general. This combination is most easily seen for a dynamic system operating at a steady state. At this prominent state, the two types of models have the structures of a regular directed graph (anchor model) and an edge-to-vertex dual graph (template model), respectively [47]. In the language of graph theory, the dual graph G* of a primal (line) graph G is constructed by exchanging edges and vertices. For directed graphs, which are most pertinent here, the vertex set of G* corresponds to the edge set of G, and every directed edge in G* corresponds to an instance in G where the head of an edge e_i meets the tail of another edge e_j. **Fig 5** shows a generic example.

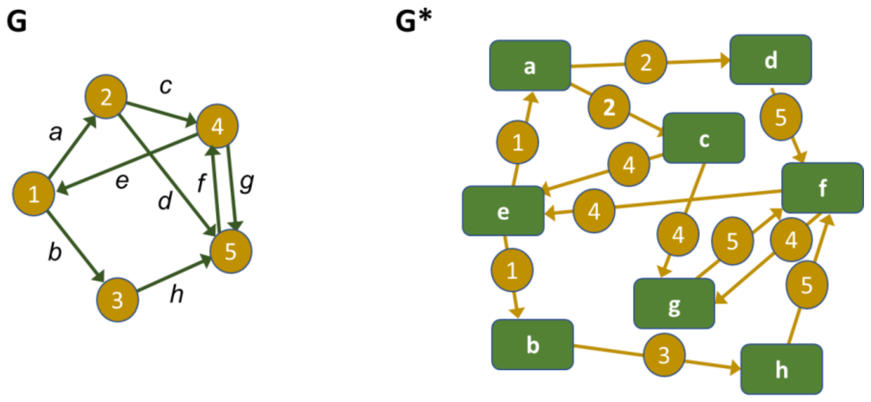

Fig 5: Example of a directed line graph G and its (edge-to-vertex) dual graph G*. The vertices of G* correspond directly to the edges in G, as indicated by letters a – h. The edges of G* correspond to vertices in G if one edge is entering this vertex and another one is leaving it. For instance, vertex 2 in G is represented in the directed dual graph G* through the edges from a to c and from a to d, because these edges meet head to tail at vertex 2 in G.

Graph theory has been discussing different types of graph duality for a long time [47]. In contrast, the literature on dual dynamic models is scattered, with rather different concepts using the term "dual" (*e.g.*, [48-50]). Closest in spirit to the situation of T&A models is an article on flux duality [51], where the exchange of variables and fluxes in certain types of primal and dual differential equation models was investigated.

A typical anchor model has the underlying graph structure of a common metabolic or physiological model. For instance, the variables (vertices) of the cholesterol biosynthesis model (**Fig 3**) are metabolites, and the fluxes (directed edges) among them represent (regulated) biochemical reactions, transport steps, or the formation or disassociation of complexes. This type

of diagram represents the biosynthesis anchor well and, as a natural consequence of the model design, there is perfect flux balance at steady state: the fluxes into any of the variables collectively equal the fluxes leaving this variable in magnitude.

Surprisingly, these fundamental features are not necessarily present in a template model. The variables (vertices) in a template model are usually physiological or biochemical *processes*, or conglomerations of such processes. In the case of the cholesterol template, for example, the variables are the mevalonate pathway, cholesterol storage, the dynamics of (cholesterol-containing) plasma lipids, and so on (**Fig 2**), which are all processes altering the concentration of free or bound cholesterol. Concomitantly, the directed edges in the template model do not represent material fluxes. Rather, they indicate modifying effects that may increase or decrease the activity of another template variable. These effects either consist of handing molecules like cholesterol from one subsystem to another or they represent indirect modulations. Importantly, there is no material flow between template variables in a strict sense, because "Biosynthesis" does not become "Plasma Dynamics," in analogy to "Lanosterol" becoming "Cholesterol" in the anchor model. Instead, the variable "Diet" increases the amounts of cholesterol in the variables "Plasma Dynamics" and "Storage," and the variable "Biosynthesis" indirectly affects an increase in the input to the template variable "Sex Hormone Production." Furthermore, material may be generated or disappear within boxes. For instance, cholesterol disappears within the box "Sex Hormone Production," because it is converted into testosterone, estradiol, and other steroids. In the template box "Biosynthesis", cholesterol is apparently created out of nothing. Thus, a more accurate, although slightly awkward depiction of the cholesterol template is shown in **Fig 6**. It is of note that this representation actually runs counter to the standards of typical biological diagrams [18]. As an example, consider the signal emitted by a box like "Tissue Usage" on

"Storage" within this diagram: It signifies that increased use of cholesterol in the peripheral tissues indirectly triggers a release of cholesterol from storage in the liver.

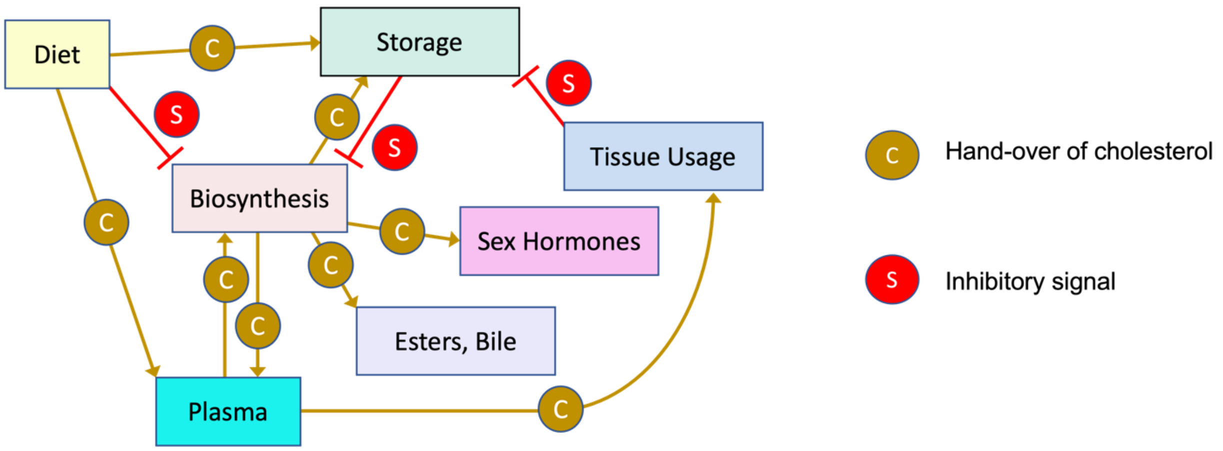

Fig 6: Cholesterol template model as dual graph. New representation of the cholesterol template model, where variables are in truth processes and edges either represent the moving of cholesterol C among processes or indicate inhibitory signals S Note that the arrows defy the standards of typical proper model diagrams [18].

The stark difference in model structures has implications for the stacking of sub-models during the zooming between templates and anchors. Specifically, it is possible that "anchors" associated with a template model in fact have the structure of lower-level templates. As an example, suppose a high-level template represents some aspect of cellular physiology. The anchors then could represent mitochondrial energy handling, protein synthesis, generation of reactive oxygen species, and a feature like cell contraction. These anchors clearly have the structure of template models, as they represent systems of processes, rather than processes whose variables are

biological entities like metabolites. Thus, in typical T&A models, the variables of a template models might be true anchor models, which are usually not further reducible to sub-models, or they are themselves lower-level template models with variables that may either be further templates or true anchors. For some psychological reasons, we tend to think downward from a template to anchors. Here, it might be more convenient to start with pure anchors at the lowest level of interest, which become the variables of templates at a higher level, which in turn are integrated in template models at even higher levels until one reaches an overarching umbrella template. As an example, different from our case study, consider again a typical physiologically based pharmacokinetic (PBPK) model (**Fig 1**). At first glance, the variables appear to be organs, but a slightly misleading moniker like "Liver" in truth means the concentration of a drug being processed by this organ at some point in time. The value of this variable is not only driven by the influx of the drug from the arterial blood and its efflux into the venous blood but also by the enzymatic, transport, and storage activities that alter (*e.g.*, bind, degrade or excrete) some of this drug within the organ. Without accounting for this enzymatic activity or excretion, there is no valid flux balance at a steady state. This situation is reconciled if one recognizes that the variable "Liver" represents a system of processes in which the overall influx is converted into the overall efflux. In other words, this situation of a PBPK model is typical for a template model, whose anchor "Liver" is a lower-level template that contains subsystems of biosynthesis, degradation, excretion, or storage. At the same time, each apparent flux in the PBPK model in truth represents the concentration of the drug being transported from one organ to another. As yet a different example, we recently proposed a template model for cystic fibrosis, where the variables at first glance are organs (*e.g.*, intestine), but in reality consist of processes such as digestion [52].

Taken together, anchors have the structure of typical biomathematical models. Their constituents are pools (vertices, *e.g.*, organic compounds) and arrows (edges; *e.g.*, enzymatic reactions, transport processes). By contrast, typical template models have the structure of dual systems: The vertices are processes, and the edges are entities like chemical compounds or signals. In the relationship between templates and anchors, the template variables become anchor models. These anchors may be true anchors or new, lower-level template models. A true anchor, like “Biosynthesis” in **Fig 2**, contains variables that are molecules (*e.g.*, lanosterol), which are handed off from one vertex to the next. Its processes are fluxes in the truest sense, as lanosterol is converted into—and thus becomes—cholesterol. However, if an anchor is a new template at a lower level, its variables are again processes or systems of processes. The edges of a template model, whether at the highest or a lower level, consist of signals or of entities (molecules) that are handed from one anchor to the next. Thus, the multiscale organization effort is complete once all anchor models are true anchors with variables that are entities rather than processes.

There are bound to be exceptions to this generalization of the T&A structure, but the feature of duality in typical T&A models highlights the fundamental difference between templates and anchors. It is unclear whether the recognition of this duality has practical ramifications beyond academic insights, but it does demonstrate that the two model types in T&A structures contain intrinsic differences. These differences offer guideposts for an effective hierarchical model design that spans different levels.

Discussion

The T&A modeling approach originally suggested over two decades ago was used for the purpose of inferring a set of common design concepts of posture and movement from different modes of locomotion among terrestrial species [36]. The authors pursued this goal by creating a model on a conceptual low-dimensional manifold describing the key aspects of locomotion. This invariant manifold was conceived as being embedded within a much-higher-dimensional space describing the various observed modes of locomotion, and all species-specific implementations were seen as intersecting in the subspace of the common design [36, 37, 53].

We propose in this article the T&A paradigm for a different purpose, namely for analyzing multiscale biomedical models in an effective manner. As demonstrated here, the approach provides a systematic and intuitively organized strategy for multi-level analysis of complex biological systems. The template model serves as a high-level, coarse-grained representation that focuses on the main physiological components of a system and entails a minimal set of variables and parameters. It acts as an overarching view of the entire system, thus providing an overall snapshot of the dynamic interactions among its core constituents. By contrast, the anchor models provide a more detailed and finely grained view of specific biological details and mechanisms within the system. They allow the user to zoom into individual variables within the template, thereby permitting an account of intricate aspects of the underlying processes. Together, the template and anchor models form a hierarchical structure, allowing for both broad overviews and in-depth analyses, thereby offering a versatile tool for multiscale modeling.

As an illustration of the implementation of the T&A model, we describe the impact of dioxin exposure on hepatic cholesterol levels. Diverse simulation scenarios can be performed, such as different dietary cholesterol intake regimens and the combined effect of dietary cholesterol

variation with dioxin exposure on the hepatic and plasma cholesterol levels [42]. In this example, the dose-response relationship between dioxin exposure and the corresponding hepatic cholesterol concentration is the main output that will subsequently inform the template model.

The T&A modeling approach carries distinct advantages in handling multiscale problems. In particular, the method enhances computational efficiency through a divide-and-conquer strategy and provides a flexible structure for model setup and the definition of variables across different levels. In contrast to other multiscale approaches, each anchor is separately analyzed, yielding a comprehensive record of essential input-output relationships that inform other anchors and calibrate the template model, while allowing for the interchangeability of anchor models without affecting the overall template structure.

Of course, the T&A approach is no panacea, and our exposure model is obviously simplified, like any other model. Even for the single anchor discussed here, the mevalonate pathway, much more sophistication could—and maybe should—be considered. For instance, it has been demonstrated that gene expression differs among hepatocytes depending on where they are located within the micro-lobules of the liver [39, 54, 55]. Also, we performed our analysis at the steady state of the pathway system, which may seem restrictive. However, it is expected that the does-response curve we determined with the anchor model is similarly applicable to non-steady-state situations and that pathway within the human body operates relatively close to the steady state. Despite these limitations, the proposed approach appears to be sufficient for revealing the essence of a complex phenomenon, as in our example the impact of dioxin on cholesterol biosynthesis. A different caveat is that even the best model is only as good as the supporting data, and the data need for a complete T&A analysis may greatly exceed reality. In our example, the amount of human data is scarce and our supplanting them with mouse data raises clear

uncertainties. At a theoretical level, the duality between templates and anchors will require a deeper investigation and an exploration of its speculated generality.

Overall, multiscale models are clearly important, and the T&A approach is intended to provide a novel, effective tool for complex modeling tasks as it provides a comprehensive and holistic perspective that accommodates a range of organizational scales. It is quite evident that an approach like T&A can readily be tailored toward many different research goals and applications, which could, for instance, support traditionally difficult extrapolations of *in vitro* to *in vivo* results [56] and the important translation of observations between different species [57]. For our example of TCDD exposure, this translation poses an interesting challenge, as some enzyme activities and the crucial TCDD-AhR mechanism are species-specific [58, 59]. More generically, the T&A approach has the potential of serving as an effective tool for advancing computer-supported personalized medicine [60, 61] and in virtual clinical trials [62, 63].

Acknowledgements

This work was supported in part by NIEHS HERCULES grant P30ES019776-05 (PI: Carmen Marsit) and NIEHS Superfund Research grant P42ES04911 (PI: Norbert Kaminski). The funding agency is not responsible for the content of this article.

References

1. Schnell, S., R. Grima, and P. Maini, *Multiscale Modeling in Biology.* American Scientist - AMER SCI, 2007. **95**.
2. Savageau, M.A., *The challenge of reconstruction.* New Biol, 1991. **3**(2): p. 101-2.
3. Savageau, M.A., *Reconstructionist molecular biology.* New Biol, 1991. **3**(2): p. 190-7.
4. Kreeger, P.K. and D.A. Lauffenburger, *Cancer systems biology: a network modeling perspective.* Carcinogenesis, 2009. **31**(1): p. 2-8.
5. Gay, L., A.M. Baker, and T.A. Graham, *Tumour Cell Heterogeneity.* F1000Res, 2016. **5**.
6. Dagogo-Jack, I. and A.T. Shaw, *Tumour heterogeneity and resistance to cancer therapies.* Nature Reviews Clinical Oncology, 2018. **15**(2): p. 81-94.
7. Shurin, M.R., *Cancer as an immune-mediated disease.* Immunotargets Ther, 2012. **1**: p. 1-6.
8. Goetz, L.H. and N.J. Schork, *Personalized medicine: motivation, challenges, and progress.* Fertil Steril, 2018. **109**(6): p. 952-963.
9. Berman, G.J., *Measuring behavior across scales.* BMC Biol, 2018. **16**(1): p. 23.
10. Anderson, A.R. and V. Quaranta, *Integrative mathematical oncology.* Nat Rev Cancer, 2008. **8**(3): p. 227-34.
11. Liu, L. and A. Bockmayr, *Formalizing Metabolic-Regulatory Networks by Hybrid Automata.* Acta Biotheor, 2020. **68**(1): p. 73-85.
12. Weinan, E. and B. Engquist, *Multiscale Modeling and Computation.* Notices of the American Mathematical Society, 2011. **50**.
13. Groen, D., et al., *Mastering the scales: a survey on the benefits of multiscale computing software.* Philosophical Transactions of the Royal Society A: Mathematical, Physical and Engineering Sciences, 2019. **377**(2142): p. 20180147.
14. Millar-Wilson, A., et al., *Multiscale modeling in the framework of biological systems and its potential for spaceflight biology studies.* iScience, 2022. **25**(11): p. 105421.
15. Peng, G.C.Y., et al., *Multiscale Modeling Meets Machine Learning: What Can We Learn?* Archives of Computational Methods in Engineering, 2021. **28**(3): p. 1017-1037.
16. Ingólfsson, H.I., et al., *Machine Learning-Driven Multiscale Modeling: Bridging the Scales with a Next-Generation Simulation Infrastructure.* J Chem Theory Comput, 2023. **19**(9): p. 2658-2675.
17. Voit, E.O., *The Inner Workings of Life: Vignettes in Systems Biology*. 2016: Cambridge University Press.
18. Voit, E., *A First Course in Systems Biology (2nd ed.). Garland Science*. 2017.
19. Liu, R., et al., *A Physiologically Based Pharmacokinetic (PBPK) Modeling Framework for Mixtures of Dioxin-like Compounds.* Toxics, 2022. **10**(11).
20. Bonabeau, E., *Agent-based modeling: Methods and techniques for simulating human systems.* Proceedings of the National Academy of Sciences, 2002. **99**(suppl_3): p. 7280-7287.
21. Wilensky, U. and W. Rand, *An Introduction to Agent-Based Modeling Modeling Natural, Social, and Engineered Complex Systems with NetLogo*. 2015: The MIT Press.
22. Monti, C., et al., *On learning agent-based models from data.* Scientific Reports, 2023. **13**(1): p. 9268.
23. Versypt, A.N.F., *Multiscale modeling in disease.* Curr Opin Syst Biol, 2021. **27**.
24. Glen, C.M., M.L. Kemp, and E.O. Voit, *Agent-based modeling of morphogenetic systems: Advantages and challenges.* PLOS Computational Biology, 2019. **15**(3): p. e1006577.
25. Kasereka, S., N. Kasoro, and P. Chokki, *A hybrid model for modeling the spread of epidemics: Theory and simulation*. 2014.

26. Bardini, R., et al., *Multi-level and hybrid modelling approaches for systems biology.* Comput Struct Biotechnol J, 2017. **15**: p. 396-402.
27. Garbey, M., S. Casarin, and S.A. Berceli, *A versatile hybrid agent-based, particle and partial differential equations method to analyze vascular adaptation.* Biomechanics and Modeling in Mechanobiology, 2019. **18**(1): p. 29-44.
28. Cruz, D.A. and M.L. Kemp, *Hybrid computational modeling methods for systems biology.* Progress in Biomedical Engineering, 2022. **4**(1): p. 012002.
29. Cassidy, R., et al., *Mathematical modelling for health systems research: a systematic review of system dynamics and agent-based models.* BMC Health Services Research, 2019. **19**(1): p. 845.
30. Lättilä, L., P. Hilletofth, and B. Lin, *Hybrid simulation models – When, Why, How?* Expert Systems with Applications, 2010. **37**(12): p. 7969-7975.
31. Liao, J.C. and E.N. Lightfoot, Jr., *Lumping analysis of biochemical reaction systems with time scale separation.* Biotechnol Bioeng, 1988. **31**(8): p. 869-79.
32. Savageau, M.A., *Growth of complex systems can be related to the properties of their underlying determinants.* Proceedings of the National Academy of Sciences, 1979. **76**(11): p. 5413-5417.
33. Gunawardena, J., *Time-scale separation--Michaelis and Menten's old idea, still bearing fruit.* Febs j, 2014. **281**(2): p. 473-88.
34. Ferreira, A.E.N., A.M. Ponces Freire, and E.O. Voit, *A quantitative model of the generation of N(epsilon)-(carboxymethyl)lysine in the Maillard reaction between collagen and glucose.* Biochem J, 2003. **376**(Pt 1): p. 109-21.
35. Collier, M.L., et al., *Calcium-induced calcium release in smooth muscle: loose coupling between the action potential and calcium release.* J Gen Physiol, 2000. **115**(5): p. 653-62.
36. Full, R.J. and D.E. Koditschek, *Templates and anchors: neuromechanical hypotheses of legged locomotion on land.* J Exp Biol, 1999. **202**(Pt 23): p. 3325-32.
37. Kvalheim, M., and Revzen, S, *Templates and Anchors; in M.A*. Bioinspired Legged Locomotion. Models, Concepts, Control and Applications, ed. S.a.A. Seyfarth. 2017, Oxford, UK.
38. Angrish, M.M., C.Y. Dominici, and T.R. Zacharewski, *TCDD-elicited effects on liver, serum, and adipose lipid composition in C57BL/6 mice.* Toxicol Sci, 2013. **131**(1): p. 108-15.
39. Fader, K.A., et al., *2,3,7,8-Tetrachlorodibenzo-p-Dioxin Alters Lipid Metabolism and Depletes Immune Cell Populations in the Jejunum of C57BL/6 Mice.* Toxicol Sci, 2015. **148**(2): p. 567-80.
40. Institute of Medicine Committee to Review the Evidence Regarding the Link Between Exposure to Agent, O.D., in *Veterans and Agent Orange: Herbicide/Dioxin Exposure and Type 2 Diabetes*. 2000, National Academies Press (US) Copyright 2000 by the National Academy of Sciences. All rights reserved.: Washington (DC).
41. Tuomisto, J.T., et al., *Soft-tissue sarcoma and dioxin: A case-control study.* Int J Cancer, 2004. **108**(6): p. 893-900.
42. Kumbale, C.M., Q. Zhang, and E.O. Voit, *Hepatic cholesterol biosynthesis and dioxin-induced dysregulation: A multiscale computational approach.* Food and Chemical Toxicology, 2023: p. 114086.
43. Greene, J.F., S. Hays, and D. Paustenbach, *Basis for a proposed reference dose (RfD) for dioxin of 1-10 pg/kg-day: a weight of evidence evaluation of the human and animal studies.* J Toxicol Environ Health B Crit Rev, 2003. **6**(2): p. 115-59.
44. Voit, E.O., *The best models of metabolism.* Wiley Interdiscip Rev Syst Biol Med, 2017. **9**(6).
45. Voit, E.O., *Computational Analysis of Biochemical Systems: A Practical Guide for Biochemists and Molecular Biologists*. 2000: Cambridge University Press.
46. Voit, E.O., *Biochemical Systems Theory: A Review.* ISRN Biomathematics, 2013. **2013**: p. 897658.
47. Malhotra, P., et al., *Disturbances in Cholesterol Homeostasis and Non-alcoholic Fatty Liver Diseases.* Front Med (Lausanne), 2020. **7**: p. 467.

48. Dassios, I. and F. Milano, *Singular dual systems of fractional-order differential equations.* Mathematical Methods in the Applied Sciences, 2021.
49. Rudolph, J., *Duality in time-varying linear systems: A module theoretic approach.* Linear Algebra and its Applications, 1996. **245**: p. 83-106.
50. Bruce, J.W. and F. Tari, *Duality and implicit differential equations.* Nonlinearity, 2000. **13**(3): p. 791.
51. Marin-Sanguino, A., E.R. Mendoza, and E.O. Voit, *Flux duality in nonlinear GMA systems: implications for metabolic engineering.* J Biotechnol, 2010. **149**(3): p. 166-72.
52. Olivença, D.V., et al., *Mathematical models of cystic fibrosis as a systemic disease.* WIREs Mech Dis, 2023: p. e1625.
53. Holmes, P., et al., *The Dynamics of Legged Locomotion: Models, Analyses, and Challenges.* SIAM Review, 2006. **48**(2): p. 207-304.
54. Nault, R., et al., *Dose-Dependent Metabolic Reprogramming and Differential Gene Expression in TCDD-Elicited Hepatic Fibrosis.* Toxicol Sci, 2016. **154**(2): p. 253-266.
55. Nault, R., et al., *Lipidomic Evaluation of Aryl Hydrocarbon Receptor-Mediated Hepatic Steatosis in Male and Female Mice Elicited by 2,3,7,8-Tetrachlorodibenzo-p-dioxin.* Chem Res Toxicol, 2017. **30**(4): p. 1060-1075.
56. Zhang, Q., et al., *Bridging the Data Gap From in vitro Toxicity Testing to Chemical Safety Assessment Through Computational Modeling.* Frontiers in Public Health, 2018. **6**.
57. Brubaker, D.K., et al., *An interspecies translation model implicates integrin signaling in infliximab-resistant inflammatory bowel disease.* Sci Signal, 2020. **13**(643).
58. Schulz, T.G., et al., *Inducibility of cytochromes P-450 by dioxin in liver and extrahepatic tissues of the marmoset monkey (Callithrix jacchus).* Biochimica et Biophysica Acta (BBA) - Protein Structure and Molecular Enzymology, 1996. **1298**(1): p. 131-140.
59. Fader, K.A. and T.R. Zacharewski, *Beyond the Aryl Hydrocarbon Receptor: Pathway Interactions in the Hepatotoxicity of 2,3,7,8-Tetrachlorodibenzo-p-dioxin and Related Compounds.* Curr Opin Toxicol, 2017. **2**: p. 36-41.
60. Voit, E.O. and K.L. Brigham, *The Role of Systems Biology in Predictive Health and Personalized Medicine.* The Open Pathology Journal,, 2008. **2**: p. 68-70.
61. Davis, J.D., et al., *Dynamical systems approaches to personalized medicine.* Curr Opin Biotechnol, 2019. **58**: p. 168-174.
62. Brown, D., et al., *Trauma in silico: Individual-specific mathematical models and virtual clinical populations.* Science Translational Medicine, 2015. **7**(285): p. 285ra61-285ra61.
63. Craig, M., et al., *A practical guide for the generation of model-based virtual clinical trials.* Frontiers in Systems Biology, 2023. **3**.
