# Supplementary material for: Analysis of systemic effects of dioxin on human health through template-and-anchor modeling
Source: PLoS Comput Biol. 2025 Mar 27;21(3):e1012840. doi: 10.1371/journal.pcbi.1012840 (PMC12005559; doi:10.1371/journal.pcbi.1012840)
Supplement: Attachment — Submitted filename: PCOMPBIOL-D-24-00128_R2_reviewer_R2.pdf [file pcbi.1012840.s005.pdf]

PLOS Computational Biology

Analysis of Systemic Effects of Dioxin on Human Health through Template-and-Anchor Modeling

--Manuscript Draft--

**Manuscript Number:**	PCOMPBIOL-D-24-00128R2
**Full Title:**	Analysis of Systemic Effects of Dioxin on Human Health through Template-and-Anchor Modeling
**Short Title:**	Effects of Dioxin on Human Health Analyzed through Template-and-Anchor Modeling
**Article Type:**	Research Article
**Keywords:**	Cholesterol; Dioxin; Dose-Response-Curve; Lipoproteins; Multiscale Modeling; Steroidogenesis
**Abstract:**	Dioxins are persistent environmental pollutants known for their multiple health effects, from skin rashes to liver dysfunction, reproductive toxicity and cancer. While the hazards of dioxins have been well documented, the challenge of developing a comprehensive understanding of the overall health impact of dioxins remains. We propose to address this challenge with a new approach methodology (NAM) consisting of a novel adaptation of the Template-and-Anchor (T&A) modeling paradigm. Generically, the template model is defined as a high-level coarse-grained model capturing the main physiological processes of the system. The variables of this template model are anchor models, which represent component sub-systems in greater detail at lower biological levels. For the case of dioxin, we design the template to capture the systemic effects of dioxin on the body's handling of cholesterol. Two new anchor models within this template elucidate the effects of dioxin on cholesterol transport in the bloodstream and on sex hormone steroidogenesis and the menstrual cycle. A third anchor model, representing dioxin-mediated effects on cholesterol biosynthesis via the mevalonate pathway, had been developed previously [1]. The T&A modeling paradigm enables a holistic evaluation of the impact of toxicants, which in the future may be translated into a powerful tool for comprehensive computational health risk assessments, personalized medicine, and the development of virtual clinical trials.
**Additional Information:**	
**Question**	**Response**
**Government Employee**<br><br>Are you or any of the contributing authors an employee of the United States government?<br><br>Manuscripts authored by one or more US Government employees are not copyrighted, but are licensed under a CC0 Public Domain Dedication, which allows unlimited distribution and reuse of the article for any lawful purpose. This is a legal requirement for US Government employees.<br><br>This will be typeset if the manuscript is accepted for publication.	No - No authors are employees of the U.S. government.

Powered by Editorial Manager® and ProduXion Manager® from Aries Systems Corporation

**Financial Disclosure**<br><br>Enter a financial disclosure statement that describes the sources of funding for the work included in this submission. Review the submission guidelines for detailed requirements. View published research articles from *PLOS Computational Biology* for specific examples.<br><br>This statement is required for submission and **will appear in the published article** if the submission is accepted. Please make sure it is accurate.<br><br>**Funded studies**<br>Enter a statement with the following details:<br>• Initials of the authors who received each award<br>• Grant numbers awarded to each author<br>• The full name of each funder<br>• URL of each funder website<br>• Did the sponsors or funders play any role in the study design, data collection and analysis, decision to publish, or preparation of the manuscript?<br><br>Did you receive funding for this work?	Yes
Please add funding details.<br>as follow-up to "**Financial Disclosure**<br><br>Enter a financial disclosure statement that describes the sources of funding for the work included in this submission. Review the submission guidelines for detailed requirements. View published research articles from *PLOS Computational Biology* for specific examples.<br><br>This statement is required for submission and **will appear in the published article** if the submission is accepted. Please make sure it is accurate.	NIEHS HERCULES grant P30ES019776-05 (PI: Carmen Marsit)<br><br>NIEHS Superfund Research grant P42ES04911 (PI: Norbert Kaminski)

**Funded studies**<br>Enter a statement with the following details:<br>• Initials of the authors who received each award<br>• Grant numbers awarded to each author<br>• The full name of each funder<br>• URL of each funder website<br>• Did the sponsors or funders play any role in the study design, data collection and analysis, decision to publish, or preparation of the manuscript?<br><br>Did you receive funding for this work?"	
Please select the country of your main research funder (please select carefully as in some cases this is used in fee calculation).<br>as follow-up to "**Financial Disclosure**<br><br>Enter a financial disclosure statement that describes the sources of funding for the work included in this submission. Review the submission guidelines for detailed requirements. View published research articles from *PLOS Computational Biology* for specific examples.<br><br>This statement is required for submission and **will appear in the published article** if the submission is accepted. Please make sure it is accurate.<br><br>**Funded studies**<br>Enter a statement with the following details:<br>• Initials of the authors who received each award<br>• Grant numbers awarded to each author<br>• The full name of each funder<br>• URL of each funder website<br>• Did the sponsors or funders play any role in the study design, data collection and analysis, decision to publish, or preparation of the manuscript?<br><br>Did you receive funding for this work?"	UNITED STATES - US
**Competing Interests**	The authors declare no competing interests.

On behalf of all authors, disclose any competing interests that could be perceived to bias this work.<br><br>This statement will be typeset if the manuscript is accepted for publication.<br><br>Review the instructions link below and PLOS Computational Biology's competing interests policy to determine what information must be disclosed at submission.	
**Data and Code Availability**<br><br>From the time of publication, Authors are required to make fully available and without restriction all data and computational code underlying their findings. Please see our PLOS Data Policy page for detailed policy information, and our Code Sharing page for specific information on code sharing.<br><br>A **Data Availability Statement**, detailing where the data (and code, if applicable) can be accessed, is required at first submission. Insert your Data Availability Statement in the box below. The statement you provide **will be published in the article**, if accepted.<br><br>Please see the Data Reporting section of our submission guidelines for instructions on what you need to include in your Data Availability Statement.<br><br>If the data and code are all contained in your submission files, please state: *All relevant data are within the manuscript and its Supporting Information files.*<br><br>**PLOS allows rare exemptions to address legal and ethical concerns.** If you have legal or ethical restrictions, please detail these in your Data Availability Statement below for the Journal team to consider.	No data used.<br>Code is available on GitHub, as stated in the articles.

Powered by Editorial Manager® and ProduXion Manager® from Aries Systems Corporation

Dear Professor Mendes:

In this newest revision, we have followed your suggestion and merged the two papers into one. In the process, we streamlined the paper in order to keep the length reasonable. The content of the previous responses to reviewers is still pertinent. We have left in the websystem the previous manuscript with very extensive tracker changes. However, it became infeasible to add to these changes, as there were so many alterations with our intermediate sequence of two papers and the consolidation.

I hope you and the reviewers will be pleased with the result.

Previous Rebuttal

Thank you for the constructive reviewer comments and the opportunity to revise our work. While we are responding to the reviewer comments one-by-one below, the major aspect of the revision is that we have followed the first reviewer's suggestion of creating an accompanying article that contains a comprehensive illustration of how a template-and-anchor (T&A) model is to be set up and analyzed. Importantly, it makes it clearer how information flows between the two levels. We are confident that this substantial change is a true improvement and hope that you and the reviewers will agree with our decision. In addition, we have made substantial editions to the present (first) paper, taking into consideration the constructive comments made by the reviewers.

Below, please find itemized responses to all specific comments and suggestions by the reviewers.

In addition to the two manuscript, we are submitting the original manuscript with Tracker changes for the reviewers' perusal.

Responses to the Reviewers' Comments

Reviewer #1

The manuscript presents a complex set of ideas with potential, but several questions arose as I finished reading it, mainly regarding clarity in exposition and organization.

Response: Thank you for the encouragement. In this revision, we have tried to reduce ambiguities in our description of the method.

While the paper introduces the Template and Anchor (T&A) approach for multiscale modeling, the conceptual framework of T&A modeling remains somewhat unclear. The distinction between template and anchor models seems inconsistently articulated throughout the paper, potentially causing confusion for both myself and readers

unfamiliar with the concept. A clearer definition and differentiation between template and anchor models in the introduction section could help, but may not sufficiently elucidate the adaptation from biomechanics to biomolecular levels.

Response: Thank you for the suggestions. We have devoted substantial effort to clarifying concepts and methodological details throughout the manuscript. As part of this rewriting, we have amended the introduction by adding clarifying verbiage. Importantly, following your suggestion, we have created an entirely new companion article with a detailed example of a T&A analysis of dioxin-mediated effects on cholesterol dynamics and steroidogenesis. This example discusses all steps required for the set-up of the template model and of various anchor models and then illustrates how information is transmitted from the anchors to the template model. We refer to this example in the introduction and in other locations of the first article.

The transition from a mechanistic-like model to a graph-theoretical model needs clearer explanation.

Response: There is not really a transition, as the mechanistic model is not directly affected by the graph-theoretical model or *vice versa*. Instead, the reference to graph theory is purely an analogy, which may help some readers see the intriguing differences between template and anchor models. We have attempted to make this analogy clearer.

Duality of graphs is evoked only to demonstrate that the template model and the anchor models have a different character. In particular, a conventional model, including a typical anchor model, contains variables that represent molecules or other physical quantities. By contrast, we argue, that a template model contains variables that do not represent molecules or physical quantities but processes. This insight has consequences for setting up the different models and their constituents. Having said that, we propose that anchor models may serve as template models at lower levels. In this case, the character of the model changes. As an example, “cholesterol storage in the liver” is currently an anchor model that contains as main variable the currently stored amount of cholesterol. However, if this anchor model is to become a template for an underlying model of finer granularity, the variables become the molecular and physiological processes of storing and releasing cholesterol in response to demands. The result could be a template model, with its own anchors, for a lower organizational level.

In addition to amending the text, we refer to the companion paper, which describes the design and analysis of both model types and the conceptual interactions between them. We also provide additional references to papers on graph duality that are considered the first to discuss duality of graphs. Indeed, this topic has been discussed in the literature for a long time, using different terms, such as “line graphs,” “edge graphs” and “edge-to-vertex duals,” among others. Line graphs were arguably first mentioned by Hassler Whitney in 1931/1932. A good review is the 1972 book by Harary and, in particular, the chapter on line graphs within this book. In our case, the specific targets are actually directed graphs (“digraphs”) and the corresponding “line digraphs,” which were discussed in Harary and Norman (1960) and Hemminger and Beineke (1978).

Whitney, H. (1931) Non-separable and planar graphs. *PNAS* **17**, 125-127.
Whitney, H. (1932) Congruent Graphs and the Connectivity of Graphs. *American Journal of Mathematics* **54 (1)**, 150-168.
Harary, F. (1972) *Graph Theory*, Massachusetts: Addison-Wesley.
Harary, F., Norman, R.Z. (1960) Some properties of line digraphs. *Rend. Circ. Mat. Palermo* **9**, 161–168. https://doi.org/10.1007/BF02854581
Hemminger, R. L.; Beineke, L. W. (1978), "Line graphs and line digraphs", in Beineke, L. W.; Wilson, R. J., *Selected Topics in Graph Theory,* Academic Press Inc., pp. 271–305.

The current structure of the explanation gives the impression of a two-level modeling approach, rather than a multilevel one. Did I misunderstand this? For instance, how would one use the methodology to progress from molecular networks to tissues, organisms, and populations/epidemiological groups? How would more than two levels be addressed?

Response: It is true that the example we are analyzing primarily spans two levels. However, as we point out in the theoretical discussion, it is possible that an anchor model, *mutatis mutandis*, might in fact serve as the template model for lower-level anchors. By the same token, a template could become an anchor within a higher-level template model. In our example of the effect of dioxin on cholesterol handing, one could imagine that our template might serve as an anchor in a higher-level model that investigates the effects of dioxin on the entire organism and contains additional physiological systems, such as the skin, or assesses dioxin-related carcinogenesis. Clearly, these effects are not independent from cholesterol handling, which we discuss here, and our current template could serve as an anchor within an overall model of dioxin effects in parallel to other anchors.

At this point, our example in the companion article discusses the progression from molecular systems, such as metabolic pathways, to tissues and physiological systems, such as the liver and lipoprotein dynamics in the plasma. It also touches on reproductive dysfunction caused by dioxin, which clearly is a higher-level issue.

Nonetheless, the reviewer’s point is well taken. As detailed in the response to the previous comment, our current template could serve as an anchor in a higher-level template that might not only look at dioxin-induced cholesterol dysfunction but also at effects on other aspects of the organism, such as tumor formation. In terms of population-based phenomena, we mention the use of epidemiological data in the anchor of steroidogenesis. As a more generic example, one could imagine a template describing the spread of an infection (with a structure like a sophisticated, detailed SIRS model, maybe for an age-stratified population). The anchors could be individuals who interact and thereby spread the disease. Each individual would be characterized by her/his social interactions with others and the potential of being infected. For each individual, one might model the inhalation processes and the immune system with individualized properties, which would determine the effectiveness of being infected, fighting the infection through a strong immune response and the concomitant transmission potential of the disease. Under the same template, it could also be possible to create anchor models of bacteria or viruses causing the disease. Clearly, there are interactions between the pathogens and the individuals’ immune systems and,

thus among the anchors at this level. At a more granular level, these anchors could be templates for underlying physiological or molecular systems.

We are sorry if all this sounds a bit vague, but the use of Full and Koditchek's original T&A approach proposed here is new and so far untested in terms of possibilities and limitations.

While the paper discusses practical applications of the T&A approach, such as personalized medicine and translational research, it lacks specificity in describing how the approach can be applied in real-world scenarios. While the example presented throughout the paper is helpful, it is insufficient to fully illustrate the complex concepts. There are two way I can see to improve this. One is by creating a set of sections or a detailed methodology (or a second accompanying manuscript) where the concepts are properly formalized. The alternative, is providing more examples with similar depth to the one you use to enhance readers' understanding of the underlying concepts. Highlight specific challenges addressed and insights gained through T&A modeling in diverse contexts.

Response: Thank you for this suggestion, which we have wholeheartedly accepted. The new accompanying article presents a very detailed example that illustrates model design and analysis. We have shown elsewhere [Voit and Brigham, 2008; Davis *et al.*, 2019] how population-based models may be personalized, and the T&A approach could be personalized in a conceptually straightforward manner (even though substantial information would be needed for such an implementation). Nonetheless, the structure of the T&A approach is no hindrance toward personalization. As an indication in this direction, we previously studied interactions between dioxin exposure and dietary cholesterol uptake [Kumbale *et al.*, 2023].

We also mention that the well-known physiologically-based pharmacokinetic (PBPK) models can be seen as simple T&A models.

We have added a section to the discussion on how the T&A framework can be effectively utilized in personalized medicine and for drug discovery.

Voit, E. O. and K. L. Brigham: The role of systems biology in predictive health and personalized medicine. *The Open Path. J.* **2**, 68-70, 2008.

Davis, J.D., Kumbale, C.M., Zhang, Q. and E.O. Voit: Dynamical systems approaches to personalized medicine. *Curr. Opin. Biotechn.* **58**, 168-174, 2019.

Kumbale, C.M., Q. Zhang, and E.O. Voit: Hepatic cholesterol biosynthesis and dioxin-induced dysregulation: A multiscale computational approach. *Food Chem. Toxicol.* **181**:114086, 2023. doi: 10.1016/j.fct.2023.114086.

Additionally, while the paper acknowledges potential limitations and challenges associated with T&A modeling, the discussion remains relatively brief and lacks depth. Expanding the discussion section to include a more comprehensive analysis of the

limitations and challenges of T&A modeling would be beneficial. This could be done by including additional examples that are unrelated to the system that is used throughout the paper.

More detailed sections regarding the limitations for each of our developed anchor models and the overall template model have been added to the discussion of the present paper and in the companion paper.

The paper emphasizes the importance of empirical validation for T&A models but falls short of providing concrete guidance on validation methodologies and best practices. Utilizing more than one example to illustrate the framework and validate its models may facilitate this. The paper could also benefit from a more comprehensive analysis of potential avenues for advancing T&A modeling in biomedical research.

Model validation is always a challenge. Here, the anchors and the template are well structured and thus provides avenues toward individual and overall validation. In terms of anchors, validation and best practices following traditional guidelines that have been governing mathematical modeling for a long time. For instance, the model should be assured to be stable and sufficiently insensitive at the nominal operating point to withstand moderate external perturbations. Also, it is in principle possible to measure details, *e.g.*, for the cholesterol biosynthesis model, with experimental means. Given the paucity of experimental and clinical data, much of the available information was used to implement the model, but other details could be measured and compared with model results. In terms of the template model, one could pursue measurements, for instance, targeting the combination of dioxin exposure and different amounts of dietary cholesterol. Similarly, model results regarding steroidogenesis could be validated in principle.

There are some additional comments and typo that you can find in the attached pdf.

Response: Thank you for your careful reading and suggestions. We have taken care of all issues mentioned.

Reviewer #2

The manuscript revisits the Template-and-Anchor (T&A) modeling framework, originally proposed for design concepts from various manifestations of biological locomotion. The manuscript emphasizes the T&A framework's utility in simplifying complex multiscale biomedical models by separating them into high-level template models and more detailed anchor models. The template model describes overarching bioprocess components with minimal variables, while anchor models delve into specific biological details of subsystems. This separation enhances computational efficiency, provides a structured approach to model development, and maintains flexibility by allowing anchor

models to be independently updated or replaced. The manuscript illustrates this framework's application in the modeling of dioxin exposure and its effects on cholesterol dynamics, showcasing its potential.

The manuscript's exploration of the Template-and-Anchor (T&A) framework, particularly its application to biological systems modeling, is commendable. The presentation of the T&A framework is insightful, but its application to dioxin exposure needs clarifications (see Major Comments). I believe this work will likely be well-received by practitioners of biosystems modeling.

Response: Thank you for these encouraging comments.

Major comments:

1. The manuscript provides a general description of the Template-and-Anchor (T&A) framework. Unfortunately, it lacks detailed implementation specifics, particularly in the dioxin exposure case study. Essential elements such as the interaction between template and anchor models, specific equations governing the dioxin model, and the nature of information exchanged between template and anchor are notably absent. This omission hampers the reader's ability to fully understand and appreciate the model's complexity and operational mechanisms. For example, it is not clear how one would get the simulation result in Fig. 4 since no information of the model that was solved was provided.

Response: We hope that the extensive rewriting of the paper and the addition of the companion paper, as recommended by the first reviewer, offer sufficient methodological detail to support our claims. In particular, we have put much more emphasis outlining how the anchors and templates “interact” with each other. Also, we are now providing code on GitHub and in the Supplements of the companion paper that shows pertinent details.

2. Despite the mention of future code availability on GitHub, the current lack of access to the model's code restricts the ability to replicate the study and/or delve into the technical intricacies of the framework. The absence of code, coupled with the lack of mathematical detail and algorithmic descriptions in the manuscript, significantly detracts from the research's reproducibility, thus limiting its utility and impact within the scientific community.

Response: We are sorry that the previous version did not permit a reproduction of our results and are now providing code on GitHub and in the Supplements of the companion article.

3. The similarity between the Template-and-Anchor (T&A) framework and block diagrams in process control, particularly in how both describe input-output relationships through modular components, deserves a comparison. Like T&A template, block diagrams use blocks to represent the relationships between variables, where such relationships are modeled using transfer functions. The anchors could be seen as complex versions of block diagram transfer functions. A more explicit comparison highlighting the T&A framework's unique contributions and advantages in modeling biological systems, beyond what traditional block diagrams offer, would greatly enhance the manuscript's impact.

Response: Thank you for the insightful comment. In response, we have added new verbiage to the text.

In essentially all our modeling efforts, the first step is the construction of a block diagram or, as we often call it, a box-and-arrow diagram [Voit, 2000, 2017]. We consider this structuring and visualization of a biomedical problem as absolutely essential because, in a way, it replaces reality during the mathematical modeling and analysis process; for a somewhat philosophical account of this facet, please see [Voit, 2019].

While diagrams are crucial, they only go so far, because it is necessary to “bring them to life” by defining what exactly the boxes and the arrows mean. In the case of T&A models, this definition is very different, as the boxes (variables of the template model) turn out to be entire systems (anchors) at a different level of granularity. Thus, we most certainly agree that the first step is a block (box-and-arrow) diagram, but that the proposed approach deals with boxes that are more complex than in typical control theory. We have added verbiage to the text debating these issues.

Voit, E.O.: *Computational Analysis of Biochemical Systems. A Practical Guide for Biochemists and Molecular Biologists*, xii + 530 pp., Cambridge University Press, Cambridge, U.K., 2000.
Voit, E.O.: *A First Course in Systems Biology*. Second Edition, Garland Science, New York, NY, 2017.
Voit, E.O.: Perspective: Dimensions of the Scientific Method. *PLoS Comp. Biol.* **15(9)**: e1007279, 2019.

Have the authors made all data and (if applicable) computational code underlying the findings in their manuscript fully available?

Reviewer #1: **No:** Not yet. they say they will provide the code on GitHUb. I would not accept the paper before a link to the repository is provided.

Reviewer #2: **No:** The code generating the model simulations was not provided with the manuscript for review.

Response: We are now providing code on GitHub and in the Supplements of the companion paper.

Manuscript

Click here to access/download;Manuscript;New Paper - submitted.docx

Analysis of Systemic Effects of Dioxin on Human Health through Template-and-Anchor Modeling

Carla M. Kumbale*, Qiang Zhang,*** , and Eberhard O. Voit*,***,******

* Wallace H. Coulter Department of Biomedical Engineering, Georgia Institute of Technology and Emory University, Atlanta, GA 30332, USA.
** Gangarosa Department of Environmental Health, Rollins School of Public Health, Emory University, Atlanta, GA 30322, USA.
*** Co-corresponding author.
**** Department of Biological Sciences, University of Texas at Dallas, Richardson, TX 75080-3021, USA.

Email Addresses: Ckumbale3@gatech.edu; Qiang.zhang@emory.edu; Eberhard.voit@bme.gatech.edu

Keywords: Cholesterol; Dioxin; Dose-Response-Curve; Lipoproteins; Multiscale Modeling; Steroidogenesis

Abstract

Dioxins are persistent environmental pollutants known for their multiple health effects, from skin rashes to liver dysfunction, reproductive toxicity and cancer. While the hazards of dioxins have been well documented, the challenge of developing a comprehensive understanding of the overall health impact of dioxins remains. We propose to address this challenge with a new approach methodology (NAM) consisting of a novel adaptation of the Template-and-Anchor (T&A) modeling paradigm. Generically, the template model is defined as a high-level coarse-grained model capturing the main physiological processes of the system. The variables of this template model are anchor models, which represent component sub-systems in greater detail at lower biological levels. For the case of dioxin, we design the template to capture the systemic effects of dioxin on the body's handling of cholesterol. Two new anchor models within this template elucidate the effects of dioxin on cholesterol transport in the bloodstream and on sex hormone steroidogenesis and the menstrual cycle. A third anchor model, representing dioxin-mediated effects on cholesterol biosynthesis via the mevalonate pathway, had been developed previously [1]. The T&A modeling paradigm enables a holistic evaluation of the impact of toxicants, which in the future may be translated into a powerful tool for comprehensive computational health risk assessments, personalized medicine, and the development of virtual clinical trials.

Author Summary

Dioxin is a ubiquitous, persistent pollutant that can have a range of health effects from skin rashes to reproductive problems, liver toxicity and cancer. To characterize and understand pressing health risk problems like those caused by dioxin, the field of chemical toxicity testing has been encouraging the search for "new approach methodologies" (NAMs) that reduce the use of animal experiments. Here, we propose a computational NAM to assess how the diverse effects of dioxin on cholesterol handling in different organs can be quantified in an organism-wide manner. To this end, we use a novel adaptation of the Template-and-Anchor (T&A) modeling paradigm. The template captures the systemic effects of dioxin on the body's handling of cholesterol, while three anchors explore the impact of dioxin on cholesterol biosynthesis, cholesterol transport and metabolism in the bloodstream, and on female sex hormone synthesis. The T&A modeling strategy permits an overall evaluation of the impact of dioxin, which in the future may be translated into a potent tool for computational health risk assessments, personalized medicine, and the development of virtual clinical trials.

1. Introduction

Dioxins are persistent organic pollutants (POPs) that are exceptionally stable and therefore remain in the environment for a long time. In the human body, they often exhibit decade-long half-lives and therefore tend to accumulate in various tissues throughout life. Considering that the World Health Organization (WHO) reported worldwide exposure, dioxins present a potentially severe, global problem [2]. The most toxic among numerous congeners of dioxins is 2,3,7,8-tetrachlorodibenzo-p-dioxin (TCDD), where moderate to severe exposure is known to cause adverse health effects from skin disorders and hormonal disruption to reproductive and developmental dysfunction, liver damage, and cancer [3]. In the following, we will use the more colloquial term “dioxin” as referring primarily to “TCDD.”

At the molecular level, dioxin alters the expression of a wide range of genes, including prominently those involved in cholesterol biosynthesis, lipoprotein transport, and steroidogenesis through the estradiol biosynthesis pathway. Many of these alterations are mediated by the aryl hydrocarbon receptor (AhR) [4], which is generally recognized as a sensor for xenobiotic molecules.

Although the hazards of dioxin are well documented in a rich literature, developing a comprehensive understanding of its systemic health impacts remains challenging. Given the numerous contributing factors and their complex interactions, it is appealing to attempt capturing the overall effects of dioxin with computational methods. This pursuit aligns with the new trend toward “New Approach Methodologies” (NAMs) within the toxicological community. These NAMs can be defined as any molecular, analytical or computational method “that when used alone, or in concert with others, enables improved chemical safety assessments through more protective and/or relevant models and, as a result, contributes to the replacement of animals” [5]. Of note here is that computational approaches are included in the envisioned repertoire of desirable NAMs [6].

While it is easy to fathom that computational methods are well suited to capture the many aspects of systemic toxicological assessments, the broad spectrum of effects and the multiscale nature of the impact of dioxin on human health poses a challenge for the development of any comprehensive computational models (*e.g.*, [7-11]). The primary issue is the significant increase in the number of variables, processes, and parameters that must be carried across various temporal and spatial scales. Additionally, defining the exact linkages among these scales poses a substantial

hurdle as direct experimental or clinical evidence is often scarce. The complexity of these tasks translates into formidable computational demands and poses difficulties for model validation and standardization. In particular, simulating the system dynamics across various scales tends to be computationally intensive, uncertainties are likely to be compounded, and validating multiscale models often necessitates unique criteria for each level, as well as the overall system. Finally, the lack of standardized methodologies across different domains complicates the analysis, comparison, and interpretation of multiscale systems.

In response to the quest for NAMs, and embracing the complexity posed by the multiscale nature of the problem, we analyze here the organism-wide effect of dioxin on cholesterol handling with a computational approach based on the concepts of Template-and-Anchor (T&A) models [12]. In contrast to the original usage of this paradigm, namely as a tool for identifying biological design principles [12, 13], we use the T&A approach simply as a means for organizing details, data, and other pertinent information into a manageable computational structure that is conceptually and practically tractable and flexible enough to facilitate substitutions of sub-models with alternative models in a simple and efficient manner.

The T&A approach uses two types of models that are tightly connected. The template serves as a high-level, coarse-grained model that focuses on the main physiological components of a system and contains only a modest number of variables and their interactions. In contrast, anchor models are more detailed and describe specific biological subsystems or processes in much finer granularity.

As with any mathematical or computational model, the T&A approach is most certainly no panacea; we describe some of the limitations in the discussion. Nonetheless, it allows us to dissect health risk assessments of dioxin into manageable components.

In previous work, we presented one anchor model capturing dioxin-mediated effects on cholesterol biosynthesis via the mevalonate pathway [1]. Here, we introduce the overall template model, two additional anchor models, as well as their relationships to each other. One anchor elucidates the effects of dioxin on cholesterol transport through the plasma, while the other demonstrates the impact of dioxin on ovarian steroidogenesis, which utilizes cholesterol as the source material, and its subsequent effects on the menstrual cycle. Once these anchor models have been analyzed, we demonstrate how the results from these analyses are used to inform the template

model. The template model itself is designed to capture the overall impact of dioxin on the body's cholesterol handling and subsequent ramifications for steroidogenesis in the ovary.

1.1. Biomedical Background

1.1.1. Global effects of dioxin on cholesterol handling

As documented in the Comparative Toxicogenomics Database (CTD) [14], dioxins, and especially TCDD, affect the expression of a multitude of genes. In many cases, the direct sequelae of these alterations are unknown, but there is substantial information regarding three target areas associated with cholesterol handling: cholesterol biosynthesis through the mevalonate pathway; lipoproteins and their role of cholesterol transporters; and the reproductive system, especially the effects of dioxin on ovarian hormones. These three target areas are of course connected, leading to a complex web of effects of dioxin, which is sketched below. Table 1 displays the expression of genes directly associated with dioxin and the organ systems in the anchors.

1.1.2. Effects of dioxin on cholesterol biosynthesis

Hepatic cholesterol biosynthesis was described in detail in [1]. At its core is the mevalonate pathway, which consists of a highly regulated multistep process (**Figure 1**). It encompasses a metabolic cascade that begins with the synthesis of HMG-CoA (3-hydroxy-3-methyl-glutaryl-coenzyme A) and progresses through several enzymatic steps, ultimately leading to the production of cholesterol. The mevalonate pathway is tightly controlled through negative feedback regulation exerted by the transcription factor SREBP (Sterol Regulatory Element-Binding Protein), which ensures cholesterol homeostasis. Additionally, the anchor model describing this pathway includes the conversion of cholesterol into bile acids, which constitute a significant route of cholesterol utilization, the storage of cholesterol in the liver as cholesterol esters, and the exchange of cholesterol between the liver and plasma. Dietary cholesterol intake also influences this pathway, reflecting the impact of external cholesterol sources on internal biosynthesis and overall cholesterol balance.

Table 1. Genes, enzymes, and proteins positively (↑) or negatively (↓) affected by dioxin in the anchor models, according to the Comparative Toxicogenomics Database [14].

Gene	Enzymes / Proteins	Impact of Dioxin on Expression	Organisms	Sources
HMGCS1	HMG- CoA synthase	↓	Homo sapiens<br>Mus musculus	Iqbal K (2021)
HMGCR	HMG- CoA Reductase	↓	Homo sapiens<br>Mus musculus	Boutros PC (2009)
FDPS	Farnesyl diphosphate synthase	↓	Homo sapiens<br>Mus musculus	Fracchiolla NS (2016)
SQS	Squalene-synthase	↓	Homo sapiens<br>Mus musculus	Hurst CH (1998)
LSS	Lanosterol- synthase	↓	Homo sapiens<br>Mus musculus	Labaronne (2017)
APOA1	Apolipoprotein A1	↑	Mus musculus	Angrish (2013)
LCAT	Lecithin–cholesterol acyltransferase	↑	Mus musculus	Angrish (2013)
LDLR	Low-density lipoprotein receptor	↑	Mus musculus	Nault R (2017)<br>Angrish (2013)
LPL	Cytochrome P450 family 7 subfamily A member 1	↑	Homo sapiens<br>Mus musculus	Fader KA (2015)<br>Nault R (2017)
CYP17A1	cytochrome P450 family 17 subfamily A member 1	↓	Mus musculus	Fader KA (2015)
HSD3B1	hydroxy-delta-5-steroid dehydrogenase, 3 beta- and steroid delta-isomerase 1	↓	Homo sapiens<br>Mus musculus	Saurat JH (2012)
CYP19A1	cytochrome P450 family 19 subfamily A member 1	↓	Homo sapiens<br>Mus musculus	Baldridge MG (2015)
HSD17B1	hydroxysteroid 17-beta dehydrogenase 1	↓	Mus musculus	Karman (2012)
CYP1B1	cytochrome P450 family 1 subfamily B member 1	↑	Homo sapiens<br>Mus musculus	Iqbal K (2021)
COMT	catechol-O-methyltransferase	↑	Mus musculus	Boutros PC (2009)
CYP11A1	cytochrome P450 family 11 subfamily A member 1	↑	Homo sapiens<br>Mus musculus	Baldridge MG (2015)

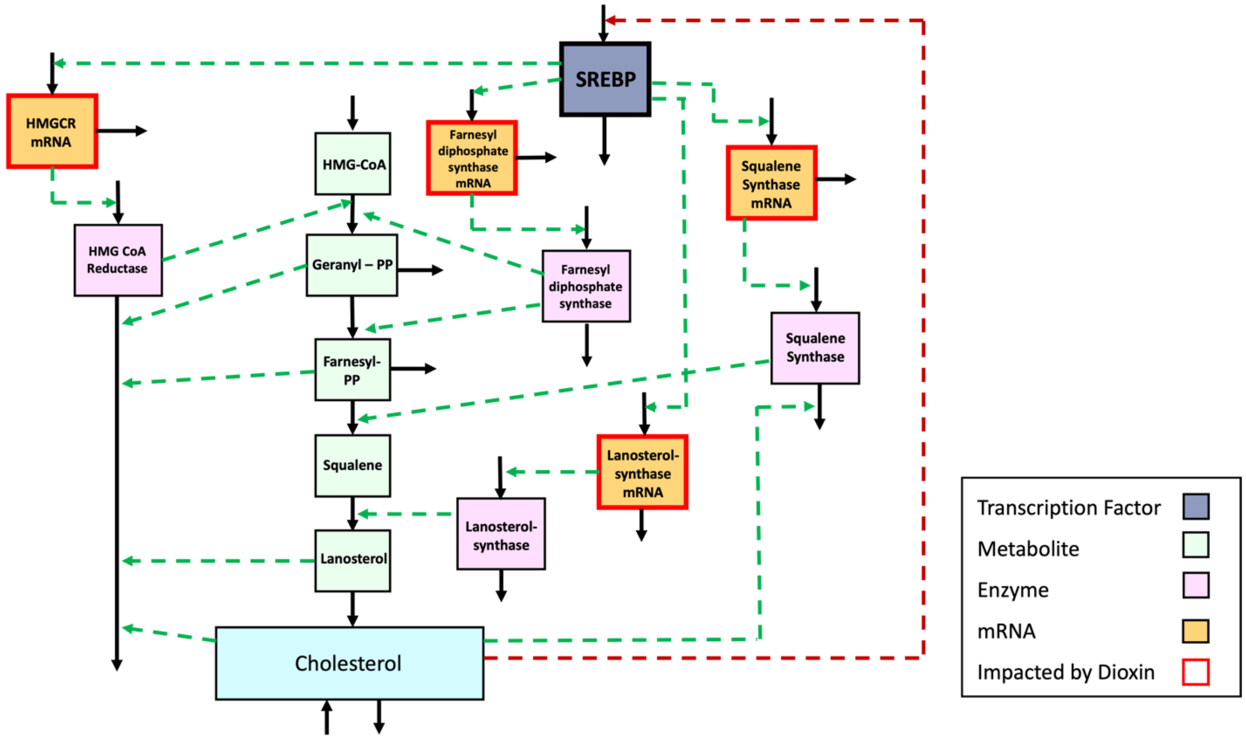

Figure 1. Hepatic cholesterol biosynthesis is the subject of one anchor model. The various components of the biosynthetic mevalonate pathway system are color-coded by type, as indicated. Metabolic reactions, transport steps, and the production of mRNAs and proteins are shown as solid lines, while regulatory signals, representing feedback inhibition (red) and modulation of enzyme activity [15], are given as dotted lines; mRNAs whose transcription is affected by dioxin, are indicated by boxes outlined in red. The key transcription factor SREBP binds to sterol regulatory elements (SREs) in the promoter regions of its target genes. It controls the activity of mevalonate pathway via these target genes, among which HMG CoA (3-hydroxy-3-methyl-glutaryl-coenzyme A) reductase is considered the rate-limiting step of the pathway, even though control of the pathway is distributed. Adapted from [1].

1.1.3. Effects of dioxin on lipoprotein dynamics

Lipoproteins serve as carriers of lipids in the bloodstream. They consist of a core of cholesterol esters and triglycerides surrounded by a shell of phospholipids, free cholesterol, and apolipoproteins [16]. Lipoproteins are typically classified into one of five groups: chylomicrons, very low-density lipoproteins (VLDL), VLDL remnants (IDL), low-density lipoproteins (LDL), and high-density lipoproteins (HDL) [16]. The literature on these lipoproteins is enormous.

A simplified depiction of lipoprotein metabolism and transport is shown in **Figure 2**. This diagram serves as the direct basis for constructing the anchor model (see Section 2.3).

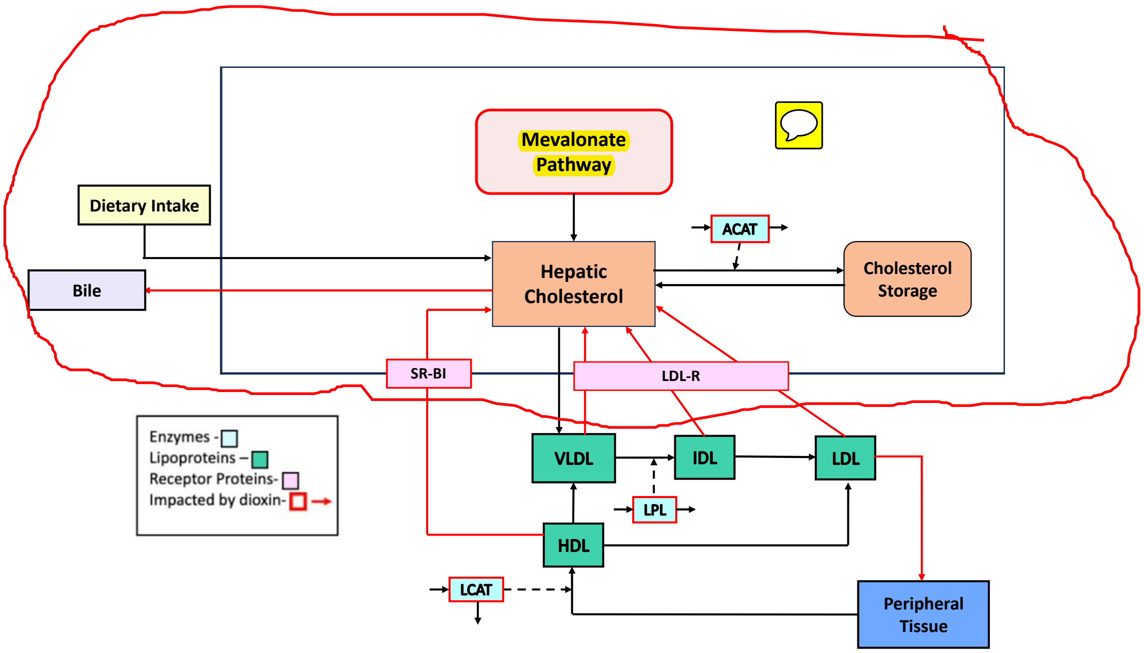

Figure 2. Overview of cholesterol metabolism and transport pathways. The diagram illustrates the processes of organism-wide cholesterol handling, and especially the involvement of the lipoproteins VLDL, IDL, LDL, and HDL, which are responsible for the transport of cholesterol between the liver and the peripheral tissues. The receptor proteins SR-BI and LDL-R, along with enzymes such as LPL and ACAT, are also depicted. The red boxes and arrows indicate the impact of dioxin on specific processes.

In the liver, cholesterol is synthesized *de novo* or taken up from the bloodstream via low-density lipoprotein receptors (LDL-R). Hepatic cholesterol can then be converted to cholesterol esters and stored, a process catalyzed by the enzyme acetyl-CoA acyltransferase (ACAT), or it can be incorporated into VLDL for secretion into the bloodstream. Once in the bloodstream, VLDL is hydrolyzed by lipoprotein lipase (LPL) leading to the formation of IDL. IDLs are either taken up by the liver or further catabolized to LDLs by an enzyme called hormone sensitive lipase (HSL). LDLs are then transported into the liver or peripheral cells where they are utilized for various cellular processes, including membrane synthesis and steroid hormone production [17]. LDL-R facilitates the uptake of VLDLs, IDLs, and LDLs into the liver [18]. Additionally, HDL acquires free cholesterol from the cell membranes of peripheral tissues, which allows it to be esterified by lecithin cholesterol acyltransferase (LCAT). It is believed that this esterification of cholesterol by LCAT promotes the net removal of cholesterol from peripheral tissue and incorporation into HDL [19]. HDLs then transport the excess cholesterol from peripheral tissues back to the liver for excretion, storage or recycling in a process called reverse cholesterol transport [20]. This process

is facilitated by scavenger receptor class B type 1 (SR-B1), which mediates selective uptake of cholesterol from HDL particles by hepatocytes [21].

According to CTD [14], dioxin strongly influences the lipoprotein transport pathway. Specifically, by interacting with AhR, dioxin can alter the expression of genes that are integral to the production, conversion, and uptake of lipoproteins. Genes that are pertinent for our model and impacted by dioxin in rodents and/or humans are presented in Table 1.

1.1.4. A brief overview of the female reproductive system

The female sex hormones 17β-estradiol (E2) and progesterone (P4) are synthesized in the ovaries. The steroidogenesis process occurs in the antral follicles during the follicular phase and additionally in the corpus luteum during the luteal phase. Antral follicles are composed of granulosa cells in the inner layers and theca cells in the outer layers. These two types of cells work together to produce sex steroid hormones according to the so-called two-cell-two-gonadotropin principle [22], according to which the theca cells utilize cholesterol to make progesterone and androgen, and androgen diffuses into the granulosa cells where it is converted to estrogen. The steroidogenesis in the corpus luteum works in a similar fashion, involving luteinized, differentiated theca and granulosa cells [23].

The initial rate-limiting step in the steroidogenesis pathway ~~(see **Figure 3** for a simplified representation)~~ is the transport of cholesterol from the mitochondrial outer membrane to the inner membrane, which is mediated by the steroidogenic acute regulatory protein (StAR). Subsequently, cholesterol is converted to pregnenolone (P5) by the cholesterol side-chain cleavage enzyme 20,22-desmolase, which is also called CYP11A1 and is located in the inner mitochondrial membrane [24-26]. P5 then moves out of the mitochondrion and into the endoplasmic reticulum where it is converted to P4 by 3β-hydroxysteroid dehydrogenase (HSD3B1), or to dehydroepiandrosterone (DHEA) by CYP17A1. P4 can either be released into the bloodstream or partially transformed into androstenedione (A4) by the enzyme CYP17A1. Additionally, DHEA can be converted into A4 by the enzyme HSD3B1. A4 is then transformed into testosterone (T) by HSD17B1 and T is converted to E2 by the aromatase CYP19A1. An alternative path to the last two steps is that A4 is first converted to estrone (E1) by CYP19A1, and E1 is then converted to E2 by HSD17B1. E2 is eventually secreted into the blood circulation.

The overall structure of the model is illustrated in Figure 3. It contains the essential steroid metabolites and feedback interactions between the pituitary gonadotropins FSH and LH and the ovarian hormones E2 and P4, the antral follicle and corpus luteum dynamics, and the steroidogenesis pathway. The steroidogenesis pathway contains several enzymatic steps. The initial rate-limiting step is marked with k_Des (desmolase; see Figure 3). All steroid conversion steps were modeled as mass-action processes, without explicit concern for the specific enzymes involved.

FSH stimulates the growth of the antral follicle. As the follicle increases in mass, its steroidogenic activity increases because more theca and granulosa cells are formed. This increase in steroid synthesis during follicle growth is implemented in the model by increasing the supply of plasma cholesterol as the substrate to the first step of the steroidogenesis pathway. As E2 in the plasma increases, it inhibits the production of pituitary FSH, causing FSH to decrease. Once the increase in plasma E2 reaches a certain threshold level, it triggers the release of LH, which was so far stored in the pituitary, into the blood circulation, producing a plasma LH surge. A similar surge is triggered in FSH.

The E2 threshold was implemented here by an intermediate signaling step (S, not shown) that functions as a bistable switch in response to E2. The LH surge converts the antral follicle to the corpus luteum, which eventually regresses. The corpus luteum drives the steroid synthesis also by increasing the supply of plasma cholesterol. In addition, the corpus luteum promotes the release of P4 to the circulation, causing a P4 rise in the luteal phase.

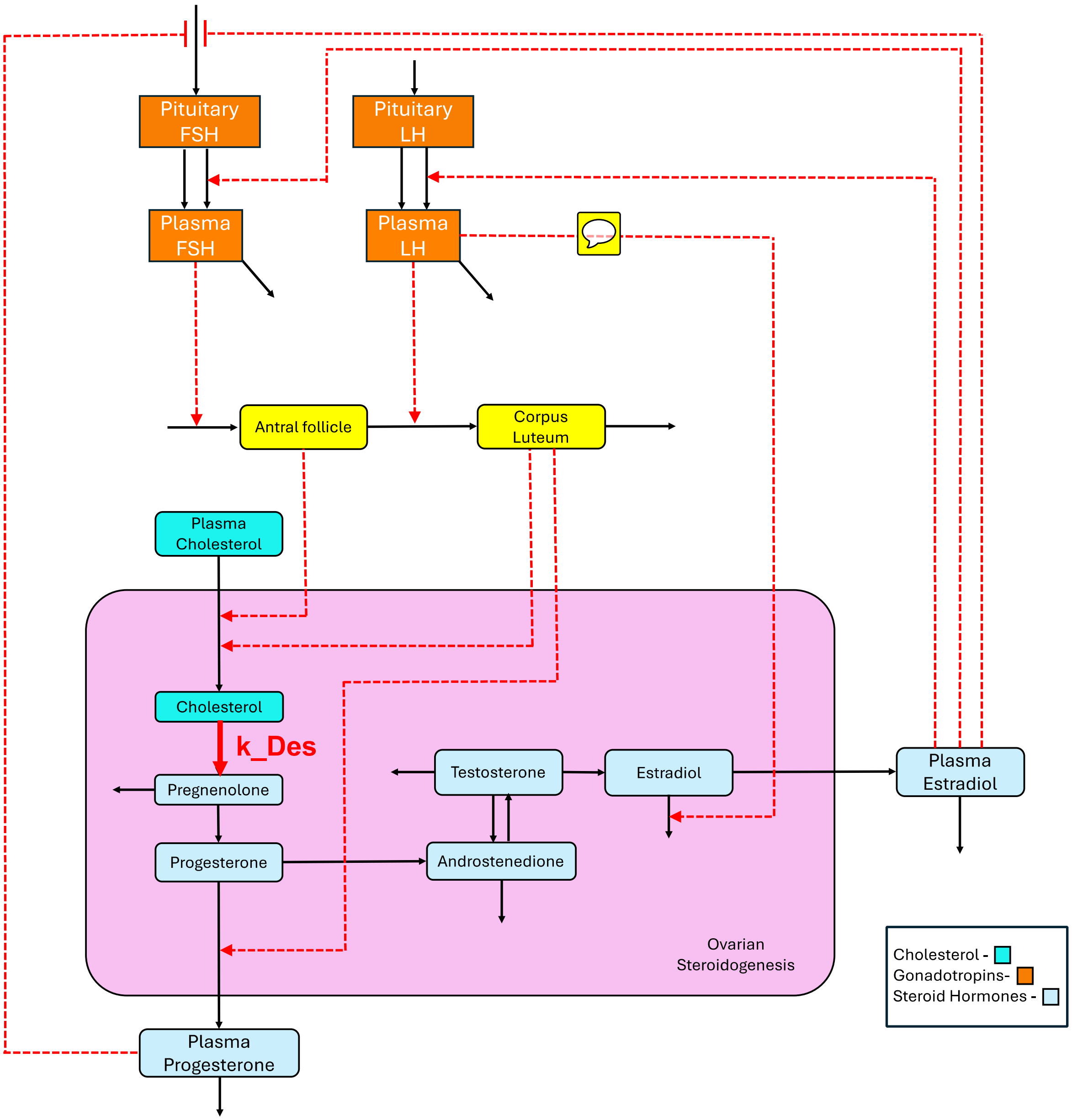

Figure 3. Anchor model of female steroidogenesis, describing the estradiol synthesis pathway, its regulation, and perturbation by dioxin. Molecules are color-coded by type, as indicated. Solid lines show reactions and transport steps, with the red solid line signifying the 20,22- desmolase reaction (k_Des), which is affected by dioxin. Dashed lines represent feedback regulation (red) from estradiol and progesterone on the synthesis and release of FSH and LH hormones from the pituitary to plasma, exerting control over the development of the antral follicles and the corpus luteum.

1.1.5. Effects of dioxin on the female reproductive system

Sufficient exposure to dioxin can cause a variety of adverse reproductive outcomes, including altered sex hormone dynamics, anovulation, reduced fecundity, delayed fetal growth and

development, and endometriosis [27]. Dioxin likely has multiple toxicity targets within the female reproductive system of a variety of species, but the most common appears to be inhibition of female steroid hormone production and disruption of the reproductive cycles. For instance, rhesus monkeys that had been exposed to 500 ppt dioxin in the diet for six months exhibited decreases in both E2 and P4 levels [28]. Similarly, chronic exposure to dioxin induced a dose-dependent decrease in the E2 level of proestrus in rats [29]. Dioxin also caused E2 to decrease in human luteinizing granulosa cells [30]. In mouse antral follicles, studied *in vitro*, dioxin exposure suppressed the secretion of P4, A5, T, and E2 over a period of 96 hours, showing a concentration-dependent pattern of inhibition [31, 32].

Exposure to dioxin in rats can interfere with the estrous cycle, resulting in an extended duration of the diestrus phase [33]. Similarly, rhesus monkeys exposed to dioxin through their diet exhibited a trend toward slightly longer menstrual cycle lengths, increasing from 27.3 ± 4.3 to 27.8 ± 3.9 days, although this average increase was not statistically significant [28]. Epidemiological studies showed that the menstrual cycle length of women living in the dioxin-polluted area of Seveso, Italy was positively associated with serum dioxin concentrations and that women with high serum dioxin concentrations experienced longer times to pregnancy and increased risk of infertility [34]. Serum levels of estrogenic polychlorinated biphenyls, some of which are dioxin-like compounds, were significantly associated with increased menstrual cycle length, which was prolonged by up to 3 days [35].

While animal and *in vitro* studies both showed that exposure to dioxin generally leads to suppression of ovarian E2 secretion, the steroidogenic enzymes altered by dioxin appear to vary depending on the species and experimental conditions. In human luteinizing granulosa cells, dioxin caused E2 to decrease without changing either the aromatase protein or its enzyme activity [36]; however, there was a decrease in CYP17A1 protein abundance and activity [37]. In these cells, it was shown recently that dioxin reduced mRNA expression of CYP11A1 and CYP19A1. Similarly, in granulosa cells from young or adult rats, dioxin lowered mRNA expression of the key enzyme CYP11A1 (20,22-desmolase) and of CYP19A1 [38]. In rats chronically exposed to dioxin, CYP17A1 mRNA expression was significantly inhibited while the expression of genes of other steroidogenic enzymes were not altered [29]. In mouse antral follicles, dioxin inhibited 20,22-desmolase, CYP17A1, HSD17B1, and CYP19A1 mRNA expression, and increased HSD3B1

mRNA expression; however, at the protein level only the abundance of HSD17B1 was significantly reduced [32].

In summary, sufficient exposure to dioxin can dysregulate several steroidogenic enzymes, inhibit steroid hormone production, and extend or disrupt the ovarian cycle.

2. Methods

A generic challenge of any modeling effort is that the true functional forms capturing the dynamics of complex biomedical systems are unknown and hardly identifiable with experimental means [39]. As a potent solution to this conundrum, we use canonical modeling with methods of Biochemical Systems Theory (BST) [39-41] and its most prominent special case, Mass-Action Kinetics (MAK). The resulting ordinary differential equations (ODEs) contain two types of parameters: (1) non-negative rate constants (v_{ij}) of production and degradation, and (2) kinetic orders (a_j), whose signs directly align with the modeled function. In MAK, the kinetic orders are non-negative integers (usually, 0, 1, or 2), while they may be any real numbers in general BST systems. Positive kinetic orders in BST identify augmenting or increasing influences, whereas negative kinetic orders signal inhibitory effects. BST is mathematically rigorous, as it is directly based on Taylor's approximation theory of numerical analysis.

2.1. Template Model

The T&A paradigm provides a structured framework for exploring both the overall dynamics of a complex system and the intricate details of its underlying sub-systems. The high-level dynamics of a system is captured in the template model. For the problem space of dioxin and its health effects, one could imagine a variety of template models. Due to the critical importance of cholesterol, we structure our template around the handling of cholesterol by different organ systems in the body. A pertinent organism-wide diagram is shown in **Figure 4**.

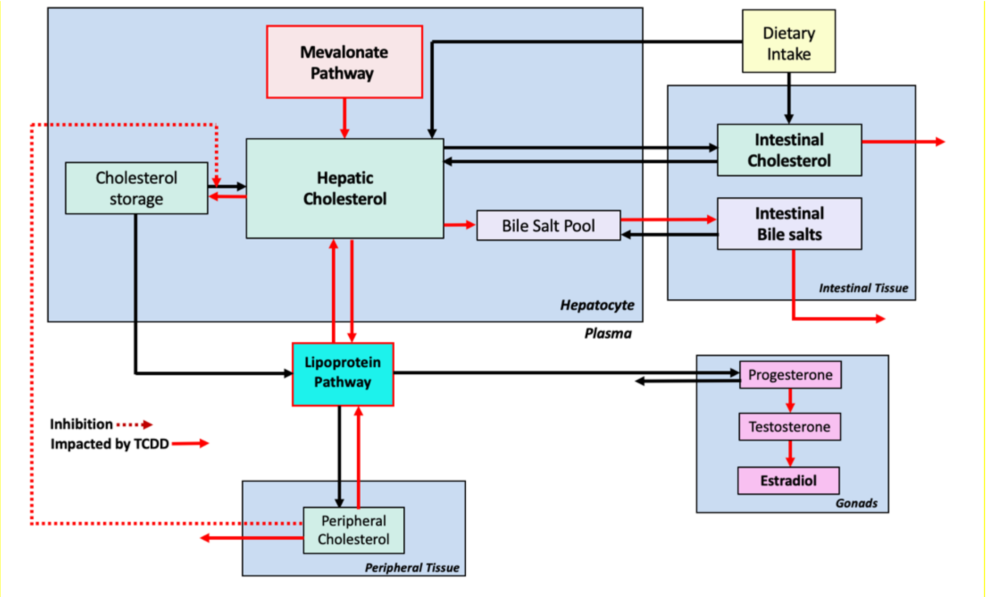

Figure 4. Template model of dioxin exposure affecting organism-wide cholesterol handling at a systemic level. Processes affected by dioxin exposure are indicated by red arrows. Adapted from [1].

The dependent (state) variables of a template model, rather than being conventional model variables like molecular concentrations, are the anchors, which themselves are models at a lower level of organization and collectively represent processes, such as cholesterol biosynthesis. In our template model, the interaction arrows indicate the handing over of cholesterol (in some form) or direct or indirect signaling events modulating cholesterol-associated processes within the various boxes. We will discuss these conceptual roles of variables and arrows later in more detail.

There is no general prescription for which variables should ultimately by expanded into full anchor models. We considered as anchors three systems that correspond to the most prominent processes of cholesterol handling:

1. Cholesterol biosynthesis in hepatocytes, combined with cholesterol storage through the reversible formation of cholesterol esters, and transport among liver, plasma, and peripheral tissues. This anchor model was discussed elsewhere in detail [1].
2. The pathway system governing the roles of the lipoproteins LDL, HDL, VLDL and IDL, which transport cholesterol within the bloodstream.
3. The dynamics of endocrine processes pertaining to the synthesis of progesterone, testosterone, estradiol, and related steroids in the gonads.

If deemed of benefit, one could explicitly consider further anchor models, which are presently not addressed or subsumed in the three anchors above. Examples are cholesterol storage and

utilization in peripheral tissues, which at this point are kept as template variables that are not expanded into full anchor models. This decision illustrates the flexibility of the T&A approach to zoom in or out from a different viewpoint.

The analysis of the template model allows a global assessment of the effects of dioxin on the human body, while analysis of the anchors captures the molecular details and mechanisms associated with cholesterol in the various organ systems. The results from analyzing the anchors and the effects of dioxin will ultimately be used to inform the parameterization and modulation of the template model, as described later.

2.1.1. Mathematical formulation of the template model

The first step of about any model design and analysis is the creation of a block diagram consisting of boxes and interaction arrows [42, 43], and T&A models are no exception. The T&A diagrams resemble diagrams that are common in control theory and biomathematics for modeling natural and engineered dynamical systems. The block diagrams in control theory schematically depict how an input signal to a system undergoes various transformations through blocks of transfer functions, including the potential forward flow of a signal or feedback signals. The transfer functions are typically modeled in the signal domain as the Laplace transforms of the mathematical functions that transmit an input signal to an output signal. Due to the assumed linearity of the engineered system, the transformations of an input signal to an output in a block diagram are relatively simple and may include feedback signals or the forward flow of information. Such signals could also apply to biomedical systems, but these are almost always nonlinear, thereby excluding Laplace transforms from the repertoire of applicable methods. Within the T&A context, both template and anchor models capture the dynamic behavior of processes that mutually interact in complicated ways.

Of importance in our context is that the variables of the template model, when zoomed in, are fully functional, more finely grained dynamic models, which serve as anchors. These anchor models provide a much more detailed view than typical block diagrams, and a visualization would require something like a nested block diagram, with nonlinear influences flowing up and down the hierarchy of the diagram.

To streamline the implementation of the dioxin model, we slightly simplify and rename the pathway system in Figure 4 toward the diagram in **Figure 5**, which is then mathematically

represented with nonlinear ordinary differential equations (ODEs) in the format of mass-action or generalized mass action (GMA) systems in BST [40, 41, 43, 44]. Using this format, and given a proper system diagram, developing a corresponding mathematical model is comparatively straightforward. Of particular note is that this strategy is effective even in the face of uncertainties. For instance, an observation like “if the value of Component A increases by this percentage, Component B decreases by this percentage” directly suggests a inverse relationship that can be captured well with a power-law function, that constitute the core of BST.

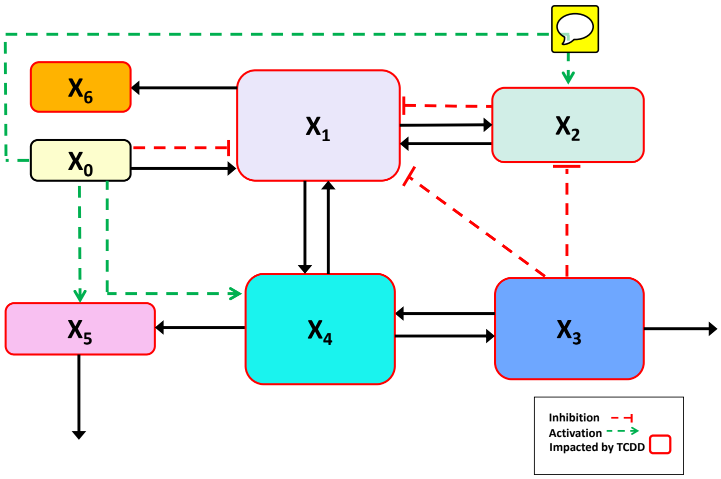

Figure 5. Simplified template model. The variables correspond to the processes in Figure 4. Specifically, X_0: Diet; X_1: Cholesterol biosynthesis; X_2: Cholesterol storage; X_3: Peripheral cholesterol utilization; X_4: Plasma transport and conversion of cholesterol; X_5: Steroid hormone production; X_6: Excretion.

Recalling that the variables in the template model represent processes, their steady-state values correspond to the steady-state fluxes of the anchor models, which may be directly affected by other template variables. The edges and their mathematical representations capture how the change in one template box (anchor) affects a neighboring box. If a box is the source of a signal, this signal is represented slightly differently than in typical models, because a signal here exerts its effect on other boxes, rather than on arrows, as it is typical in conventional models (**Figure 6**).

We begin the specific set-up of the template model by supposing the absence of dioxin exposure. The model described in the format of GMA equations is presented below. Even though the variables have a different meaning than one is used to, they contain the same two types of parameters: (1) non-negative rate constants (v_i) of production and degradation, and (2) kinetic orders (a_j), whose signs directly align with the modeled function: activating or augmenting

processes are represented with positive kinetic orders, while inhibitory or diminishing processes are modeled with negative kinetic orders.

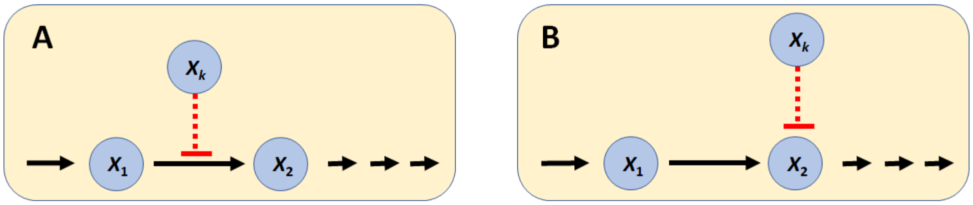

Figure 6. Signals in conventional models and T&A models. A: In a conventional model, variables modulate fluxes, either through activation or inhibition. B: In a T&A model, variables represent process systems that can directly affect each other.

The GMA representation of the template model in Figure 5 thus has the following format:

Biosynthesis

$$X_1{}' = v_{01} \cdot X_0{}^{a1} \cdot X_2{}^{a2} \cdot X_3{}^{a3} \cdot DR_1 + v_{41} \cdot X_3{}^{a4} \cdot X_4 \cdot DR_2 + v_{21} \cdot X_2 \cdot X_3{}^{a5} \cdot DR_3 - v_{14} \cdot X_0{}^{a1} \cdot X_1 \cdot DR_4 - v_{12} \cdot X_1 \cdot DR_5 - v_{16} \cdot X_1 \cdot DR_6 \quad (1)$$

Storage

$$X_2{}' = v_{12} \cdot X_1 \cdot DR_5 + v_{20} \cdot X_0{}^{a6} - v_{21} \cdot X_2 \cdot X_3{}^{a5} \cdot DR_3 \quad (2)$$

Cholesterol Utilization in Peripheral Tissue

$$X_3{}' = v_{43} \cdot X_4 \cdot DR_7 - v_{34} \cdot X_3 \cdot DR_8 - v_{30} \cdot X_3 \quad (3)$$

Plasma Dynamics

$$X_4{}' = v_{34} \cdot X_3 \cdot DR_8 - v_{43} \cdot X_4 \cdot DR_7 - v_{41} \cdot X_3{}^{a4} \cdot X_4 \cdot DR_2 + v_{14} \cdot X_0{}^{a1} \cdot X_1 \cdot DR_4 - v_{45} \cdot X_0{}^{a1} \cdot X_4 \cdot DR_9 \quad (4)$$

Steroid Hormone Production

$$X_5{}' = v_{45} \cdot X_0{}^{a1} \cdot X_4 \cdot DR_9 - v_{40} \cdot X_5 \quad (5)$$

In these equations, each quantity DR_i represents the effect of dioxin on a given process. At the baseline (dioxin absent ↔ TCDD = 0), all DR_i have a value of 1. For different dioxin exposure scenarios, the DR_i are numerically adjusted according to dose-response curves developed in the anchor models. Specifically, the amount of cholesterol handled under exposure to a dioxin dose of interest is extracted from the dose-response relationships obtained from the appropriate anchor models that are to be analyzed separately (see [1] and below). This modified amount of cholesterol is now handed off to other variables of the template model. In other words, dose-response relationships are used as dioxin-dependent modulators of the baseline fluxes under no exposure. They are applied to pertinent template variables, such as the biosynthesis pathway or cholesterol handling in the bloodstream (see Figures 1 and 2).

Once defined as an independent (external) variable, the dioxin dose can be increased incrementally across all known affected processes, where different degrees of dioxin effect can be represented with corresponding fold changes for different fluxes ($DR_i > 1$ or $DR_i < 1$) and magnitudes reflecting augmenting or diminishing effects. Thus, the template model at baseline (TCDD = 0) represents the normal, physiological state and dynamics of the cholesterol system. Once the anchors have been generated, importing dose-response information into the template allows us to gain a more comprehensive understanding of the systemic impact of dioxin on affected processes at the organismic level.

2.1.2. Parameter determination and model evaluation

If sufficient information were available regarding cholesterol sharing among organs, the template model could be parameterized from this information. However, such explicit information is lacking. Instead, the input/output relationships, obtained from the separately established anchor models, are used to inform steady-state values of the template variables and the connections between them. Kinetic-order parameters which, for instance, describe the influence of estradiol signaling and peripheral tissue utilization on cholesterol uptake in hepatocytes, as well as hepatic cholesterol storage, are then adjusted to render output consistent with published steady-state concentration data.

Upon calibrating the model parameters to match the steady-state concentrations that have been reported for the anchor models, the output of the template model at baseline (TCDD = 0) exactly reflects the steady-state fluxes in the anchor models. Specifically, the fluxes for

biosynthesis, storage, tissue utilization, plasma transport, and steroidogenesis are 18,150, 0.56, 266, 3,266, and 1.34 μg/kg, respectively.

2.2. Anchor Model of Cholesterol Biosynthesis

The biosynthesis anchor model was analyzed and discussed elsewhere in detail [1]. It contains as variables the metabolites of the pathway, as well as pertinent mRNAs, enzymes, and the important transcription factor SREBP, which is controlled by the amount of hepatic cholesterol through feedback inhibition (Figure 1). Using methods of BST and MAK, it was relatively straightforward to convert the diagram of the mevalonate pathway into a dynamic mathematical anchor model (see, *e.g.*, [39, 41, 45]). Literature searches directly or indirectly yielded parameter values, as well as information quantifying the effect of dioxin. Model equations are revisited in the *Supplements*. The biosynthesis model is also available on GitHub (GitHub.com/LBSA-VoitLab/TCDD_Chol) and has been submitted to Biomodels.

As a side note, our analysis [1] actually targeted two models of biosynthesis: One without interactions with plasma, and one where the pathway was combined with a simplified representation of cholesterol transport between the liver and plasma. This use of alternative anchors illustrates the flexibility of the T&A approach in terms of substitutions of anchors with models that are deemed more appropriate for a specific purpose.

2.3. Anchor Model of Lipoprotein Metabolism and Cholesterol Transport

As with the biosynthesis pathway, we used methods of BST to model the roles of lipoproteins. BST permits a direct conversion of the pathway diagram into symbolic equations [45]. Typical examples are the ODEs for LDL and HDL:

LDL Cholesterol:

$$LDL' = c_{11} \cdot HDL - c_8 \cdot LDL + c_{21} \cdot IDL - c_{22} \cdot LDL \qquad (6)$$

HDL Cholesterol:

$$HDL' = c_{13} \cdot LCAT^{k_3} - c_9 \cdot HDL - c_{11} \cdot HDL - c_{12} \cdot HDL \qquad (7)$$

Here, all kinetic orders are set to 1, which is typical for mass-action and transport models. The indexed quantities DR_i are placeholders that have a value of 1 at the baseline (TCDD = 0) but allow us later to modify rates according to process-specific dose-response relationships of dioxin exposure.

The complete set of ODEs can be found in the *Supplements*. Computer code in PLAS and MATLAB format is available on GitHub.com/LBSA-VoitLab/TCDD_Chol.

2.3.1. Parameter determination and model evaluation

Although the model is fully dynamic, it is evaluated here at the steady state because we are only interested in the long-term effects of dioxin exposure, which implies that the system has had enough time to reach a steady state. As a consequence, one rate in each equation, determining the time scale of the dynamics of the variable, is more or less arbitrary and may be set to a reasonable value (see below). The remaining parameters were set such that the model precisely matched the steady-state concentrations in human hepatocytes or plasma, as reported in the literature, namely: hepatic cholesterol: 11,636 μM [46]; VLDL: 776 μM [47]; LDL: 2,586 μM [47]; HDL: 1293 μM [47]; IDL: 388 μM [47]. Upon calibrating the model parameters, the steady-state lipoprotein concentrations obtained from the literature and those computed with the parameterized model were quite similar to each other. Basal protein activity levels were arbitrarily defined as 100, reflecting "normal conditions." Values of other model parameters were based on previously obtained parameters from the mevalonate anchor model [1] and from literature information; they were reasonably adjusted to obtain model outputs consistent with published steady-state concentration data. The remaining parameter values were manually adjusted to align approximately with the expected half-lives of the lipoproteins and enzymes. Finally, we calibrated the percent change in lipoprotein cholesterol concentration under dioxin exposure based on animal studies for a dioxin dose of 30 μg/kg [48]. For simplicity, and due to the lack of specific information, we then used interpolation to obtain lipoprotein cholesterol concentrations at other doses of dioxin. A summary of the parameter values for the model is provided in the *Supplements, Section* S2.2.

Using the methods presented in *Supplements, Section* S1 with the parameter values listed in *Supplements, Section* S2.2, numerical simulations and sensitivity analyses were conducted to examine the impact of various doses of dioxin exposure on hepatic and lipoprotein cholesterol homeostasis. The sensitivity analysis did not reveal any concerns, as all parameter sensitivities

with respect to steady-state concentrations were less than 1 in magnitude (see *Supplements, Section* S8).

2.4. Anchor Model of Ovarian Steroidogenesis

We set up a dynamic model reflecting steroidogenesis, as depicted in Figure 3. It can be found in the *Supplements* and GitHub.com/LBSA-VoitLab/TCDD_Chol. The system was formulated as an anchor model in the format of mass action, Michaelis-Menten, or Hill kinetics. The reactions were parameterized to match observed fold-changes in variables. The ODEs and parameter values of the model are listed in the *Supplements, Section* S2.3. The key model output (**Figure 7**) recapitulates the hormonal dynamics in the follicular and luteal phases semi-quantitatively [49, 50].

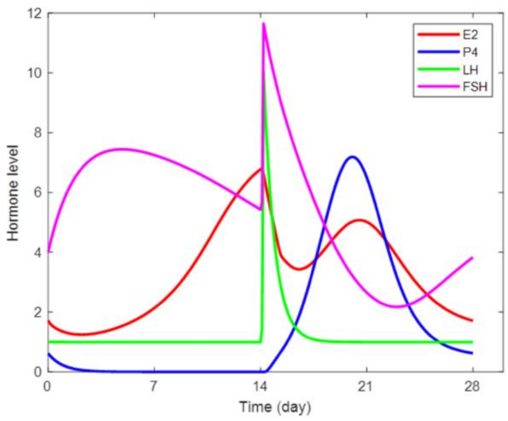

Figure 7. Simulation results with the steroidogenesis anchor model. The output captures the dynamics of gonadotropins (FSH and LH) and steroid hormones (E2 and P4) throughout one menstrual cycle.

The steroidogenic enzyme targets of dioxin vary with species and the state within the ovarian cells [31, 32, 36, 38, 51]. This fact renders it challenging to implement the inhibitory effect of dioxin in an enzyme-specific manner. To address this issue, we collectively assess the effect by modulating just the rate-limiting step of the steroidogenesis pathway, which is the conversion of cholesterol to P5 by the enzyme 20,22-desmolase (k_Des; Figure 3). This simplification is in line with research showing that the inhibitory effects of dioxin on P4, A4, T, and E2 in mouse antral follicles can be reversed completely by oversupplying P5, suggesting that the main inhibition site of dioxin is upstream of P5 [32]. Thus, we simulated the effect of dioxin by altering k_Des to different fold-change levels relative to its default value. This linking of dioxin exposure and k_Des activity is furthermore supported by epidemiological information. Namely, a study [34]

investigated the association between menstrual cycle length and serum dioxin level in young women who were exposed to dioxin caused by an industrial explosion. These women had not reached puberty at the time of the incident and had subsequently lived in the same area for 20 years. The study showed that for every 10-fold increase in serum dioxin concentration, the menstrual cycle was lengthened by 0.92 days. With the serum dioxin ranging between 10-10,000 part per trillion (ppt) in these subjects, the mean menstrual length varied by nearly 3 days.

Assuming that the increase in menstrual cycle length was caused primarily by an increase in the duration of the follicular phase, we implemented the dioxin dose-response by specifying a Hill function between dioxin concentration and the fold-change in k_Des activity so that high dioxin concentrations lowered the activity of k_Des to a value causing the follicular phase length to increase by 3 days.

To mimic epidemiological findings, representing distributions of health effects within populations, we ran Monte Carlo simulations with steroidogenesis model, where key parameter values were randomly sampled from pre-defined lognormal distributions.

3. Results

3.1. Anchor Model of Cholesterol Biosynthesis

We previously examined the dioxin-mediated effects on hepatic and serum cholesterol homeostasis at various doses of exposure [1]. Additionally, we investigated the impact of varying levels of dietary cholesterol intake on the system.

The main result of this anchor analysis was that, despite a *decrease* in hepatic cholesterol synthesis due to dioxin exposure, the overall level of hepatic cholesterol *increased*. This at first surprising increase was shown to be attributable to increased transport of cholesterol from the plasma to the liver. Additionally, dietary cholesterol intake was demonstrated to have a more pronounced effect on hepatic cholesterol levels compared to plasma levels. Combined, dioxin exposure and a high-cholesterol diet were shown to lead to an even greater accumulation of hepatic cholesterol, highlighting the compounded impact of environmental toxins and dietary factors on liver cholesterol homeostasis.

Various simulation scenarios are possible with this anchor model. For instance, we may simulate different magnitudes of dioxin exposure, analyze the effects of varying dietary cholesterol intake on the system, and test how these variations, combined with dioxin exposure, impact hepatic

and serum cholesterol levels [1]. As an illustration, we present below one model result of an analysis of a series of dioxin concentrations on the steady-state concentration of hepatic cholesterol. Interpolating these results yields a dose-response relationship between dioxin and the anchor's main output variable, hepatic cholesterol (**Figure 8**).

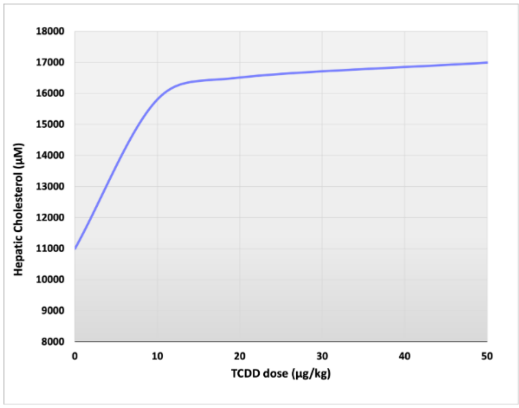

Figure 8. Simulation results from the biosynthesis anchor analysis. The model results were formulated as a dose-response relationship characterizing the effect of dioxin on the steady-state amount of hepatic cholesterol. Adapted from [1].

The amount of cholesterol produced under continuous exposure to any relevant dioxin dose of interest can be extracted from this dose-response relationship and will eventually be handed off to other variables of the template model. Specifically, the dose-response relationship will be used as a dioxin-dependent modulator of the healthy baseline flux (TCDD = 0) between the mevalonate entering the pathway and hepatic cholesterol level (see Figure 4).

3.2. Anchor Model of Cholesterol Transport and Metabolism in the Plasma

3.2.1. Effects of dioxin on cholesterol transport in plasma

To evaluate the influence of dioxin on the lipoprotein transport system, we assessed the effects of varying doses of dioxin, spanning a range from 0 to 50 μg/kg body weight. Specifically, the assessment was executed by adjusting the parameters DR_i for each dioxin-impacted flux (see *Methods*) such that the outcome matched literature information, which however was only available for a dose of 30 μg /kg [48]. We then calibrated the model using the previously developed dose-response curve for dioxin versus hepatic cholesterol [1].

In response to these dose-specific adjustments, the total plasma cholesterol, LDL, and HDL all decreased by roughly 25% following treatment with 30 μg /kg dioxin. At the same time, the

gradual "loss" of plasma cholesterol with increasing dioxin levels turned out to be accompanied by strong increases in hepatic cholesterol, as they have been observed [1] (**Figure 9**).

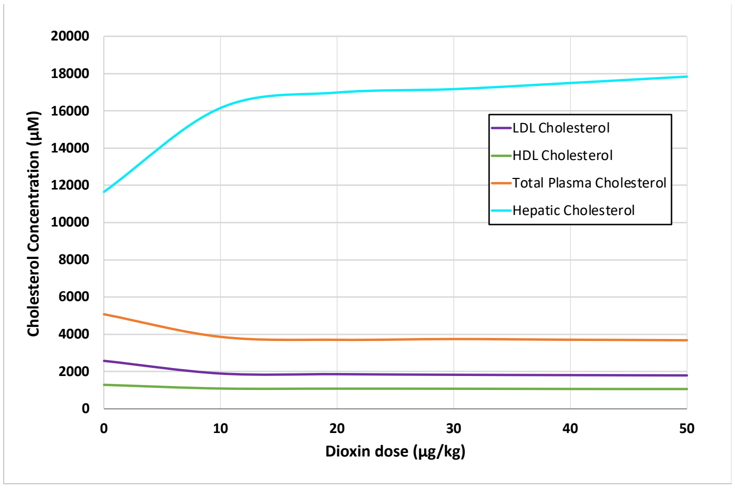

Figure 9. Dose-response curves quantifying the effects of increasing dosage of dioxin on hepatic, and plasma LDL, HDL, and total cholesterol concentrations.

Thus, similar to the dose-response curves previously reported for the cholesterol biosynthesis anchor model (Figure 8, [1]), the dioxin effect on hepatic cholesterol accumulation is noticeable even for low exposures and approaches saturation for high exposures. This increase in hepatic cholesterol under higher doses of dioxin is accompanied by a concomitant reduction in the concentrations of total plasma cholesterol, HDL, and LDL. Specifically, a dose of 30 μg/kg/day leads to an approximate 26% decrease of total plasma cholesterol, 17% decrease of HDL, and 30% decrease of LDL, which aligns well with observations in rodents [48].

3.2.2. Effects of dietary cholesterol on LDL and HDL

The model allows us to assess the potentially synergistic role of dietary cholesterol intake and its effects on hepatic and serum cholesterol. The recommended range of dietary cholesterol intake is approximately 200 mg/day, while high intake can reach 600 mg/day [52]. The model indicates that dietary cholesterol intake increases hepatic, VLDL, IDL, HDL, LDL, and total plasma cholesterol (**Figure 10**), which is consistent with literature reports [53].

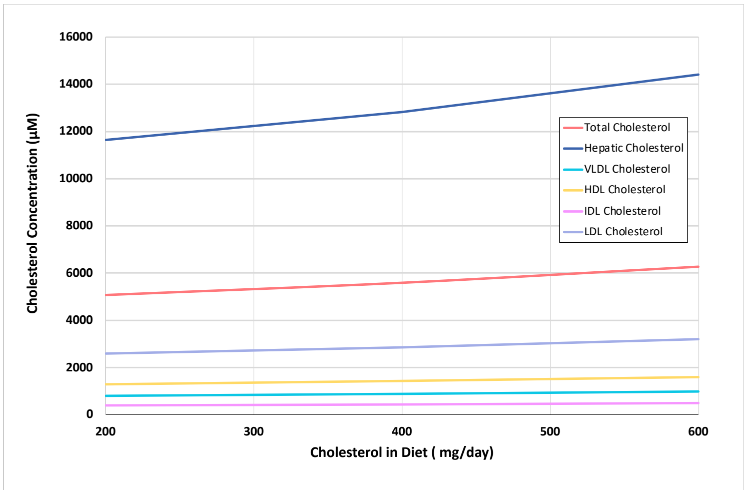

Figure 10. Effect of increased dietary cholesterol intake on LDL, HDL, IDL, VLDL, as well as on hepatic and plasma cholesterol concentrations, according to the model.

Using these results, we explored with simulations the effects of combinations of dietary intake and dioxin exposure. Without dioxin exposure, the steady-state values of LDL and HDL cholesterol under a normal daily consumption of ~200 mg cholesterol are about 2,600 µM and 1,300 µM respectively. As expected, both LDL and HDL cholesterol levels increase with increasing dietary intake but show a decrease with increasing doses of dioxin (**Figure 11**). For the recommended cholesterol intake, the model predicts a decrease in LDL and HDL to 1,782 µM and 1,064 µM respectively, if the individual is exposed to a relatively high dose of 50 µg/kg of dioxin. In comparison, for the same dose of dioxin, but with 600 mg/day of dietary cholesterol intake, the model predicts approximately 2,200 µM and 1,315 µM of LDL and HDL cholesterol respectively, which is close to the concentrations observed with the recommended dietary intake but with no dioxin exposure. Again, these decreases are accompanied by increases in hepatic cholesterol.

3.3. Anchor Model of Ovarian Steroidogenesis

3.3.1. Average model of the menstrual cycle

Figures 3 and 7 depict the interplay of sex hormones over the course of a menstrual cycle, capturing the fluctuations of the key steroid hormones—E2 and P4—as well as the gonadotropins—FSH and LH. The model of this system was calibrated using literature information that benchmarks the average menstrual cycle at 28 days, with ovulation typically occurring around day 15 shortly after the LH surge. The model allows us to track the progression of E2, P4, FSH, and LH during the

follicular phase from days 1 to 15, leading up to ovulation, and subsequently through the luteal phase from days 15 to 28.

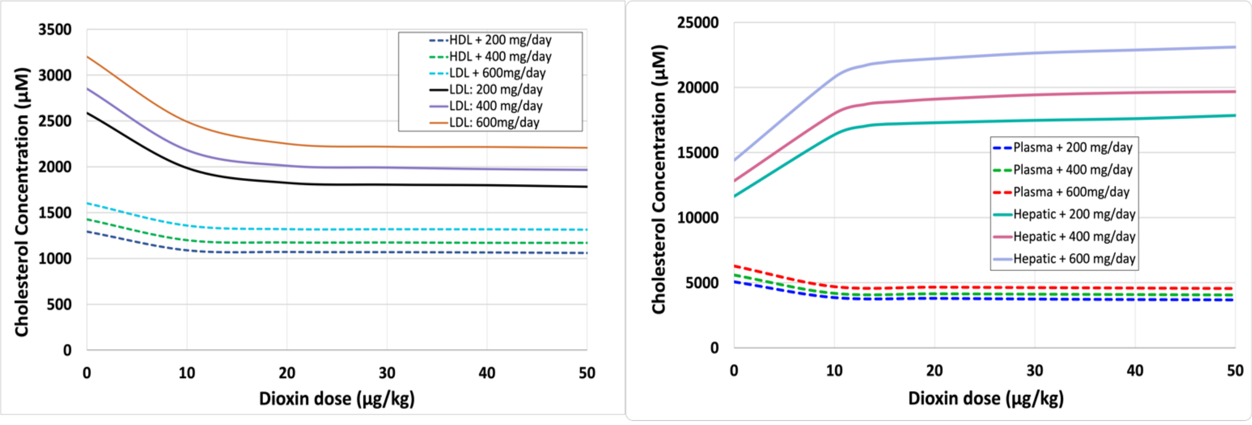

Figure 11. Dose response curves accounting for the effects of dioxin and increased diet on the lipoprotein transport system. Left: lipoproteins LDL and HDL. Right: Hepatic and plasma cholesterol. 200 mg/ day is the recommended amount of cholesterol in diet/day while 400 mg/day is high and 600 mg/day is considered very high.

3.3.2. Population-based modeling of menstrual cycle variability

The menstrual cycle length varies considerably within a population, due to both intra- and inter-individual differences [54-57], and particularly in the duration of the follicular phase [56, 58]. Furthermore, there is a broad range of what constitutes “normal” hormonal levels across different individuals [49, 50]. Considering the documented effects of dioxin on menstrual cycle duration and estradiol levels, we constructed a population-based model via Monte Carlo simulation, in which key parameters were randomized. These parameters quantify, respectively: the affinity constant reflecting the E2 threshold that triggers the LH surge (J_{16}), the clearance rates of estradiol (k_{10}) and progesterone (k_{14}) from the plasma; the rate of LH transport from pituitary into plasma (k_{18}), as well as the rate of antral follicle growth (k_{21}) (see *Supplements*, S2.3). The Monte Carlo simulation thereby accounts for variations and provides us with deeper insights into population-wide effects of dioxin on ovarian steroidogenesis. The dynamic trends of E2, P4, FSH, and LH within the synthetic population are presented in **Figure 12** across the menstrual cycle. The simulation accounts for 100 individuals (grey). All trends retain their essential shapes but now exhibit variability. The figure shows individual trends (grey) as well as the means (solid colored lines) and 95% ranges (dashed lines).

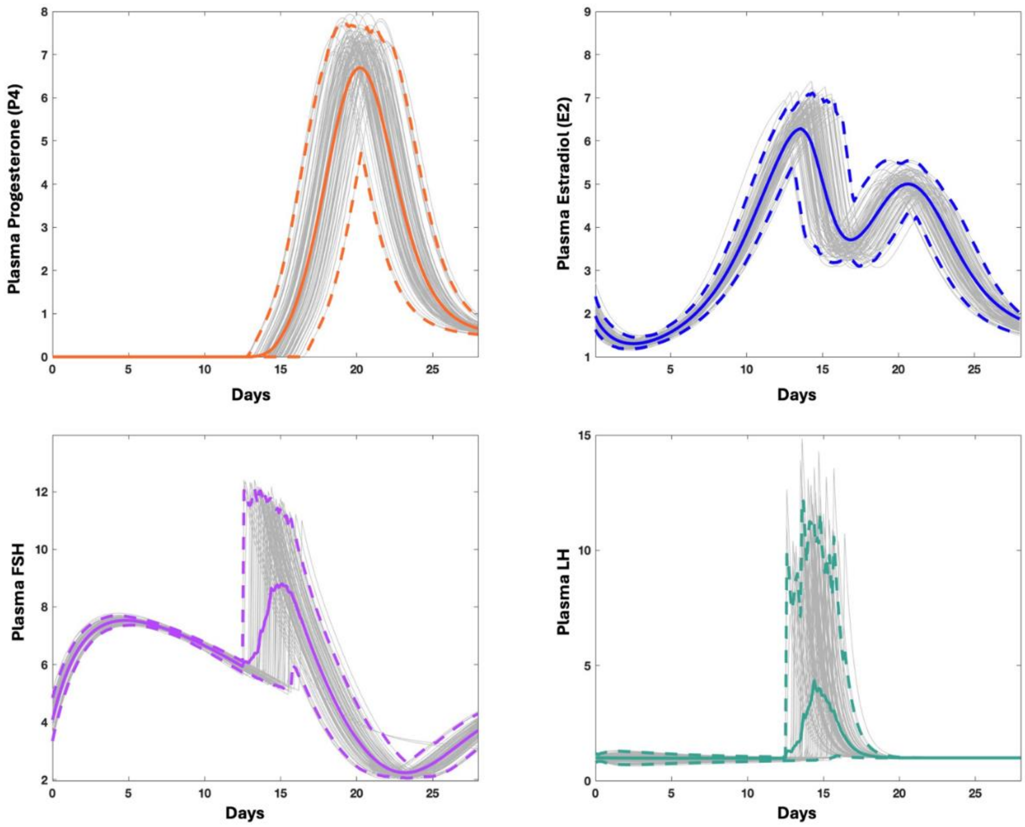

Figure 12. Dynamics of gonadotropins (FSH and LH) and steroid hormones (E2 and P4) throughout the menstrual cycle in a population of individuals. Grey lines represent the data for 100 individuals. The solid lines denote the average values across these cases, while the dashed lines define the ranges within which 95% of the values lie.

3.3.3. Impact of dioxin exposure on the average model of the menstrual cycle

We evaluated the influence of dioxin on steroidogenesis by lowering the rate of the rate-limiting conversion of cholesterol to P5, which is catalyzed by 20,22-desmolase (k_Des; see *Methods*). Specifically, we subjected k_Des to different fold-change levels relative to the default value as indicated in **Figure 13**. For instance, a 20% reduction in k_Des corresponds to a commensurate 21% reduction in the peak E2 level, but it also delays the LH peak occurrence from the original day 14 to day 16.

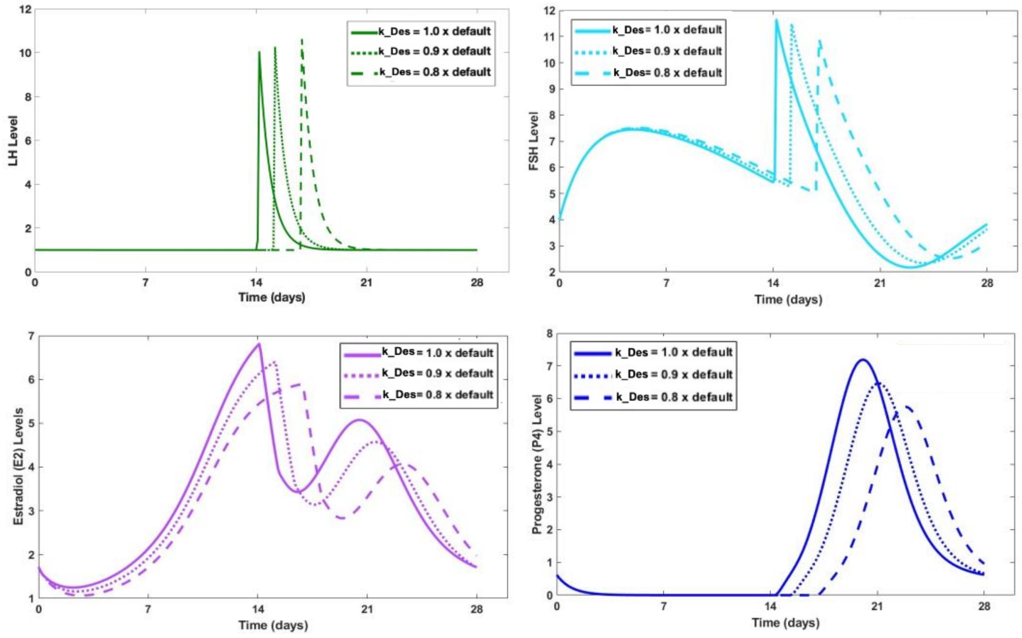

Figure 13. Effects of varying k_Des activity (*i.e.*, conversion of cholesterol to P5 by 20,22-desmolase) on hormone dynamics based on the standardized menstrual cycle model.

3.3.4. Effect of dioxin in the population model

We quantified the population-wide impact of dioxin on steroidogenesis, again per Monte Carlo simulation, for fold-decrease in k_Des. The results suggest that the lengths of the follicular phase exhibit a right-skewed distribution, as depicted in **Figure 14**, with the mean increasing as dioxin levels rise. Within the serum dioxin concentration range of 1 – 10,000 ppt, the follicular phase lengthens by approximately 3 days, starting from a mean of 14.4 ± 0.8 days to 17.3 ± 2.4 days, accompanied by increased variability, which includes instances of individuals displaying a 24-day follicular phase. The cumulative probability distribution of follicular phase lengths skews rightward as dioxin levels rise, suggesting that dioxin exposure impacts nearly the entire population under study.

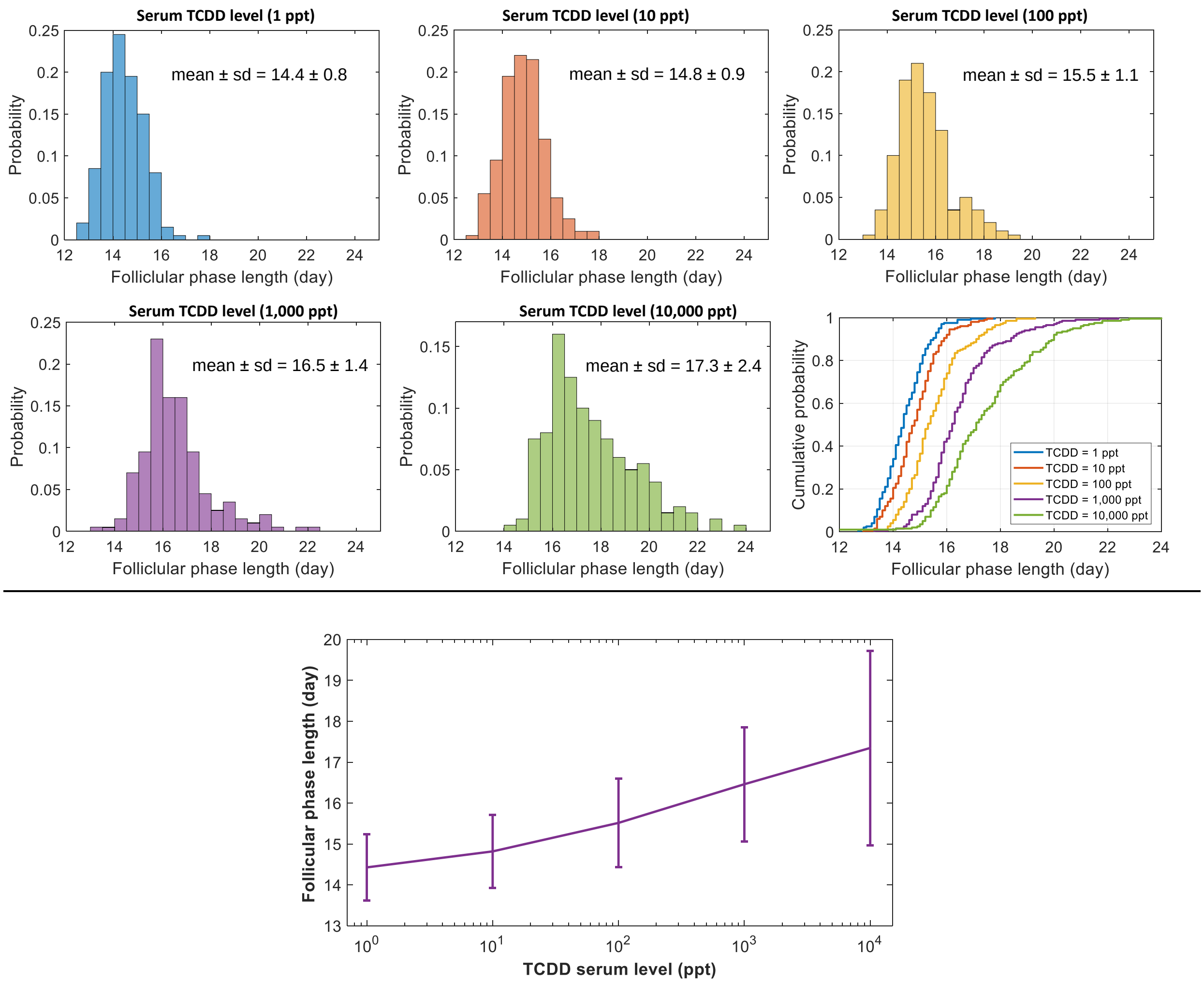

Figure 14 Population-wide distributions of follicular phase lengths and corresponding dose-response curves. Top panels: Frequency distributions and corresponding cumulative probabilities describing the relationship between dioxin serum levels (ppt) and follicular phase length. Bottom panel: Dose-response curve for a population, depicting the influence of dioxin serum level on the length of the follicular phase, expressed as the mean ± standard deviation.

3.4. Template Model

3.4.1. Overall Impact of Dioxin

To quantify the impact of dioxin on cholesterol biosynthesis in the template model, we analyzed the effects of different doses of dioxin ranging from 0 to 50 μg/kg body weight. To this end, the impact of dioxin on the overall rate of each anchor was incorporated into the template model through adjustments of the pertinent DR_i quantities, as discussed in the *Methods* section and demonstrated before [1]. The DR_i values were obtained from the dose-response curves of the anchor models. For instance, the alterations in rates of hepatic cholesterol biosynthesis were

recorded and used to construct a dose-response (DR) relationship for the template model (**Figure 15**). Using this DR, we were able to predict the effects of dioxin on the various processes comprising the template model (Table 2).

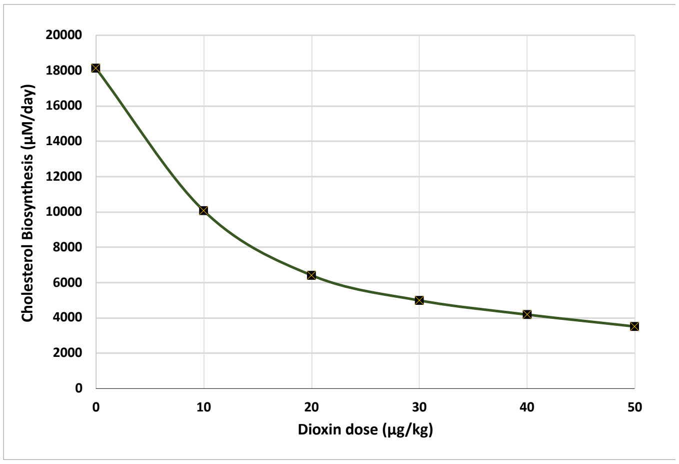

Figure 15. Dose response curve of dioxin *versus* cholesterol biosynthesis, according to the template model.

Table 2. Dose-response relationships of the processes *versus* dioxin dose. Sections highlighted in grey represent simulation-based predictions generated by applying values of the dose-response curve for cholesterol biosynthesis (unshaded; Figure 8) to the entire template model, including hepatic cholesterol storage, peripheral tissue usage, plasma dynamics, and steroid hormone production.

	Dioxin Dose					
	0 µg/kg	10 µg/kg	20 µg/kg	30 µg/kg	40 µg/kg	50 µg/kg
Biosynthesis	18150	10081	6424	5641	4196	3525
Storage	0.56	0.60	0.62	0.63	0.65	0.66
Peripheral Tissue Utilization	266	348	406	421	452	468
Plasma Dynamics	3266	2300	1663	1503	1180	1017
Steroid Hormone Production	1.34	0.94	0.68	0.62	0.48	0.42

With increasing doses of dioxin, the rate of cholesterol biosynthesis decreases [1], in line with what is documented in the literature (*e.g.*, [59]. The observed decline can be explained by the suppressed expression of multiple genes involved in biosynthesis. Increasing doses of dioxin are predicted by the template to raise peripheral cholesterol utilization and hepatic storage, while concurrently causing a reduction in plasma transport. These predictions are yet to be validated (or refuted) experimentally.

The decrease in biosynthesis is paralleled by a predicted decrease in sex hormone production. This model prediction aligns well with animal studies that indicate reduced estradiol

production and with epidemiological studies associating lower estradiol levels with dioxin exposure in humans [34].

In contrast to biosynthesis and steroidogenesis, the model predicts an increase in the utilization of cholesterol in peripheral tissues. This predicted change is again consistent with the literature [60] and can also indirectly be related to human health, where such exposure has been linked to the development of ischemic heart disease [61], a condition precipitated by the accumulation of cholesterol within the coronary artery lining [62].

3.4.2. Impact of diet on the template model

The template model allows us to assess the role of dietary cholesterol intake and its interaction with dioxin exposure on a broader scale. We begin with the effect of dioxin at the baseline of TCDD = 0.

The template model indicates that an increase in cholesterol intake results in a decrease of cholesterol synthesis in the liver. A possible explanation is that the increase in hepatic cholesterol, as a result of increased dietary cholesterol intake, promotes the reduction of cholesterol synthesis enzymes through the down-regulation of SREBP [63]. Our simulations with the template model reveal a relationship between increased cholesterol in diet and decreased biosynthesis rate (Table 3), which is consistent with the results found in the cholesterol biosynthesis anchor model detailed in [1].

Table 3: Impact of diet on the various processes of the template model. The sections highlighted in grey represent simulation-based predictions generated by applying the dose-response curve for cholesterol biosynthesis (unshaded) to the template model.

Diet (mg)	Biosynthesis	Storage	Peripheral Tissue Utilization	Plasma Dynamics	Steroid Hormone Production
**200**	18150	0.56	266	3266	1.34
**400**	15525	0.67	233	2859	1.66
**600**	14154	0.74	215	2634	1.87

Overall, the template model indicates a decrease in cholesterol biosynthesis and plasma dynamics, accompanied by an increase in cholesterol storage and peripheral cholesterol utilization. At the same time, there is a significant decrease in steroid hormone production. A hand-waving explanation for this apparently counterintuitive decrease in flux rates in response to increased dietary intake is the availability of cholesterol from diet: The body does not need to produce as

much cholesterol *de novo* if it receives it "for free" from the diet. This conclusion is in line with a documented link between heightened dietary cholesterol intake and the suppression of LDL receptor (LDLR) activity, which mediates the transport of cholesterol between the plasma and liver [64]. Specifically, the transport of LDL cholesterol to peripheral tissues has been found to be suppressed under rising cholesterol intake, likely due to the fact that the levels of cholesterol in those tissues are already increased [65]. Moreover, it has been found that heightened dietary cholesterol intake is correlated with an elevation of cholesterol in peripheral tissues [66]. It is postulated that this elevation may serve as a feedback inhibitor to cholesterol storage, thereby mitigating any further accumulation of cholesterol within these peripheral tissues. However, with strongly increased dietary cholesterol, a corresponding increase in cholesterol storage may still be observed [67].

3.4.5. Combined impact of dietary cholesterol and dioxin exposure

We used template simulations to explore the effects of combinations of dietary intake and dioxin exposure. Without dioxin exposure, the rate of hepatic cholesterol synthesis, under a normal daily consumption of ~200 mg cholesterol, is about 18,150 μM/day (Table 4). As expected, the synthesis of hepatic cholesterol decreases with both increased dietary intake and increasing doses of dioxin, due to the accumulation of hepatic cholesterol (Figure 8). For the recommended cholesterol intake, the model predicts a decrease in the synthesis rate of cholesterol from 18,150 if an individual is exposed to no dioxin to about 3,525 μM/day, if exposure to high dose (~50 μg/kg) of dioxin occurs. For the same dose of dioxin, but with 600 mg/day of dietary cholesterol intake, the model predicts a synthesis rate of approximately 2,683 μM/day (Table 4).

Table 4: Impact of diet and dioxin on the processes comprising the template model. Sections highlighted in grey represent simulation-based predictions generated by applying values of the dose-response curve for cholesterol biosynthesis (unshaded) to the template model.

	Dioxin Dose					
	0 µg/kg	10 µg/kg	20 µg/kg	30 µg/kg	40 µg/kg	50 µg/kg
**200 mg Cholesterol**						
Biosynthesis	18150	10081	6424	5641	4196	3525
Storage	0.56	0.60	0.62	0.63	0.65	0.66
Peripheral Tissue Utilization	266	348	406	421	452	468
Plasma Dynamics	3266	2300	1663	1503	1180	1017
Steroid Hormone Production	1.34	0.94	0.68	0.62	0.48	0.42
**400 mg Cholesterol**						
Biosynthesis	15525	8572	5439	4771	3541	2970
Storage	0.67	0.71	0.74	0.74	0.76	0.77
Peripheral Tissue Utilization	233	305	356	369	396	410
Plasma Dynamics	2859	2016	1458	1318	1035	892
Steroid Hormone Production	1.66	1.17	0.84	0.76	0.60	0.52
**600 mg Cholesterol**						
Biosynthesis	14154	7787	4928	4320	3201	2683
Storage	0.74	0.78	0.81	0.82	0.84	0.85
Peripheral Tissue Utilization	215	281	328	340	365	377
Plasma Dynamics	2634	1857	1342	1213	952	820
Steroid Hormone Production	1.87	1.32	0.95	0.86	0.68	0.58

The sections highlighted in grey within Table 4 present simulation-based predictions generated by the simultaneous introduction of dioxin and increased dietary cholesterol intake.

Note that all results pertain to changes in processes and reveal no information regarding concentrations. To gain insight into changes in concentrations or amounts of cholesterol in different tissues, we need to explore the theoretical structure of T&A models.

4. Theoretical Considerations Regarding the Structure and Interpretation of T&A Results

We have been using T&A models in an intuitive manner, adopting the notion of a template model as a high-level, coarse-grained umbrella structure and the anchors as models elucidating parts of the template in greater detail. At first glance, the mathematical structures of template and anchor models seem to be simple variations of the same theme. Closer inspection, however, reveals an interesting combination of differences and correspondences between template and anchor models in general. This combination is most easily seen for a dynamic system operating at a steady state.

4.1. Analogy with Dual Graphs

At the steady state, the structures of anchor and template models conceptually resemble "dual graphs" (*e.g.*, [68]). The topic of dual graphs has been discussed in the graph theory literature for a long time, using different terms, primarily "line graphs," but also "edge graphs" and "edge-to-vertex duals," among others. Line graphs were arguably first mentioned by Whitney in 1931/1932 [69]. A good review is the 1972 book by Harary and, in particular, the chapter on line graphs [70].

In the language of graph theory, the line graph G* of a graph G is constructed by exchanging edges and vertices. In our case, the model structures at steady state are directed graphs ("digraphs"), and the analogy in this situation is therefore called a "line digraph" [70, 71]. For directed graphs, the vertex set of the line digraph G* corresponds to the edge set of G, and every directed edge in G* corresponds to an instance in G where the head of an edge e_i meets the tail of another edge e_j. **Figure 16** shows a generic example.

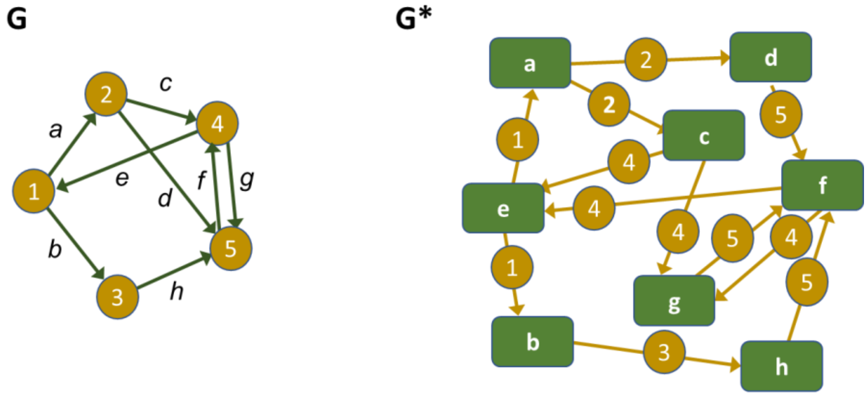

Figure 16. Example of a directed line graph G and its (edge-to-vertex) dual graph G*. The vertices of G* correspond directly to the edges in G, as indicated by letters a – h. The edges of G* correspond to vertices in G if one edge is entering this vertex and another one is leaving it. For instance, vertex 2 in G is represented in the directed dual graph G* through the edges from a to c and from a to d, because these edges meet head to tail at vertex 2 in G.

In contrast to static graph theory, the literature on dual dynamic models is scattered, with rather different concepts using the term "dual" (*e.g.*, [72-74]). Closest in spirit to the situation of T&A models is an article on flux duality [75], where the exchange of variables and fluxes in certain types of primal and dual differential equation models was investigated. One must emphasize at this point that the notion of line graphs or line digraphs is only an analogy to our situation of T&A

models. Nevertheless, this analogy helps us set up anchors and templates in an appropriate manner, as described below.

A typical anchor model has the underlying graph structure of a typical metabolic or physiological model. For instance, the variables (vertices) of the cholesterol biosynthesis model (Figure 1) are metabolites, and the fluxes (directed edges) among them represent (possibly regulated) biochemical reactions, transport steps, or the formation or disassociation of complexes. This type of diagram represents the biosynthesis anchor well and, as a natural consequence of the typical model design applied here, there is perfect flux balance at any steady state: the fluxes into any of the variables collectively equal the fluxes leaving this variable in magnitude.

Surprisingly, these fundamental features are different in a template model. The variables (vertices) in a template model are usually physiological or biochemical *processes*, or conglomerations of such processes. In the case of the cholesterol template, for example, the variables are the mevalonate pathway, cholesterol storage, the various roles of (cholesterol-containing) plasma lipids, and so on, which are all processes altering the concentration of free or bound cholesterol. Concomitantly, the directed edges in the template model do not represent material fluxes in the traditional sense. Rather, they indicate modifying effects that may increase or decrease the activity of another template variable, which itself is a process. These effects either consist of handing molecules like cholesterol from one subsystem to another or they represent indirect modulations. Importantly, there is no material flow between template variables in a strict sense, because "Biosynthesis" does not become "Plasma Dynamics," in analogy to "Lanosterol" becoming "Cholesterol" in the anchor model. Instead, the variable "Diet" increases the amounts of cholesterol in the variables "Plasma Dynamics" and "Storage," and the variable "Biosynthesis" indirectly affects an increase in the input to the template variable "Sex Hormone Production." Furthermore, material may be generated or disappear within boxes (template variables). For instance, cholesterol disappears within the box "Sex Hormone Production," because it is converted into testosterone, estradiol, and other steroids (Figure 4). Within the template variable "Biosynthesis", cholesterol is apparently created out of nothing (Figure 4). Thus, a more accurate, although slightly awkward depiction of the cholesterol template is shown in **Figure 17**. It is of note that this representation actually runs counter to the standards of typical biological diagrams (Figure 6; [39]). For example, consider the signal emitted by a box like "Tissue Usage" on "Storage" within this diagram: It signifies that increased use of cholesterol in the peripheral tissues

indirectly affects the storage of cholesterol in the liver, for instance by triggering its release from the storage compartment or by preventing available cholesterol from being stored.

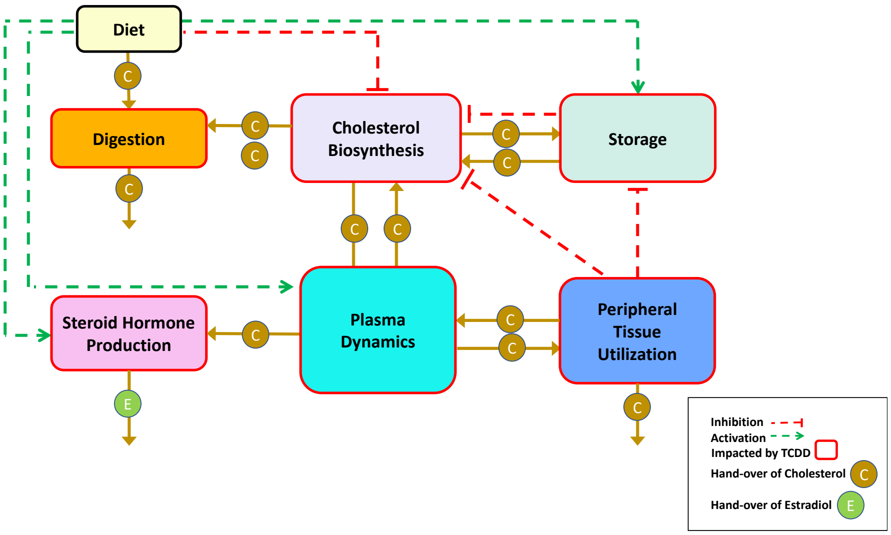

Figure 17. Cholesterol template model as dual graph. New representation of the cholesterol template model (Figure 4), where variables (anchors) are in truth processes and edges either represent the moving of cholesterol C among processes or indicate inhibitory signals S . Note that the arrows defy the standards of typical proper model diagrams (Figure 6).

The stark difference in model structures has implications for the stacking of sub-models during the zooming between templates and anchors. Specifically, it is possible that "anchors" associated with a template model can be converted into lower-level templates. This change comes with a change in model structure. As an example, "cholesterol storage in the liver" is currently an anchor model that contains as main variable the currently stored amount of cholesterol. However, if this anchor model is to become a template for an underlying model of finer granularity, the variables become the molecular and physiological processes of storing and releasing cholesterol in response to demands. The result could be a sub-template model, with its own anchors, for a lower organizational level.

Other examples of T&A-like models with processes as variables are discussed in the *Supplements*, Section S6.

4.1. From process rates to concentrations

All results of the T&A analysis (*e.g.*, Table 4) pertain to processes, as these are the variables of the template model. They do not say anything about concentrations or amounts of cholesterol in different organs or tissues. Somewhat of an analogy to this possibly perplexing aspect of the template analysis is the well-known method of flux balance analysis (FBA; [76]), which also focuses strictly on fluxes and their distribution under different conditions, while it does not shed light on concentrations. Similarly here, the template analysis reveals changes in the different processes of cholesterol handling. To obtain insight into the amounts of cholesterol, it is therefore necessary to do further model analysis.

As an example, suppose we create a model of changes in the amount of hepatic cholesterol (HC), which for this illustration is drastically simplified (**Figure 18**).

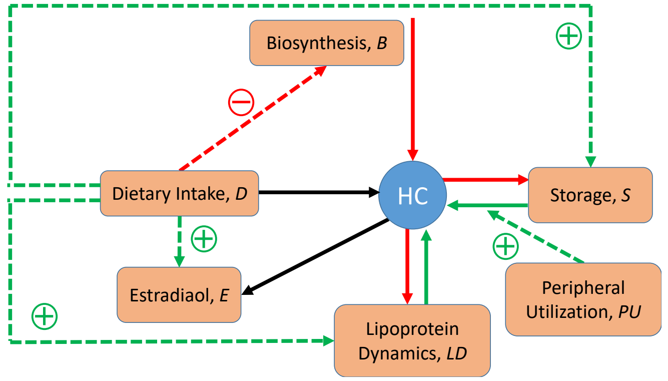

Figure 18. Utilization of flux information (*e.g.*, from Table 4) to obtain information regarding cholesterol concentrations. In this example, the concentration of interest is that of hepatic cholesterol.

The equation corresponding to the dynamics of HC, in generic terms, is:

$$\dot{HC} = k_D D + k_B B + k_{LD} LD + k_S\, fct(S, PU) - h_{LD} HC - h_S HC \qquad (8)$$

Literature information is available to set an appropriate steady-state value for HC [46], and the rates of the processes of production and consumption, if unknown, can be adjusted to reflect this value. In most cases, a mass action model will suffice, but a default alternative could be a power-law function, for instance, for the cholesterol release from storage, which involves a signal.

Given this baseline model, each of the flux rates can be modulated by simulation results associated with dioxin exposure (Table 4), which renders it possible to study the effect of dioxin on the HC concentration. Indeed, it is possible to produce a dose-response curve for the effect of dioxin on HC, which can include different amounts of cholesterol intake through the diet.

5. Discussion

The purpose of this work was to gain a deeper understanding of the organism-wide health effects of the pollutant dioxin. Dioxin is known to affect pathways, transport proteins, storage and elimination processes, and developing an efficacious model therefore has to account for different levels of biomedical organization. As a strategy for addressing this task, we re-introduce the T&A framework, which was suggested twenty-five years ago for the purpose of inferring a set of common design concepts of posture and movement from different modes of locomotion among terrestrial species [12]. The authors pursued this goal by creating a model on a conceptual low-dimensional manifold describing the key aspects of locomotion. This invariant manifold was conceived as being embedded within a much higher dimensional space describing the various observed modes of locomotion, and all species-specific implementations were seen as intersecting in the subspace of the common design [12, 13, 77].

Here, we use the T&A framework not as a method for studying design features, but as an effective tool for addressing complex tasks in biomedicine and toxicology. As such, this tool may become a potentially very powerful "New Approach Methodology" (NAM) for dissecting multiscale modeling tasks into more manageable sub-tasks. NAMs have become the target of recent research efforts in toxicology [5, 6].

We demonstrate that the T&A approach provides a systematic and intuitively organized strategy for multi-level analysis of complex biological systems. The template model serves as a high-level, coarse-grained representation that focuses on the main physiological components of a system and entails a relatively small set of variables and parameters. It reflects an overarching view of the entire problem space, thus providing an overall snapshot of the dynamic interactions among its core constituents. By contrast, the anchor models provide a more detailed and finely-grained view of specific biological details and mechanisms within the system. They allow the user to zoom into individual variables within the template, thereby permitting an account of intricate aspects of the underlying processes. Together, the template and anchor models form a hierarchical

structure, allowing for both broad overviews and in-depth analyses, thereby offering a versatile tool for multiscale modeling, while keeping computational cost at a minimum (see below).

The T&A strategy is directly applicable to the study of mechanisms with which dioxin alters the handling of cholesterol throughout the body. Thus, we designed and analyzed three detailed anchor models that were then used to inform the higher-level template model. The anchors capture the dynamics of hepatic cholesterol biosynthesis, lipoprotein transport, and the synthesis of sex hormones. The biosynthesis and lipoprotein anchors shed light on the impact of dioxin on hepatic and plasma cholesterol handling (including the roles of LDL, IDL, VLDL, and HDL cholesterol) and reveal an increase in hepatic cholesterol and a decrease in plasma cholesterol under dioxin exposure. The analysis specifically demonstrates that the upregulation of the SR-B1 and LDLR genes, which are involved in cholesterol transport between the plasma and liver, causes this decrease in plasma cholesterol concentrations and the concomitant accumulation of hepatic cholesterol. The steroidogenesis anchor demonstrates that a relatively high dose of dioxin exposure (serum concentration =10^4 ppt) leads to a reduction in estradiol synthesis, resulting in a lengthening of the menstrual cycle by nearly 3 days and consequently delaying ovulation. These findings align well with epidemiological studies [34]. Additionally, the model indicates a decrease in E2 levels with increasing doses of dioxin, which is again consistent with studies found in the literature [34].

In terms of the hierarchical structure of the T&A paradigm, the anchors correspond to the variables of the template model. Instead of carrying all details of the various anchor analyses forward, as it is attempted in many multiscale approaches, the T&A approach only uses key results from the anchors to inform the template. In our case of dioxin exposure, dose-response curves obtained with the anchors are used to modulate the variables in the template. As a result, the template offers, quasi in a minimalistic manner, a high-level overview of physiological processes and their alterations caused by external agents like dioxin. In doing so, the overarching template model captures the various processes that are explored in detail through the more fine-grained anchor models, which at this point include cholesterol biosynthesis, lipoprotein transport, peripheral cholesterol utilization, cholesterol storage, and steroid hormone production.

From a theoretical point of view, it is interesting to study the nature of variables in templates and anchors. While anchors by and large have the structure of conventional models, the variables of the template model represent processes, whereas the edges indicate how these processes in different tissues modify each other. The focus on fluxes in the template analysis is

conceptually similar to approaches like flux-balance analysis [76], whose only variables are flux rates. Importantly, FBA does not make any statements regarding metabolite concentrations. Here we show how the information from flux distributions in the template can be used for insights into concentrations, for instance, of hepatic cholesterol.

The T&A modeling approach carries distinct advantages in handling multiscale problems. In particular, the method enhances computational efficiency through its divide-and-conquer strategy and provides a flexible structure for model setup and the definition of variables across different levels. In contrast to other multiscale approaches, each anchor is separately analyzed, yielding a comprehensive record of essential input-output relationships that inform other anchors and calibrate the template model, while allowing for the interchangeability of anchor models without affecting the overall template structure.

Of course, the T&A approach is no panacea, and our dioxin exposure model, for example, is obviously simplified, like any other model. Even for a relatively straightforward anchor like the model of the mevalonate pathway, much more detail could—and maybe should—be considered, as it has been demonstrated that gene expression in the cholesterol biosynthetic pathway differs among hepatocytes depending on where they are located within the lobules, a microstructure of the liver [78-80]. The same is clearly true for other anchors, such as our model of steroidogenesis.

A possible further limitation is that we performed our analysis at the steady state of the pathway system, which may seem restrictive. However, one might expect that the pathway within the human body operates relatively close to the steady state and that the dose-response curves we determined with the anchor models are similarly applicable to non-steady-state situations. Despite these limitations, the proposed approach appears to be sufficient for revealing the essence of complex phenomena.

It goes without saying that the quality of any model, regardless of its sophistication, is fundamentally driven by the availability and accuracy of the input information used for its calibration. Furthermore, it is seldom possible to identify the mathematical structure of physiological processes directly from data. These caveats obviously pertain to our T&A analysis as well. With respect to input information, there are numerous ill-characterized uncertainties related to the values of parameters and the structure of the cholesterol handling systems. In particular, the scarcity of human data and our substitution with mouse data represent a significant

caveat. This aspect mandates caution with interpretations because, for instance, some responses of serum cholesterol levels to dioxin appear to vary between humans and mice [48, 81, 82].

To address these differences in circulating plasma cholesterol levels, while concurrently accounting for the accumulation of cholesterol in the human liver and integrating into the model additional pathways of dioxin's impact on organs besides the liver, it would be desirable to develop separate human-only or mouse-only models. Alas, the available information is currently insufficient to develop such targeted models. A critical advantage is that the models proposed here can easily be adjusted as new data become available, including the substitution of anchors with new models.

Regarding the “true” mathematical structure of the model, we resorted to the use of mass-action and power-law representations. These formulations are guaranteed by Taylor’s approximation theory to be accurate at least close to an operating point, if appropriate parameter values are used. Thus, while true functions will probably remain elusive for a very long time, the power-law-based differential equations in the model arguably constitute the most straightforward and least biased alternative (see [39] as well as references therein and above).

While certainly not complete or perfect, the T&A approach was shown to be sufficient for revealing the essence of the complex phenomenon of dioxin affecting cholesterol handling. In particular, the template model appears to perform well in capturing system-wide effects of dioxin, and as far as experimental or clinical information is available, the model results align well with reports in the literature. As an example, the template demonstrates a dose-dependent reduction in cholesterol synthesis, concurrent with a reduction in estradiol production. At the same time, cholesterol storage and utilization in peripheral tissues are increased. Furthermore, as the combined biosynthesis-plasma anchor model shows, the result of dioxin exposure is an overall increase of cholesterol in the liver, leading, in extreme cases, to non-alcoholic fatty liver disease [1].

Given the novelty of the T&A modeling paradigm, as it is proposed here, one must realize that this approach is still developing. While the T&A model appears to function properly for our application, it is obviously simplified, like any other model. Clearly, further complexity could be added to the anchor models, for instance, to account for the intricacies of cholesterol absorption from diet and the formation of bile from hepatic cholesterol, which involves a large number of enzymes and transport proteins in a multi-step process [83]. Similarly, the anchor model of

steroidogenesis examines the effects of dioxin during the follicular phase to help us understand its impact on menstrual cycle duration. Although it is noted that most variability of cycle length is usually derived from varying lengths of the follicular phase [84], the model is still woefully simplified, for example, considering that the corpus luteum also synthesizes steroid hormones similarly to the antral follicles during the follicular phase, and thus could potentially be influenced by dioxin exposure in a secondary manner. As more information becomes available, this and other anchor models may be refined, thereby enhancing their accuracy and predictive capability.

It is evident that much remains to be done, but it also becoming clear that the adaptation of the T&A approach proposed here offers substantial advantages without unduly losing resolution. The main appeal of the method is that the very complex task of multiscale analysis can be split into several, almost independent subtasks. This divide-and-conquer strategy directly enhances clarity for the model design phase, as well as computational efficiency. Unlike other methods, each anchor is analyzed individually, and while each anchor analysis generates numerous results, only key results, such as input-output relationships and their susceptibility to dioxin exposure are retained as dose-response curves or even as tabular results. Only these key results are used to inform the template model. One might add that anchor models can be replaced with alternative representations without affecting the structure of the template or other anchors. Finally, the T&A approach provides flexible guidelines for the setup of the two model types and for defining variables and interactions across scales. These guidelines may pertain to average models for populations or may be applied to customized, individually personalized models in straightforward extensions [85-87], These personalized models could, in turn, become the basis for risk assessments and virtual clinical trials [88, 89].

Multiscale approaches are clearly important for biomedical modeling, and the T&A paradigm provides a novel, effective tool for complex modeling tasks as it provides a comprehensive and holistic perspective that accommodates a range of organizational scales. Furthermore, an approach like T&A can readily be tailored toward many different research goals and applications, which could, for instance, support traditionally difficult toxicological extrapolations of *in vitro* to *in vivo* results [6] and the important translation of observations between different species [90]. For our example of dioxin exposure, this translation poses an interesting challenge, as some enzyme activities and the crucial dioxin-AhR mechanism are species-specific [91, 92].

As one future application, the T&A framework holds potential for advancing personalized risk assessments and personalized medicine [85-87] in general. It could also become relevant and effective for enhancing drug discovery and development. The current version of the model is mostly calibrated with average data, but we also indicate how variability may be taken into account. We illustrate this aspect with the example of inter-individual variability in menstrual cycle length within a population, where the production of estradiol is affected by dioxin. For future uses in personalized medicine, the model design is readily customizable, according to published personalization methods [85-87, 93, 94] that are based on individualized adjustments of relevant parameters and thus on personal data. These examples render it evident that the T&A platform is versatile and can be expanded in various directions to simulate the body's response to a wide array of pharmaceutical and toxic compounds, thereby potentially serving as a predictive tool for assessing the behaviors and interactions of drugs and environmental exposures within the body [95].

In conclusion, the T&A framework holds promise for multiscale modeling, for a deeper understanding of complex systems, and for advancing personalized medicine and risk assessment, interspecies translation [90], and drug testing through virtual clinical trials [96, 97].

Code Availability

Modeling details and computer code can be found in the supplements and on GitHub.com/LBSA-VoitLab/TCDD_Chol. Submission to Biomodels will follow after acceptance of the article.

Author Contributions

Conceptualization: Carla M. Kumbale, Qiang Zhang, Eberhard O. Voit
Data Curation: Carla M. Kumbale
Formal Analysis: Carla M. Kumbale, Qiang Zhang, Eberhard O. Voit
Funding Acquisition: Qiang Zhang, Eberhard O. Voit
Investigation: Carla M. Kumbale, Qiang Zhang, Eberhard O. Voit
Methodology: Carla M. Kumbale, Qiang Zhang, Eberhard O. Voit
Project Administration: Eberhard O. Voit
Resources: N/A
Software: Carla M. Kumbale, Qiang Zhang
Validation: Carla M. Kumbale
Visualization: Carla M. Kumbale, Qiang Zhang, Eberhard O. Voit
Writing (original draft): Carla M. Kumbale, Qiang Zhang, Eberhard O. Voit
Writing (review and editing): Carla M. Kumbale, Qiang Zhang, Eberhard O. Voit

Acknowledgments

The authors would like to thank Dr. Shuo Xiao for helpful technical discussions regarding steroidogenesis and the menstrual cycle.

Financial Disclosure
This work was supported by the National Institute of Environmental Health Sciences (HERCULES grant P30ES019776-05 to Carmen Marsit and Superfund Research grant P42ES04911 to Norbert Kaminski). The funders had no role in study design, data collection and analysis, decision to publish, or preparation of the manuscript.

Supporting Information
The Supplements contain the following information:
S1. Design of a Template-and-Anchor (T&A) Model
S2. Model Equations and Parameter Values
S3. Simulating Dioxin Exposure in PLAS
S4. Simulating Dioxin Exposure and Diet in PLAS
S5. Sensitivity Analysis
S6. Examples of T&A-like models with processes as variables

6. References

1. Kumbale, C.M., Q. Zhang, and E.O. Voit, *Hepatic cholesterol biosynthesis and dioxin-induced dysregulation: A multiscale computational approach.* Food and Chemical Toxicology, 2023: p. 114086.
2. Lundbäck, M. *Dioxins*. 2023; Available from: https://www.ucsfhealth.org/education/cholesterol-content-of-foods.
3. Poland, A. and A. Kende, *2,3,7,8-Tetrachlorodibenzo-p-dioxin: environmental contaminant and molecular probe.* Fed Proc, 1976. **35**(12): p. 2404-11.
4. Gao, J., et al., *A review of food contaminant 2,3,7,8-tetrachlorodibenzo-p-dioxin and its toxicity associated with metabolic disorders.* Curr Res Food Sci, 2023. **7**: p. 100617.
5. Sewell, F., et al., *New approach methodologies (NAMs): identifying and overcoming hurdles to accelerated adoption.* Toxicol Res (Camb), 2024. **13**(2): p. tfae044.
6. Zhang, Q., et al., *Bridging the Data Gap From in vitro Toxicity Testing to Chemical Safety Assessment Through Computational Modeling.* Front Public Health, 2018. **6**: p. 261.
7. Schnell, S., R. Grima, and P. Maini, *Multiscale Modeling in Biology.* American Scientist - AMER SCI, 2007. **95**.
8. Weinan, E. and B. Engquist, *Multiscale Modeling and Computation.* Notices of the American Mathematical Society, 2011. **50**.
9. Versypt, A.N.F., *Multiscale modeling in disease.* Curr Opin Syst Biol, 2021. **27**.
10. Fletcher, A.G. and J.M. Osborne, *Seven challenges in the multiscale modeling of multicellular tissues.* WIREs Mech Dis, 2022. **14**(1): p. e1527.
11. Millar-Wilson, A., et al., *Multiscale modeling in the framework of biological systems and its potential for spaceflight biology studies.* iScience, 2022. **25**(11): p. 105421.
12. Full, R.J. and D.E. Koditschek, *Templates and anchors: neuromechanical hypotheses of legged locomotion on land.* J Exp Biol, 1999. **202**(Pt 23): p. 3325-32.
13. Kvalheim, M., and Revzen, S, *Templates and Anchors; in M.A.* Bioinspired Legged Locomotion. Models, Concepts, Control and Applications, ed. S.a.A. Seyfarth. 2017, Oxford, UK.
14. Davis, A.P., et al., *Comparative Toxicogenomics Database: a knowledgebase and discovery tool for chemical-gene-disease networks.* Nucleic Acids Res, 2009. **37**(Database issue): p. D786-92.

15. Greene, J.F., S. Hays, and D. Paustenbach, *Basis for a proposed reference dose (RfD) for dioxin of 1-10 pg/kg-day: a weight of evidence evaluation of the human and animal studies.* J Toxicol Environ Health B Crit Rev, 2003. **6**(2): p. 115-59.
16. Feingold, K.R., *Introduction to Lipids and Lipoproteins*, in *Endotext*, K.R. Feingold, et al., Editors. 2000: South Dartmouth (MA).
17. Kanat, M., et al., *Is lipid lowering treatment aiming for very low LDL levels safe in terms of the synthesis of steroid hormones?* Med Hypotheses, 2007. **69**(1): p. 104-12.
18. van de Sluis, B., M. Wijers, and J. Herz, *News on the molecular regulation and function of hepatic low-density lipoprotein receptor and LDLR-related protein 1.* Curr Opin Lipidol, 2017. **28**(3): p. 241-247.
19. Czarnecka, H. and S. Yokoyama, *Regulation of cellular cholesterol efflux by lecithin:cholesterol acyltransferase reaction through nonspecific lipid exchange.* J Biol Chem, 1996. **271**(4): p. 2023-8.
20. Ouimet, M., T.J. Barrett, and E.A. Fisher, *HDL and Reverse Cholesterol Transport.* Circ Res, 2019. **124**(10): p. 1505-1518.
21. Shen, W.J., S. Azhar, and F.B. Kraemer, *SR-B1: A Unique Multifunctional Receptor for Cholesterol Influx and Efflux.* Annu Rev Physiol, 2018. **80**: p. 95-116.
22. Hillier, S.G., P.F. Whitelaw, and C.D. Smyth, *Follicular oestrogen synthesis: the 'two-cell, two-gonadotrophin' model revisited.* Mol Cell Endocrinol, 1994. **100**(1-2): p. 51-4.
23. Christenson, L.K. and L. Devoto, *Cholesterol transport and steroidogenesis by the corpus luteum.* Reprod Biol Endocrinol, 2003. **1**: p. 90.
24. Lavoie, H.A. and S.R. King, *Transcriptional regulation of steroidogenic genes: STARD1, CYP11A1 and HSD3B.* Exp Biol Med (Maywood), 2009. **234**(8): p. 880-907.
25. Stocco, D.M. and B.J. Clark, *Role of the steroidogenic acute regulatory protein (StAR) in steroidogenesis.* Biochem Pharmacol, 1996. **51**(3): p. 197-205.
26. Manna, P.R., et al., *Role of the steroidogenic acute regulatory protein in health and disease.* Endocrine, 2016. **51**(1): p. 7-21.
27. Yonemoto, J., *The effects of dioxin on reproduction and development.* Ind Health, 2000. **38**(3): p. 259-68.
28. Barsotti, D.A., L.J. Abrahamson, and J.R. Allen, *Hormonal alterations in female rhesus monkeys fed a diet containing 2, 3,7,8-tetrachlorodibenzo-p-dioxin.* Bull Environ Contam Toxicol, 1979. **21**(4-5): p. 463-9.
29. Valdez, K.E., et al., *Effect of chronic exposure to the aryl hydrocarbon receptor agonist 2,3,7,8-tetrachlorodibenzo-p-dioxin in female rats on ovarian gene expression.* Reprod Toxicol, 2009. **28**(1): p. 32-7.
30. Enan, E., et al., *Mechanism of toxic action of 2,3,7,8-tetrachlorodibenzo-p-dioxin (tcdd) in cultured human luteinized granulosa cells.* Reproductive Toxicology, 1996. **10**(6): p. 497-508.
31. Karman, B.N., et al., *2,3,7,8-Tetrachlorodibenzo-p-dioxin activates the aryl hydrocarbon receptor and alters sex steroid hormone secretion without affecting growth of mouse antral follicles in vitro.* Toxicol Appl Pharmacol, 2012. **261**(1): p. 88-96.
32. Karman, B.N., et al., *Dioxin exposure reduces the steroidogenic capacity of mouse antral follicles mainly at the level of HSD17B1 without altering atresia.* Toxicol Appl Pharmacol, 2012. **264**(1): p. 1-12.

33. Li, X., D.C. Johnson, and K.K. Rozman, *Effects of 2,3,7,8-tetrachlorodibenzo-p-dioxin (TCDD) on estrous cyclicity and ovulation in female Sprague-Dawley rats.* Toxicol Lett, 1995. **78**(3): p. 219-22.
34. Eskenazi, B., et al., *Serum dioxin concentrations and menstrual cycle characteristics.* Am J Epidemiol, 2002. **156**(4): p. 383-92.
35. Buck Louis, G.M., et al., *Persistent organochlorine pollutants and menstrual cycle characteristics.* Chemosphere, 2011. **85**(11): p. 1742-8.
36. Morán, F.M., et al., *2,3,7,8-tetrachlorodibenzo-p-dioxin decreases estradiol production without altering the enzyme activity of cytochrome P450 aromatase of human luteinized granulosa cells in vitro.* Biol Reprod, 2000. **62**(4): p. 1102-8.
37. Morán, F.M., et al., *Molecular target of endocrine disruption in human luteinizing granulosa cells by 2,3,7,8-tetrachlorodibenzo-p-dioxin: inhibition of estradiol secretion due to decreased 17alpha-hydroxylase/17,20-lyase cytochrome P450 expression.* Endocrinology, 2003. **144**(2): p. 467-73.
38. Dasmahapatra, A.K., et al., *Demonstration of 2,3,7,8-tetrachlorodibenzo-p-dioxin attenuation of P450 steroidogenic enzyme mRNAs in rat granulosa cell in vitro by competitive reverse transcriptase-polymerase chain reaction assay.* Mol Cell Endocrinol, 2000. **164**(1-2): p. 5-18.
39. Voit, E.O., *The best models of metabolism.* Wiley Interdiscip Rev Syst Biol Med, 2017. **9**(6).
40. Savageau, M.A., *Biochemical Systems Analysis: A Study of Function and Design in Molecular Biology*. 1976.
41. Voit, E.O., *Biochemical Systems Theory: A Review.* ISRN Biomathematics, 2013. **2013**: p. 897658.
42. Ogata, K., *Modern Control Engineering (2nd Edition)*. 1990, Englewood Cliffs, NJ: Prentice Hall.
43. Voit, E., *A First Course in Systems Biology (2nd ed.). Garland Science*. 2017.
44. Voit, E.O., H.A. Martens, and S.W. Omholt, *150 years of the mass action law.* PLoS Comput Biol, 2015. **11**(1): p. e1004012.
45. Voit, E.O., *Computational Analysis of Biochemical Systems: A Practical Guide for Biochemists and Molecular Biologists*. 2000, Cambridge, U.K.: Cambridge University Press.
46. Sniderman, A.D., et al., *Hepatic cholesterol homeostasis: is the low-density lipoprotein pathway a regulatory or a shunt pathway?* Arterioscler Thromb Vasc Biol, 2013. **33**(11): p. 2481-90.
47. Lee, C.C., et al., *Fatty liver and hepatic function for residents with markedly high serum PCDD/Fs levels in Taiwan.* J Toxicol Environ Health A, 2006. **69**(5): p. 367-80.
48. Angrish, M.M., C.Y. Dominici, and T.R. Zacharewski, *TCDD-elicited effects on liver, serum, and adipose lipid composition in C57BL/6 mice.* Toxicol Sci, 2013. **131**(1): p. 108-15.
49. Stricker, R., et al., *Establishment of detailed reference values for luteinizing hormone, follicle stimulating hormone, estradiol, and progesterone during different phases of the menstrual cycle on the Abbott ARCHITECT analyzer.* Clin Chem Lab Med, 2006. **44**(7): p. 883-7.
50. Groome, N.P., et al., *Measurement of dimeric inhibin B throughout the human menstrual cycle.* J Clin Endocrinol Metab, 1996. **81**(4): p. 1401-5.

51. Hutz, R.J., et al., *Environmental toxicants and effects on female reproductive function.* Tren Reprod Bio, 2006. **2**: p. 1-11.
52. Carson, J.A.S., et al., *Dietary Cholesterol and Cardiovascular Risk: A Science Advisory From the American Heart Association.* Circulation, 2020. **141**(3): p. e39-e53.
53. Griffin, J.D. and A.H. Lichtenstein, *Dietary Cholesterol and Plasma Lipoprotein Profiles: Randomized-Controlled Trials.* Curr Nutr Rep, 2013. **2**(4): p. 274-282.
54. Harlow, S.D., X. Lin, and M.J. Ho, *Analysis of menstrual diary data across the reproductive life span Applicability of the bipartite model approach and the importance of within-woman variance.* Journal of Clinical Epidemiology, 2000. **53**(7): p. 722-733.
55. Treloar, A.E., et al., *Variation of the human menstrual cycle through reproductive life.* Int J Fertil, 1967. **12**(1 Pt 2): p. 77-126.
56. Bull, J.R., et al., *Real-world menstrual cycle characteristics of more than 600,000 menstrual cycles.* npj Digital Medicine, 2019. **2**(1): p. 83.
57. Grieger, J.A. and R.J. Norman, *Menstrual Cycle Length and Patterns in a Global Cohort of Women Using a Mobile Phone App: Retrospective Cohort Study.* J Med Internet Res, 2020. **22**(6): p. e17109.
58. Fehring, R.J., M. Schneider, and K. Raviele, *Variability in the phases of the menstrual cycle.* J Obstet Gynecol Neonatal Nurs, 2006. **35**(3): p. 376-84.
59. Lakshman, M.R., et al., *Effects of 2,3,7,8-tetrachlorodibenzo-p-dioxin (TCDD) on de novo fatty acid and cholesterol synthesis in the rat.* Lipids, 1988. **23**(9): p. 904-6.
60. Lind, P.M., et al., *The dioxin-like pollutant PCB 126 (3,3',4,4',5-pentachlorobiphenyl) affects risk factors for cardiovascular disease in female rats.* Toxicology Letters, 2004. **150**(3): p. 293-299.
61. Humblet, O., et al., *Dioxins and cardiovascular disease mortality.* Environ Health Perspect, 2008. **116**(11): p. 1443-8.
62. Varbo, A., M. Benn, and B.G. Nordestgaard, *Remnant cholesterol as a cause of ischemic heart disease: Evidence, definition, measurement, atherogenicity, high risk patients, and present and future treatment.* Pharmacology & Therapeutics, 2014. **141**(3): p. 358-367.
63. Moslehi, A. and Z. Hamidi-Zad, *Role of SREBPs in Liver Diseases: A Mini-review.* J Clin Transl Hepatol, 2018. **6**(3): p. 332-338.
64. Dietschy, J.M., *Dietary Fatty Acids and the Regulation of Plasma Low Density Lipoprotein Cholesterol Concentrations1,2.* The Journal of Nutrition, 1998. **128**(2): p. 444S-448S.
65. Mc Auley, M.T., et al., *A whole-body mathematical model of cholesterol metabolism and its age-associated dysregulation.* BMC Syst Biol, 2012. **6**: p. 130.
66. Barona, J. and M.L. Fernandez, *Dietary cholesterol affects plasma lipid levels, the intravascular processing of lipoproteins and reverse cholesterol transport without increasing the risk for heart disease.* Nutrients, 2012. **4**(8): p. 1015-25.
67. Kostner, K.M., *Understanding Cholesterol Synthesis and Absorption Is the Key to Achieving Cholesterol Targets.* Asia-Pacific Cardiology 2007;1(1):7-10, 2007.
68. Malhotra, P., et al., *Disturbances in Cholesterol Homeostasis and Non-alcoholic Fatty Liver Diseases.* Front Med (Lausanne), 2020. **7**: p. 467.
69. Whitney, H., *A logical expansion in mathematics.* Bulletin of the American Mathematical Society, 1932. **38**(8): p. 572-579, 8.
70. Harary, F. and R.Z. Norman, *Some properties of line digraphs.* Rendiconti del Circolo Matematico di Palermo, 1960. **9**(2): p. 161-168.

71. Beineke, L.W. and R.L. Hemminger, *Infinite digraphs isomorphic with their line digraphs.* Journal of Combinatorial Theory, Series B, 1976. **21**(3): p. 245-256.
72. Dassios, I. and F. Milano, *Singular dual systems of fractional-order differential equations.* Mathematical Methods in the Applied Sciences, 2021.
73. Rudolph, J., *Duality in time-varying linear systems: A module theoretic approach.* Linear Algebra and its Applications, 1996. **245**: p. 83-106.
74. Bruce, J.W. and F. Tari, *Duality and implicit differential equations.* Nonlinearity, 2000. **13**(3): p. 791.
75. Marin-Sanguino, A., E.R. Mendoza, and E.O. Voit, *Flux duality in nonlinear GMA systems: implications for metabolic engineering.* J Biotechnol, 2010. **149**(3): p. 166-72.
76. Orth, J.D., I. Thiele, and B.Ø. Palsson, *What is flux balance analysis?* Nature Biotechnology, 2010. **28**(3): p. 245-248.
77. Holmes, P., et al., *The Dynamics of Legged Locomotion: Models, Analyses, and Challenges.* SIAM Review, 2006. **48**(2): p. 207-304.
78. Fader, K.A., et al., *2,3,7,8-Tetrachlorodibenzo-p-Dioxin Alters Lipid Metabolism and Depletes Immune Cell Populations in the Jejunum of C57BL/6 Mice.* Toxicol Sci, 2015. **148**(2): p. 567-80.
79. Nault, R., et al., *Dose-Dependent Metabolic Reprogramming and Differential Gene Expression in TCDD-Elicited Hepatic Fibrosis.* Toxicol Sci, 2016. **154**(2): p. 253-266.
80. Nault, R., et al., *Lipidomic Evaluation of Aryl Hydrocarbon Receptor-Mediated Hepatic Steatosis in Male and Female Mice Elicited by 2,3,7,8-Tetrachlorodibenzo-p-dioxin.* Chem Res Toxicol, 2017. **30**(4): p. 1060-1075.
81. Martin, J.V., *Lipid abnormalities in workers exposed to dioxin.* Br J Ind Med, 1984. **41**(2): p. 254-256.
82. Gasiewicz, T.A., et al., *Distribution, excretion, and metabolism of 2,3,7,8-tetrachlorodibenzo-p-dioxin in C57BL/6J, DBA/2J, and B6D2F1/J mice.* Drug Metab Dispos, 1983. **11**(5): p. 397-403.
83. Hernell, O., J.E. Staggers, and M.C. Carey, *Physical-chemical behavior of dietary and biliary lipids during intestinal digestion and absorption. 2. Phase analysis and aggregation states of luminal lipids during duodenal fat digestion in healthy adult human beings.* Biochemistry, 1990. **29**(8): p. 2041-56.
84. Reed, B.G. and B.R. Carr, *The Normal Menstrual Cycle and the Control of Ovulation*, in *Endotext*, K.R. Feingold, et al., Editors. 2000: South Dartmouth (MA).
85. Davis, J.D., et al., *Dynamical systems approaches to personalized medicine.* Curr Opin Biotechnol, 2019. **58**: p. 168-174.
86. Voit, E.O. and K.L. Brigham, *The Role of Systems Biology in Predictive Health and Personalized Medicine.* The Open Pathology Journal,, 2008. **2**: p. 68-70.
87. Kumbale, C.M. and E.O. Voit, *Toward Personalized Medicine for HIV/AIDS.* J AIDS HIV Treat, 2021. **3**(2): p. 37-41.
88. Narayanasetty, S. and D. Jallu, *A Review on Virtual Clinical Trials: The Future.* International Journal of Pharmaceutical Sciences Review and Research, 2021. **68**.
89. Ng, C.E., et al., *The future of clinical trials-is it virtual?* Br Med Bull, 2023. **148**(1): p. 42-57.
90. Brubaker, D.K., et al., *An interspecies translation model implicates integrin signaling in infliximab-resistant inflammatory bowel disease.* Sci Signal, 2020. **13**(643).

91. Schulz, T.G., et al., *Inducibility of cytochromes P-450 by dioxin in liver and extrahepatic tissues of the marmoset monkey (Callithrix jacchus).* Biochimica et Biophysica Acta (BBA) - Protein Structure and Molecular Enzymology, 1996. **1298**(1): p. 131-140.
92. Fader, K.A. and T.R. Zacharewski, *Beyond the Aryl Hydrocarbon Receptor: Pathway Interactions in the Hepatotoxicity of 2,3,7,8-Tetrachlorodibenzo-p-dioxin and Related Compounds.* Curr Opin Toxicol, 2017. **2**: p. 36-41.
93. Chen, R. and M. Snyder, *Systems biology: personalized medicine for the future?* Curr Opin Pharmacol, 2012. **12**(5): p. 623-8.
94. Deisboeck, T.S., *Personalizing medicine: a systems biology perspective.* Molecular Systems Biology, 2009. **5**(1).
95. Schoeberl, B., *Quantitative Systems Pharmacology models as a key to translational medicine.* Current Opinion in Systems Biology, 2019. **16**: p. 25-31.
96. Ranganathan, P., R. Aggarwal, and C.S. Pramesh, *Virtual clinical trials.* Persp. Clin. Res., 2023. **14**(4): p. 203-206.
97. Shore C, Khandekar E, and A.J. (Eds), *Virtual Clinical Trials: Challenges and Opportunities: Proceedings of a Workshop.* National Academies Press (US), Available from: https://www.ncbi.nlm.nih.gov/books/NBK548981/, 2019. **July 23 (1)**.

Figure 1 Click here to access/download;Figure;Figure 1 .tif

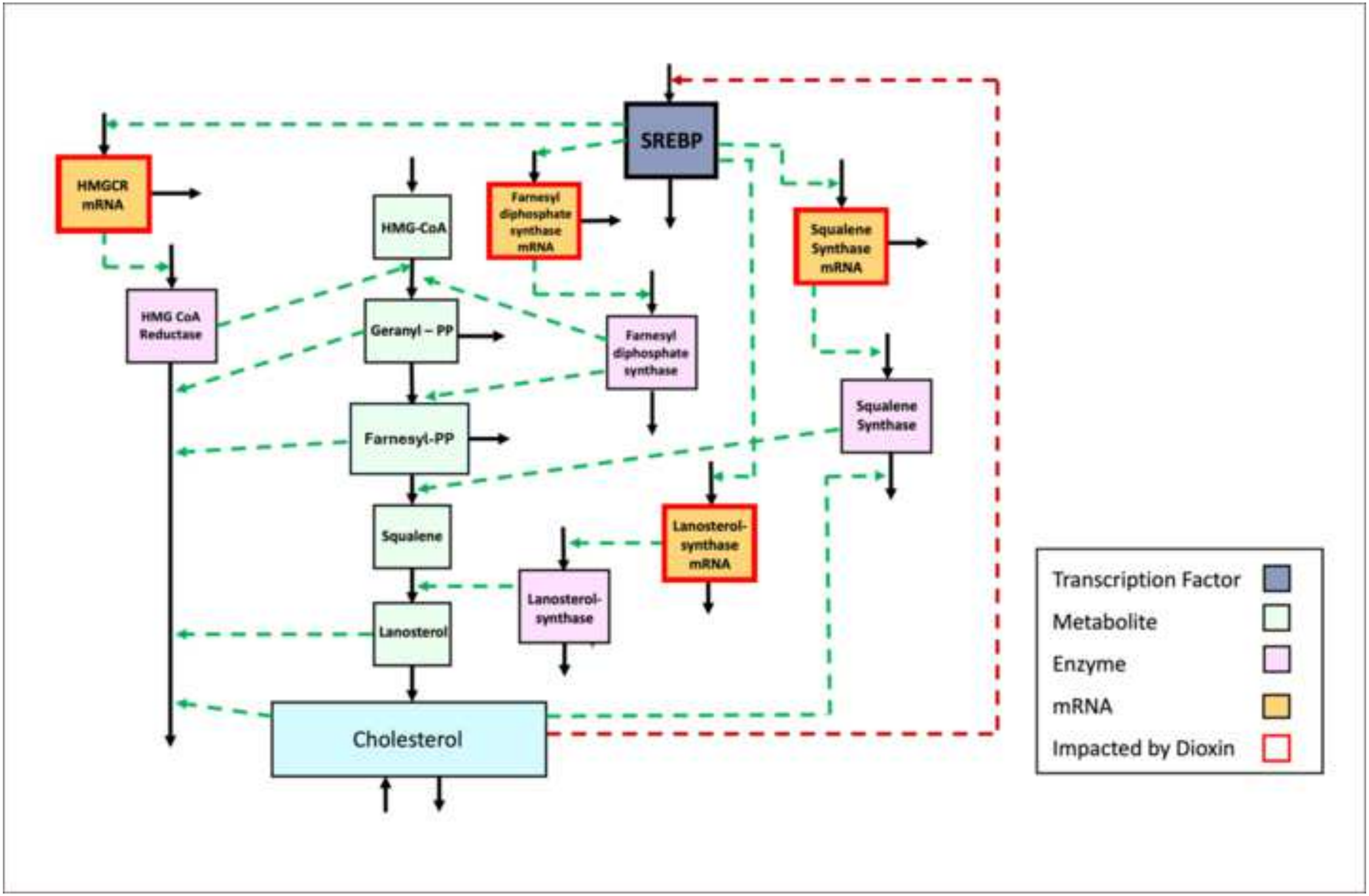

Figure 2

Click here to access/download;Figure;Figure 2.tif

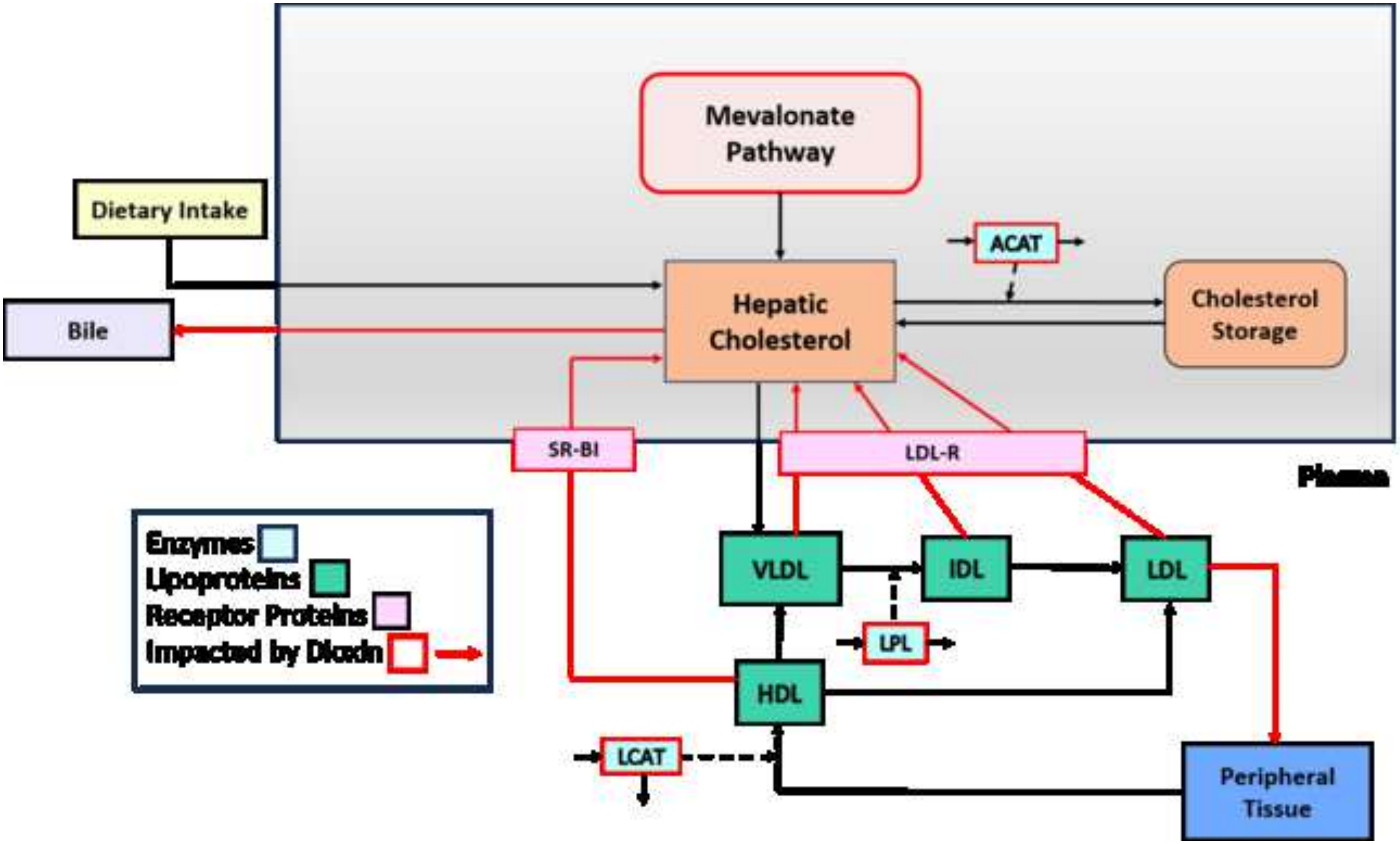

Figure 3

Click here to access/download;Figure;Figure 3.tif

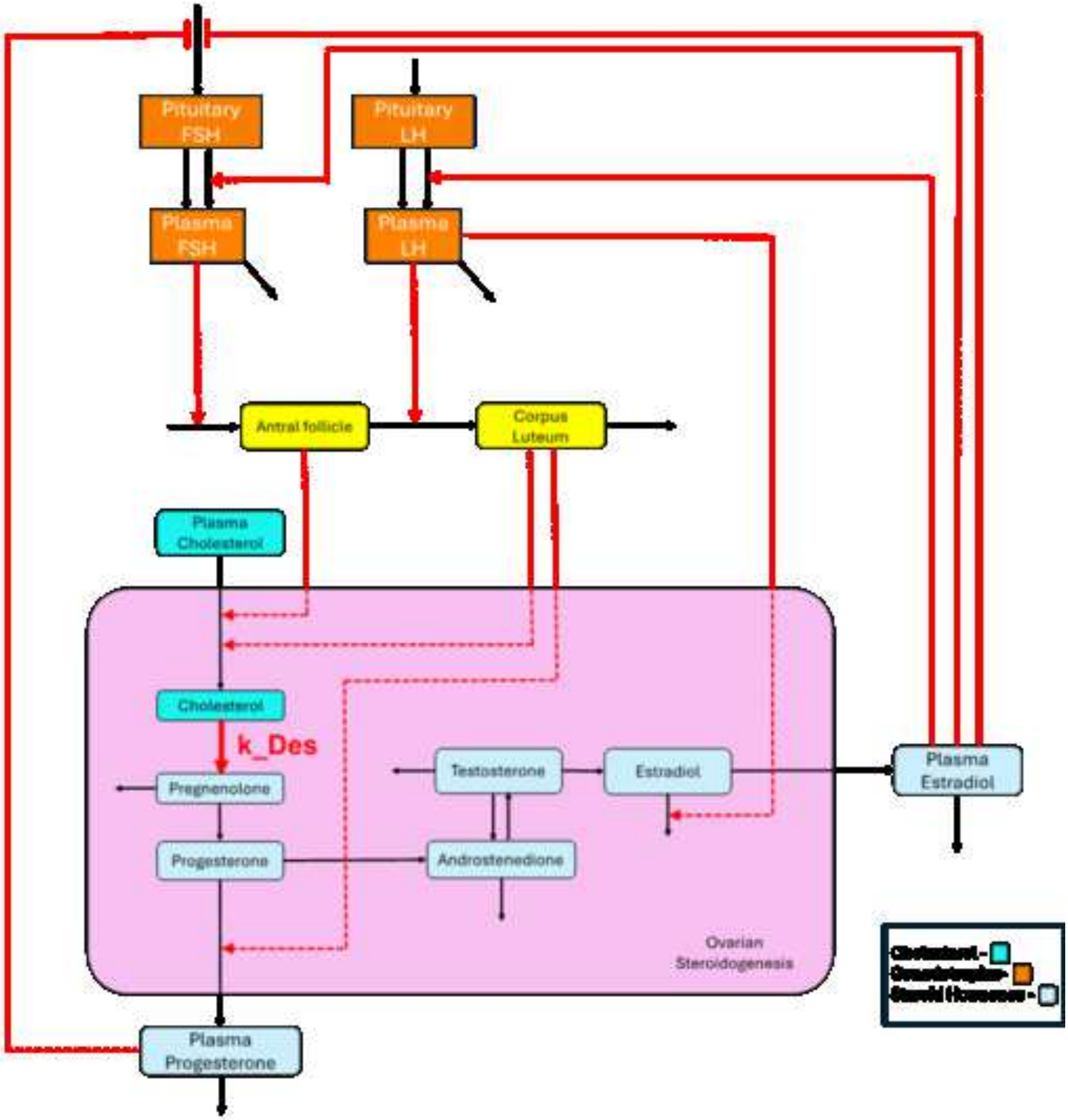

Figure 4

Click here to access/download;Figure;Figure 4.tif

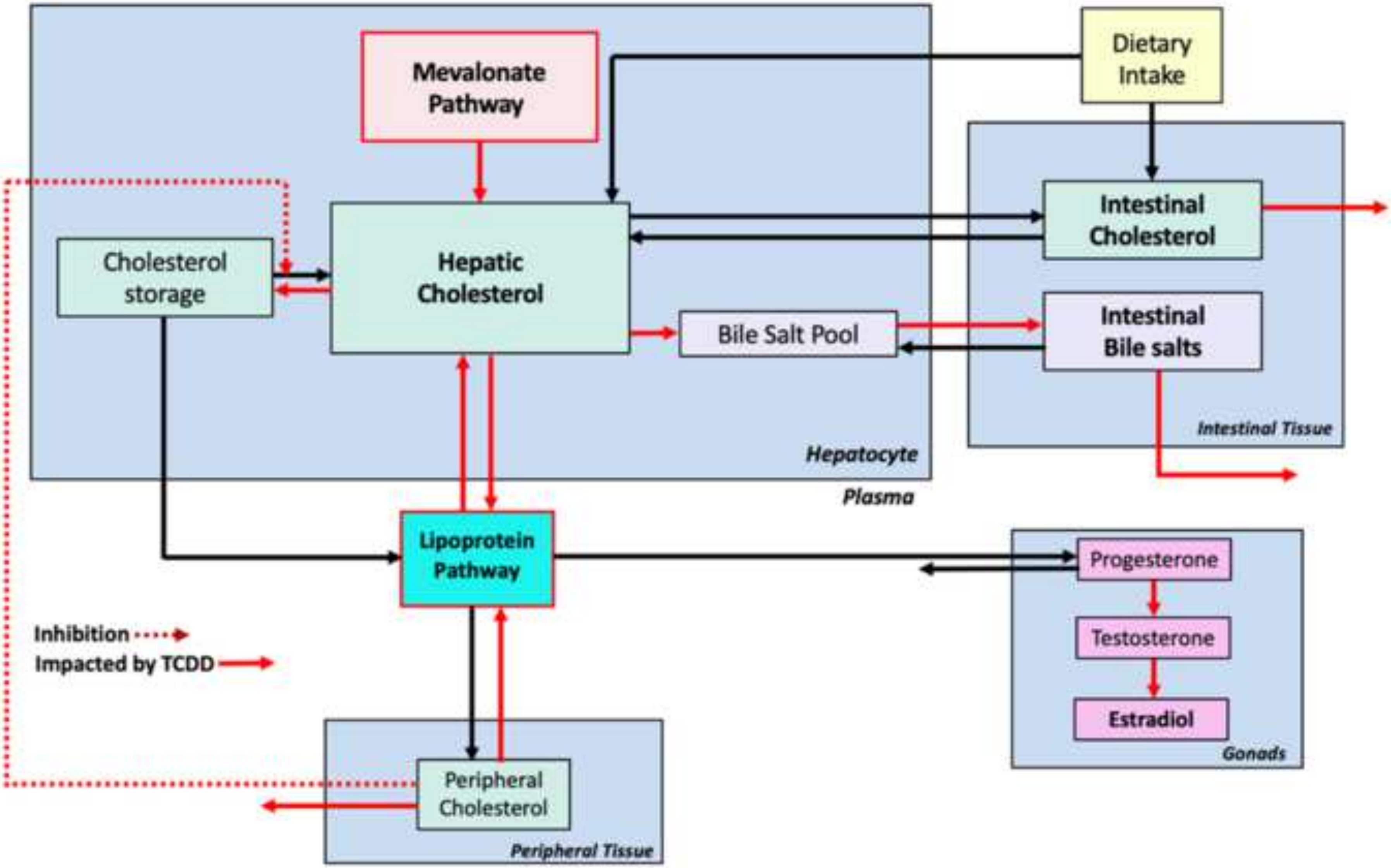

Figure 5

Click here to access/download;Figure;Figure 5.tif

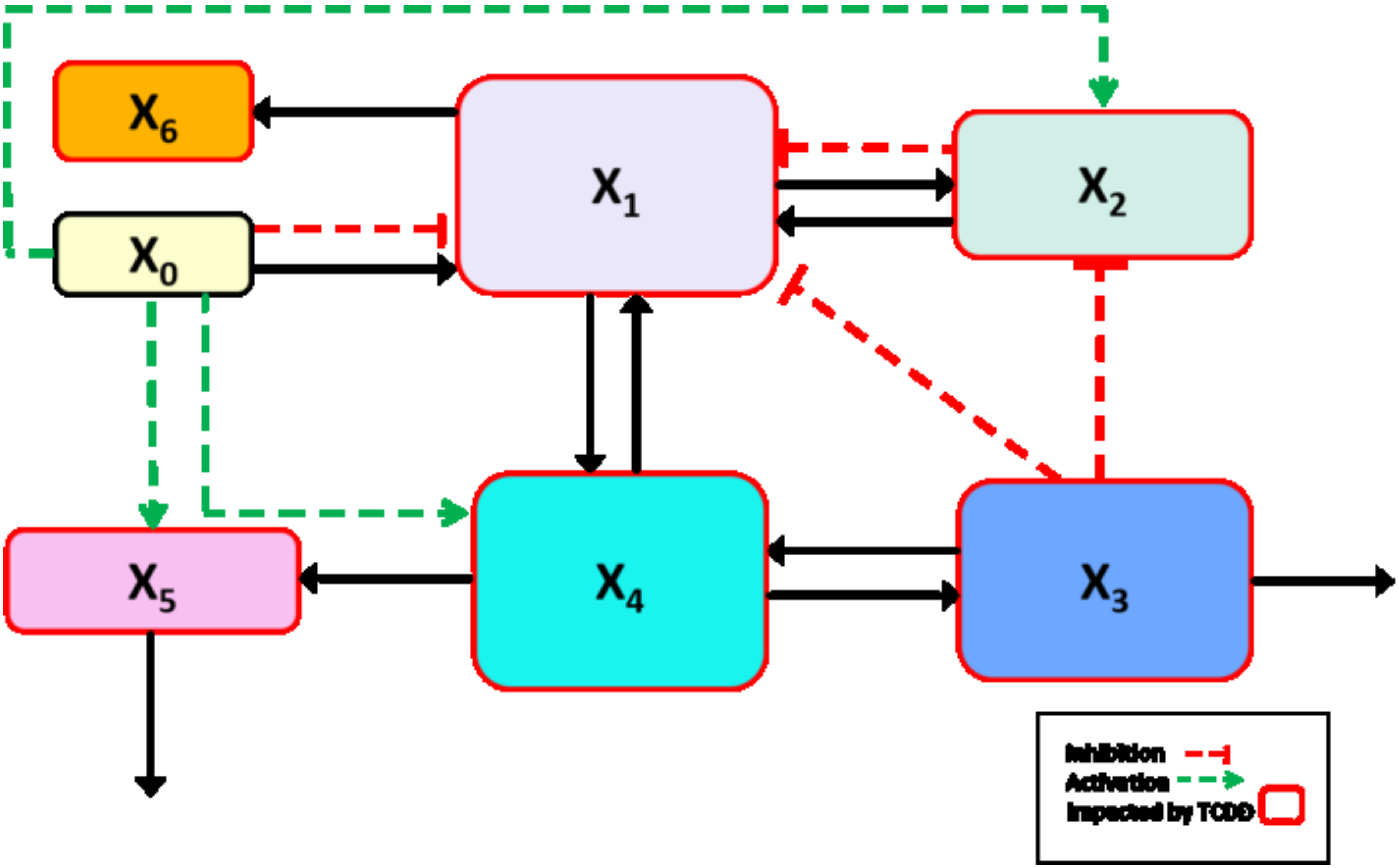

Figure 6

Click here to access/download;Figure;Figure 6.tif

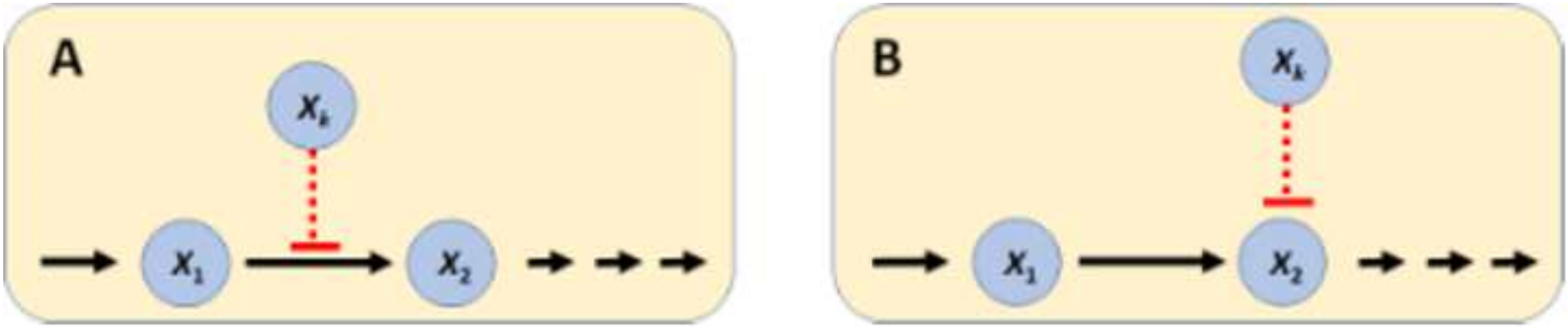

Figure 7

Click here to access/download;Figure;Figure 7.tif

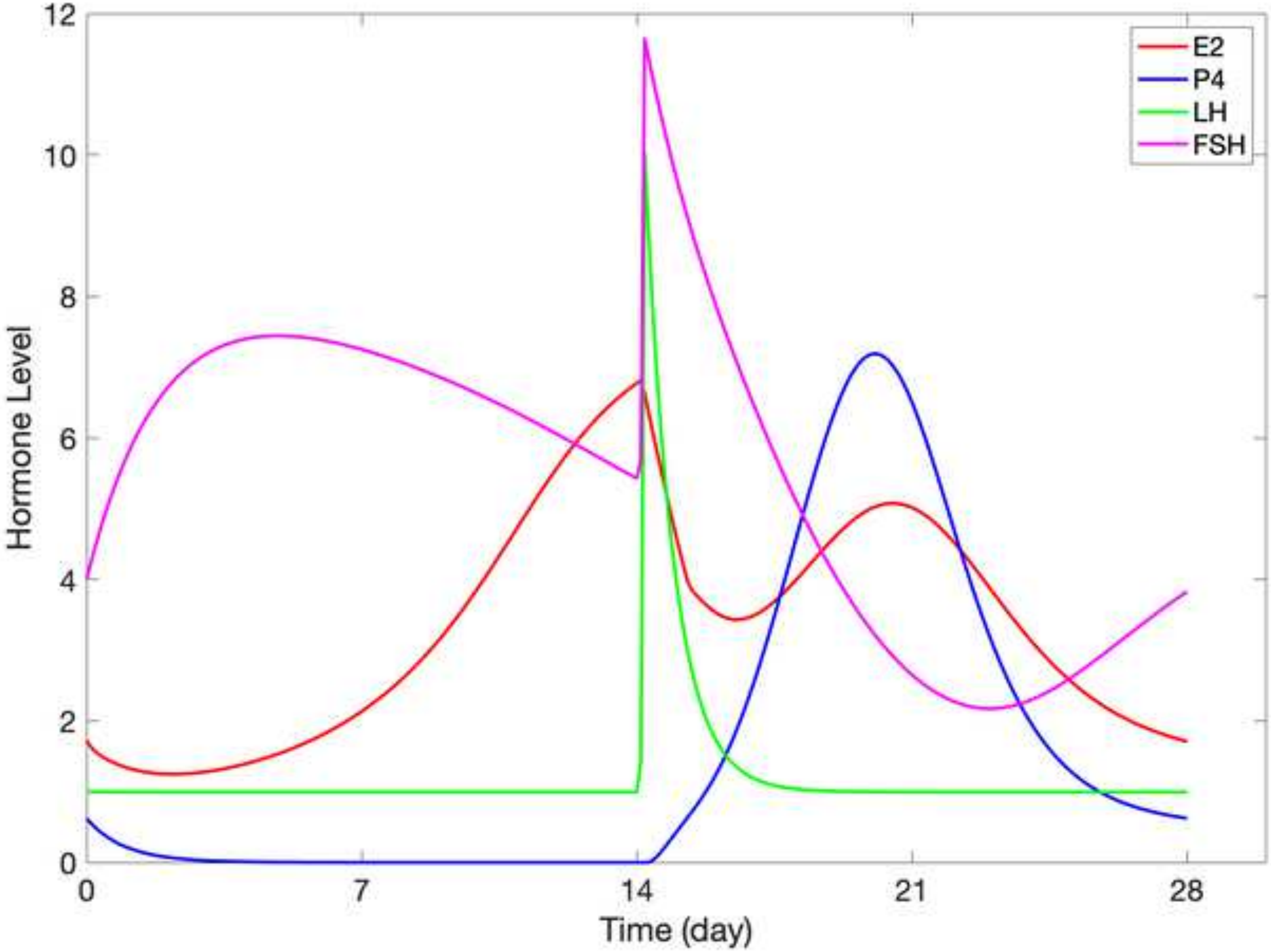

Figure 8

Click here to access/download;Figure;Figure 8.tif

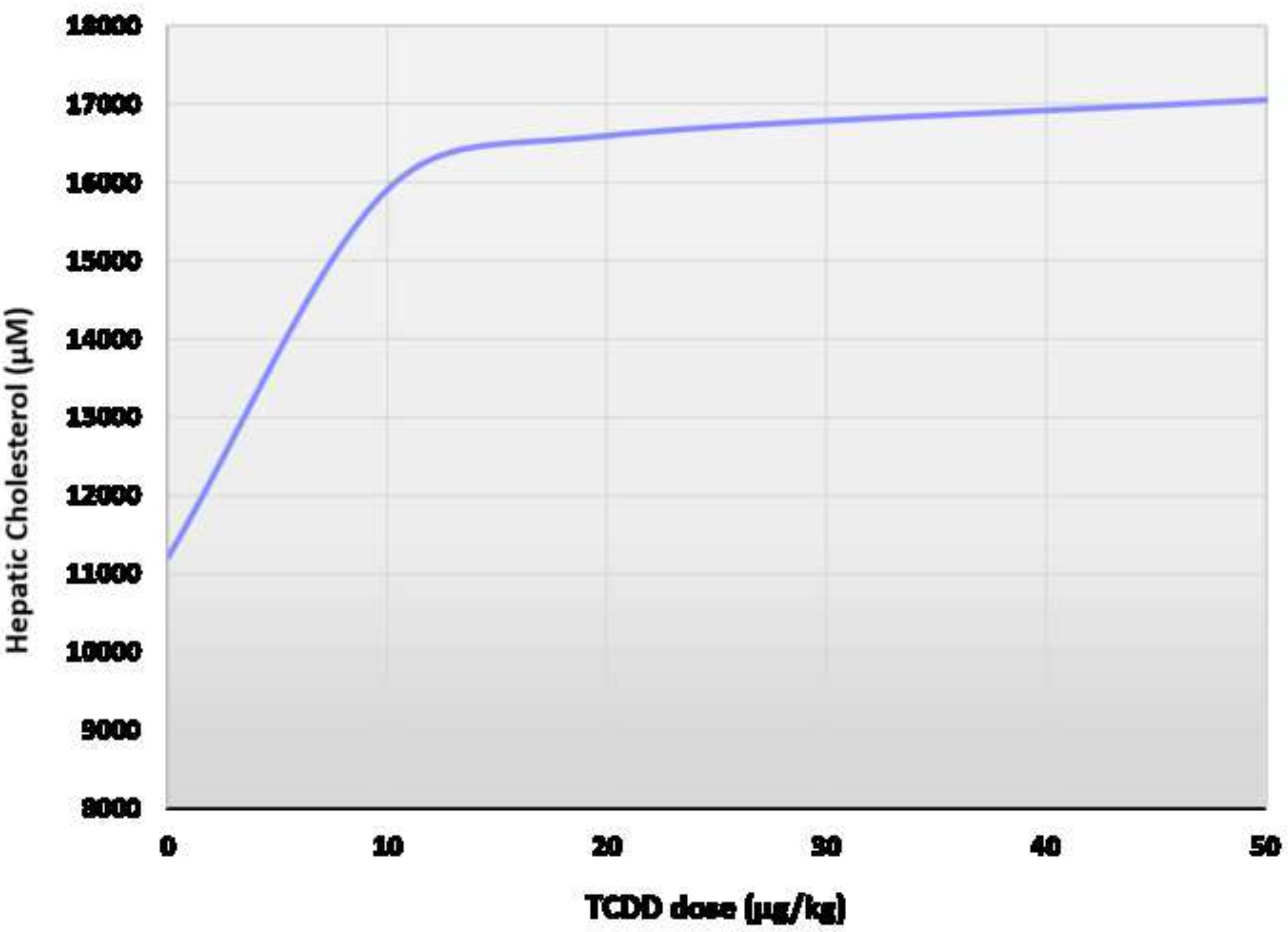

Figure 9

Click here to access/download;Figure;Figure 9.tif

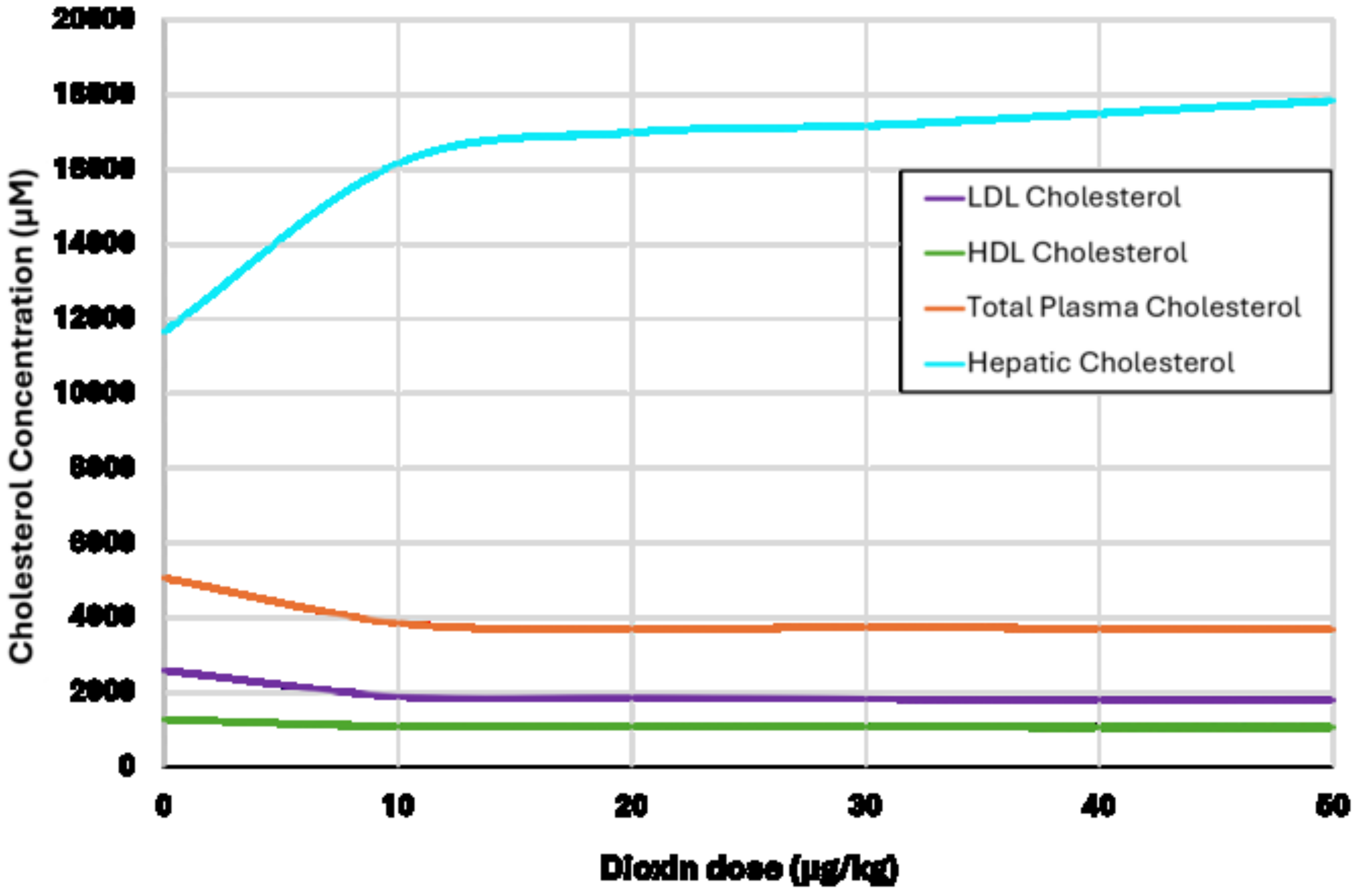

Figure 10

Click here to access/download;Figure;Figure 10.tif

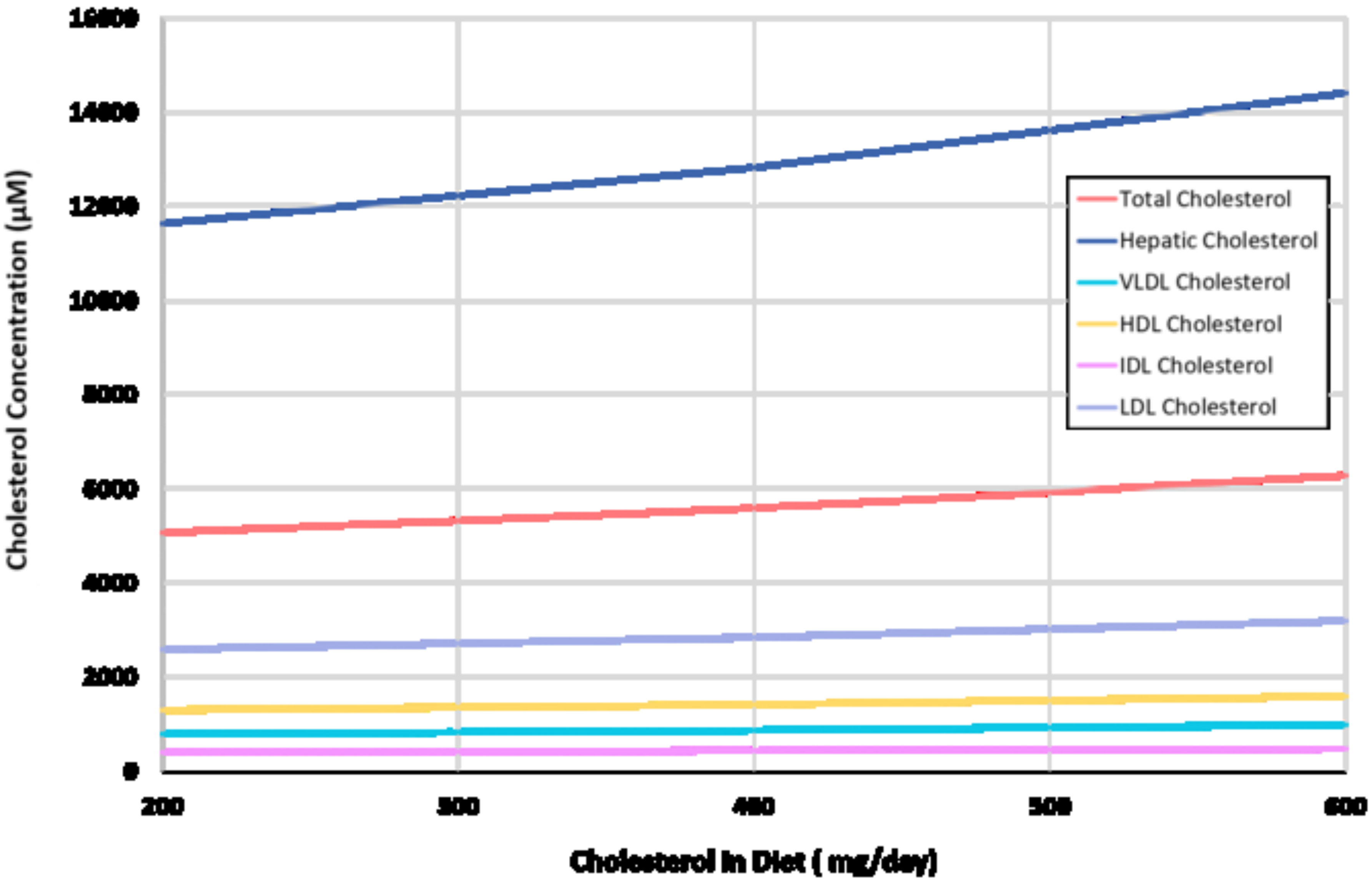

Figure 11

Click here to access/download;Figure;Figure 11.tif

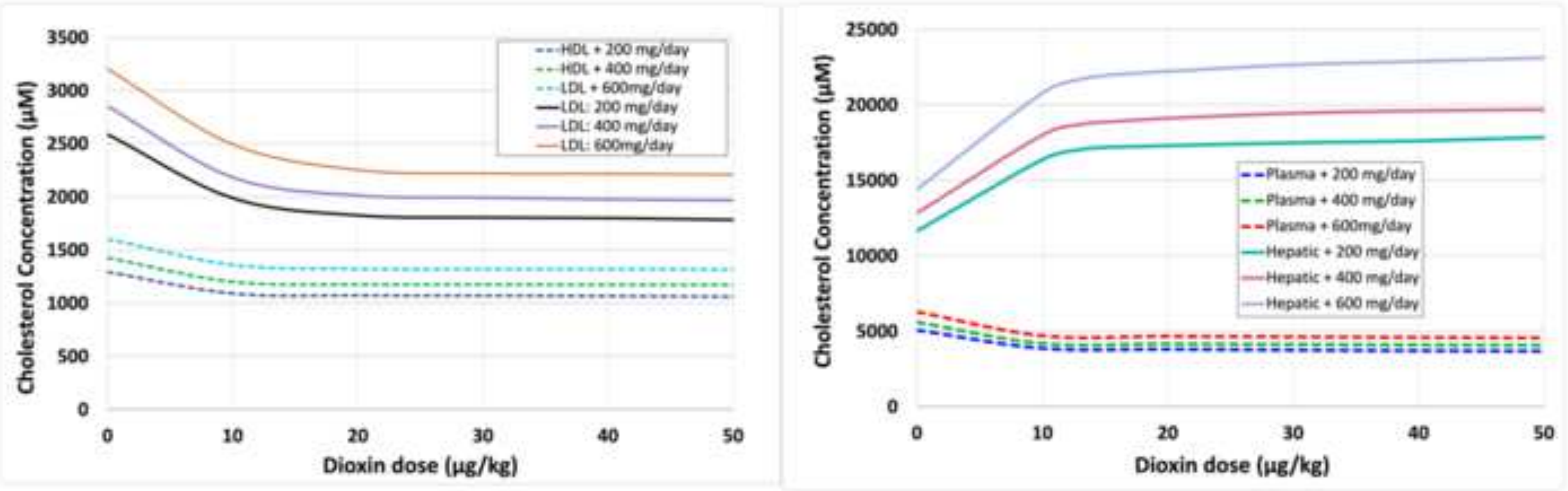

Figure 12

Click here to access/download;Figure;Figure 12.tif

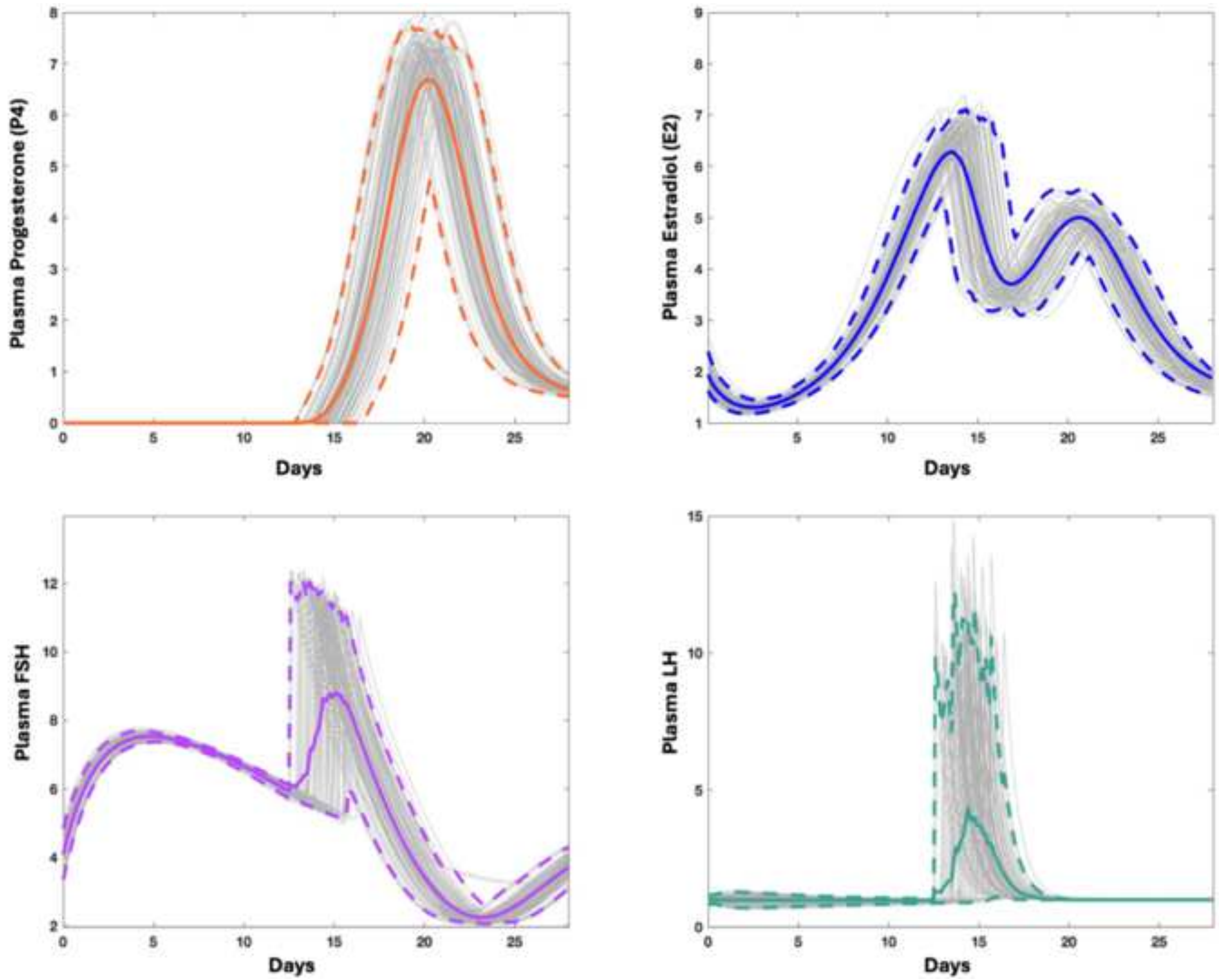

Figure 13

Click here to access/download;Figure;Figure 13.tif

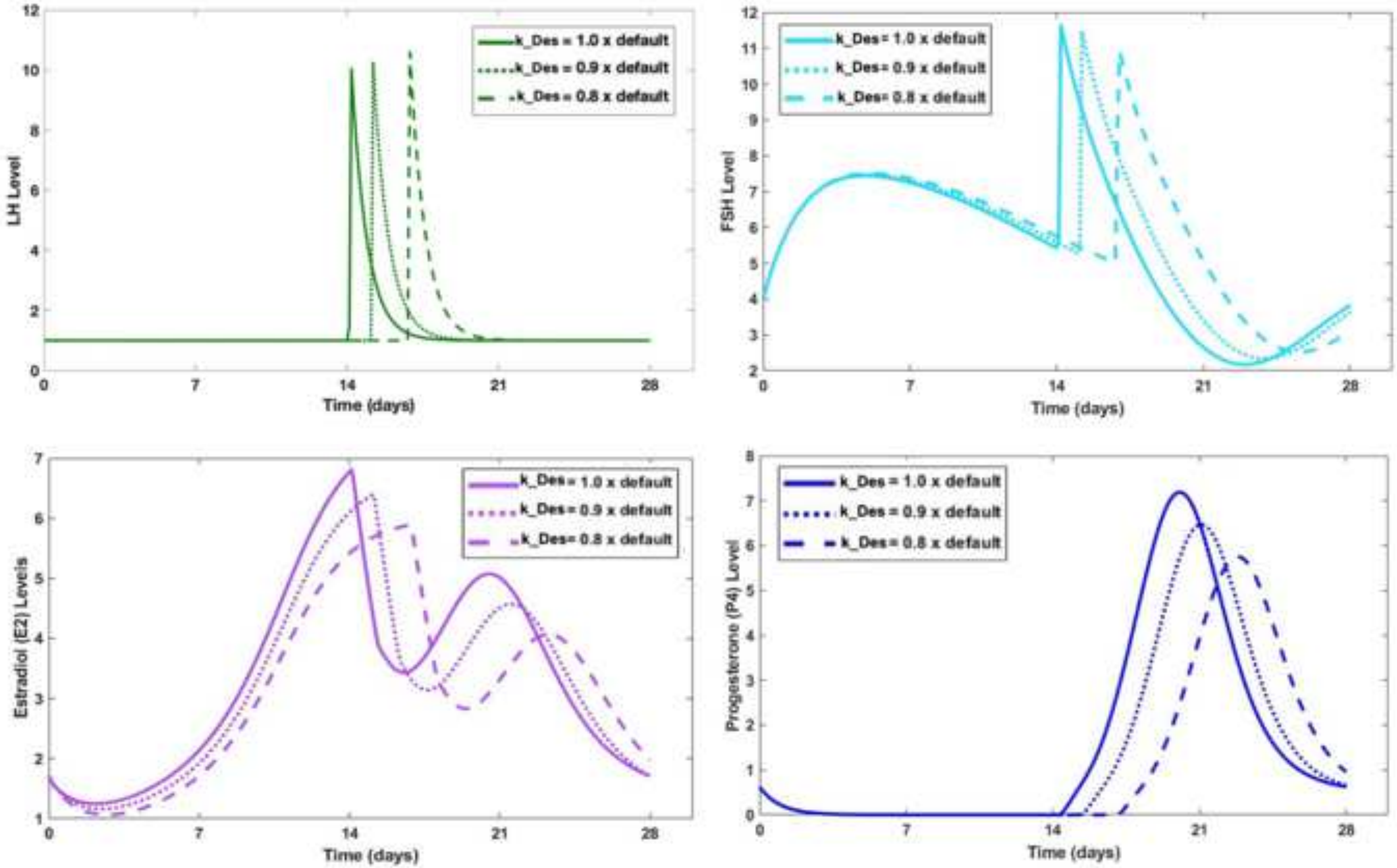

Figure 14

Click here to access/download;Figure;Figure 14.tif

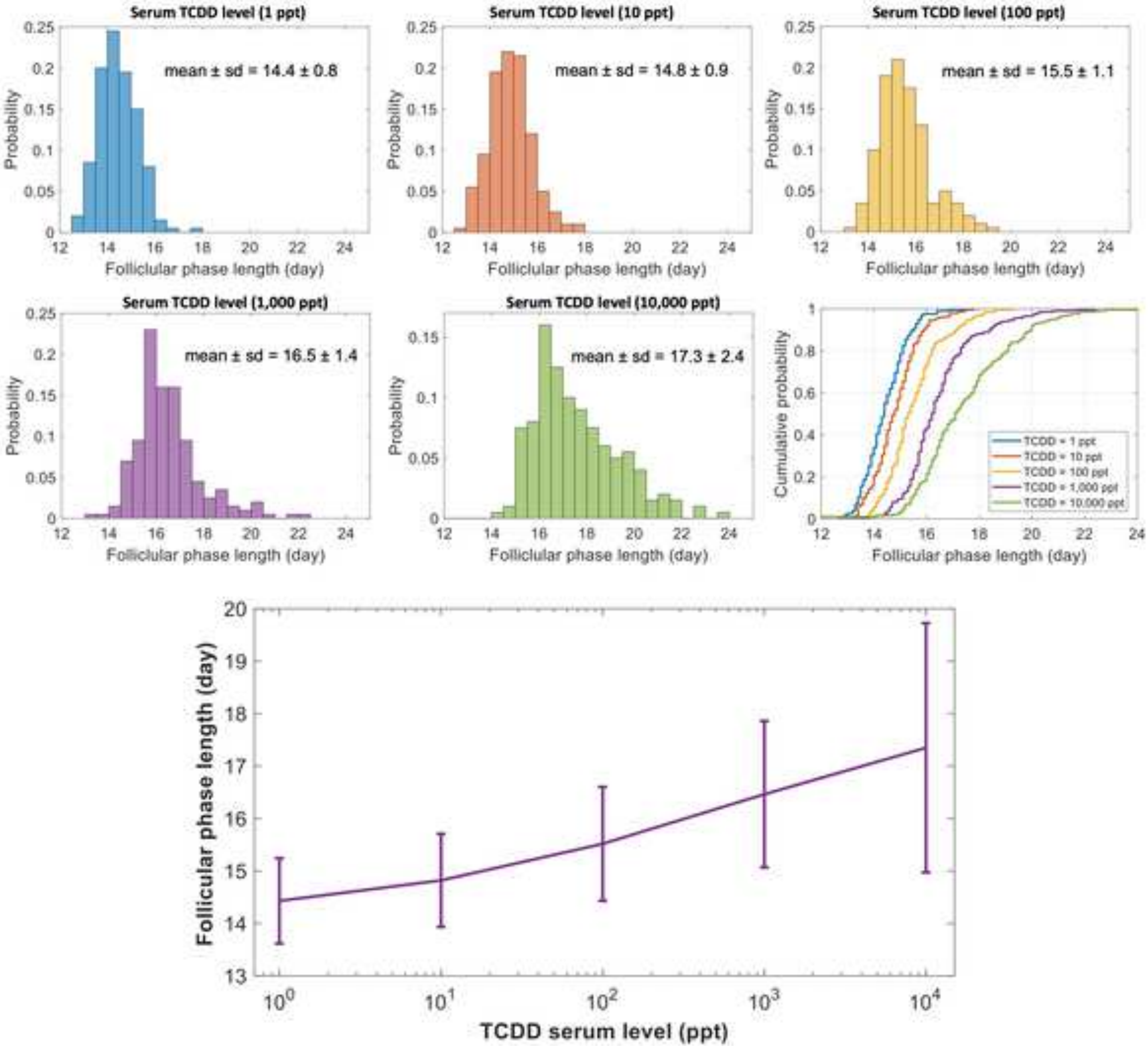

Figure 15

Click here to access/download;Figure;Figure 15.tif

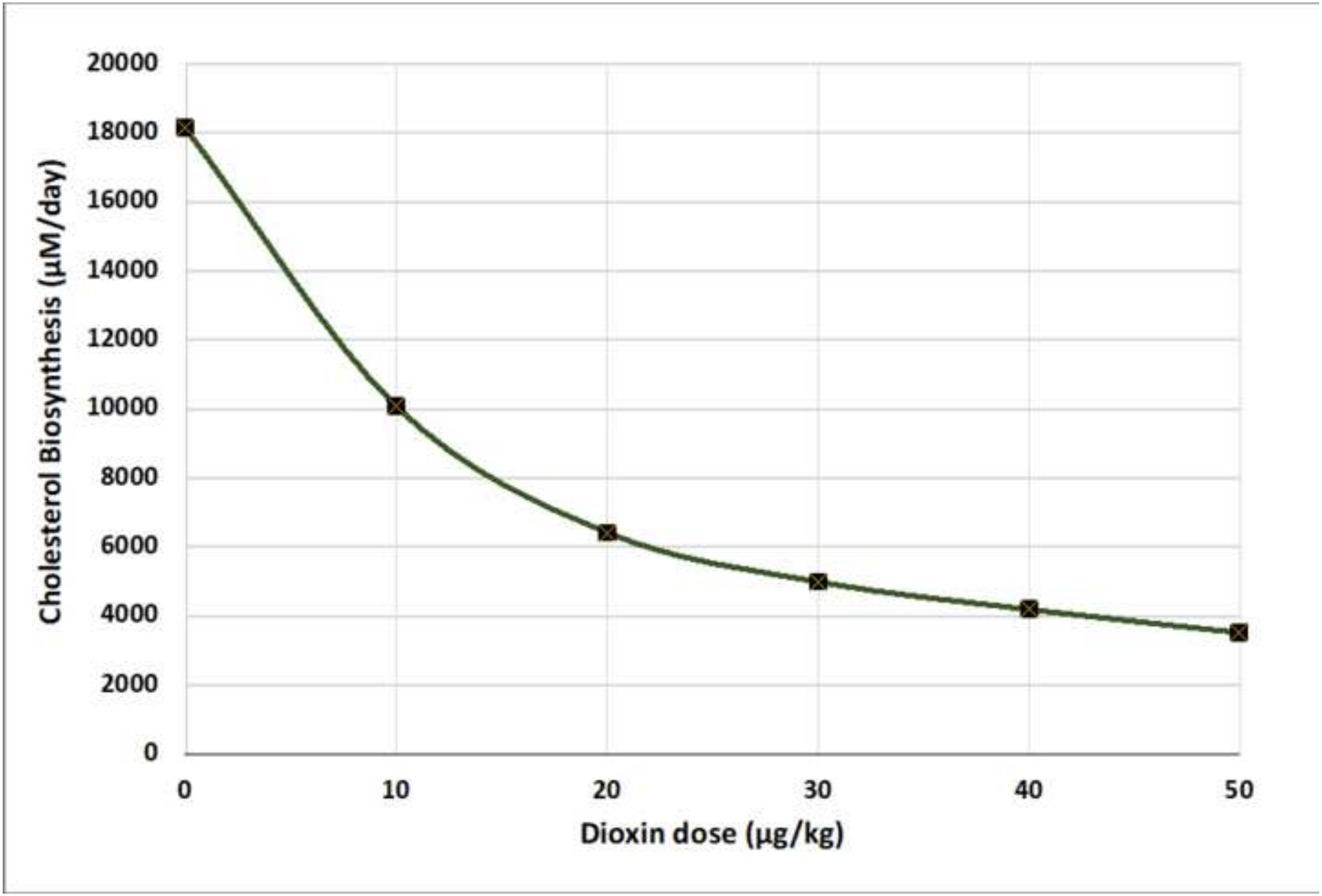

Figure 16 Click here to access/download;Figure;Figure 16.tif

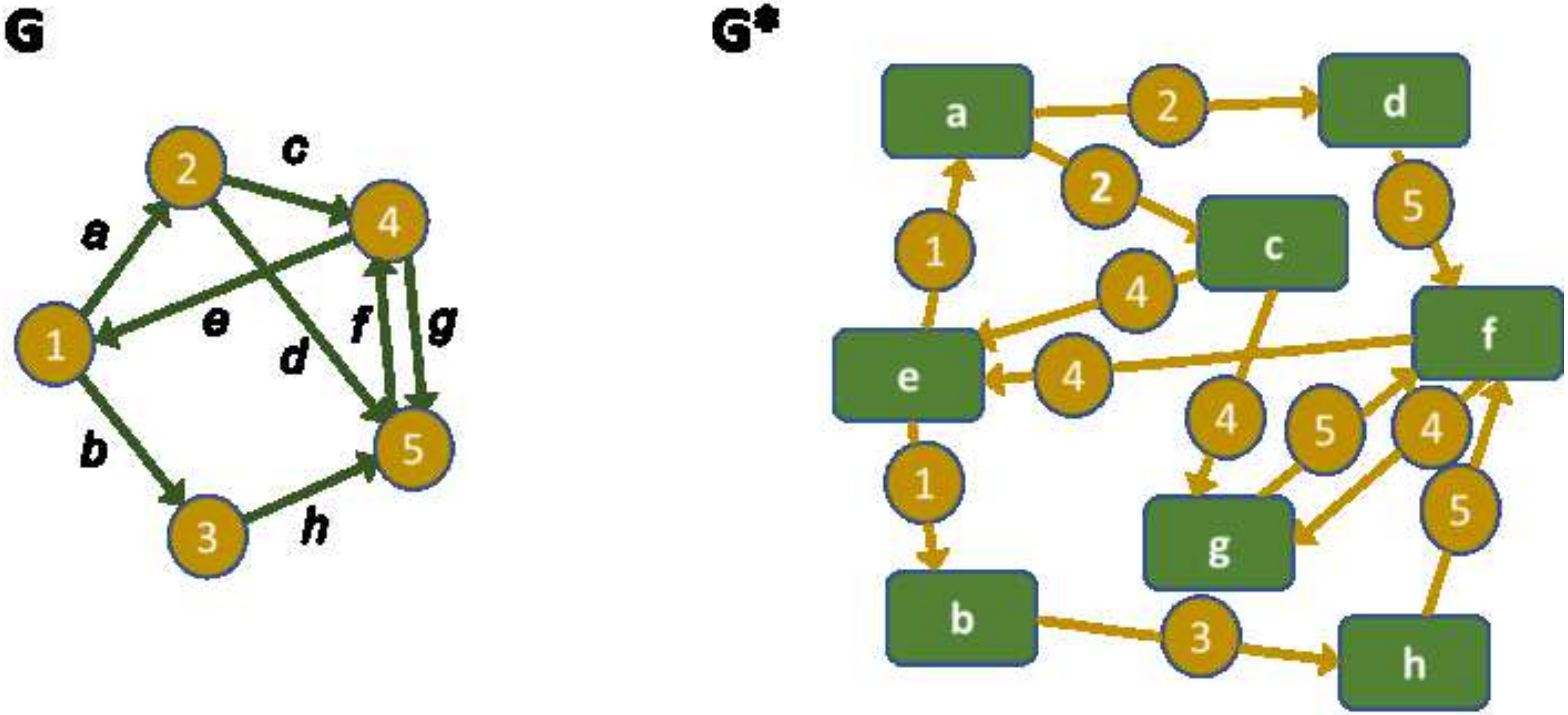

Click here to access/download;Figure;Figure 17.tif

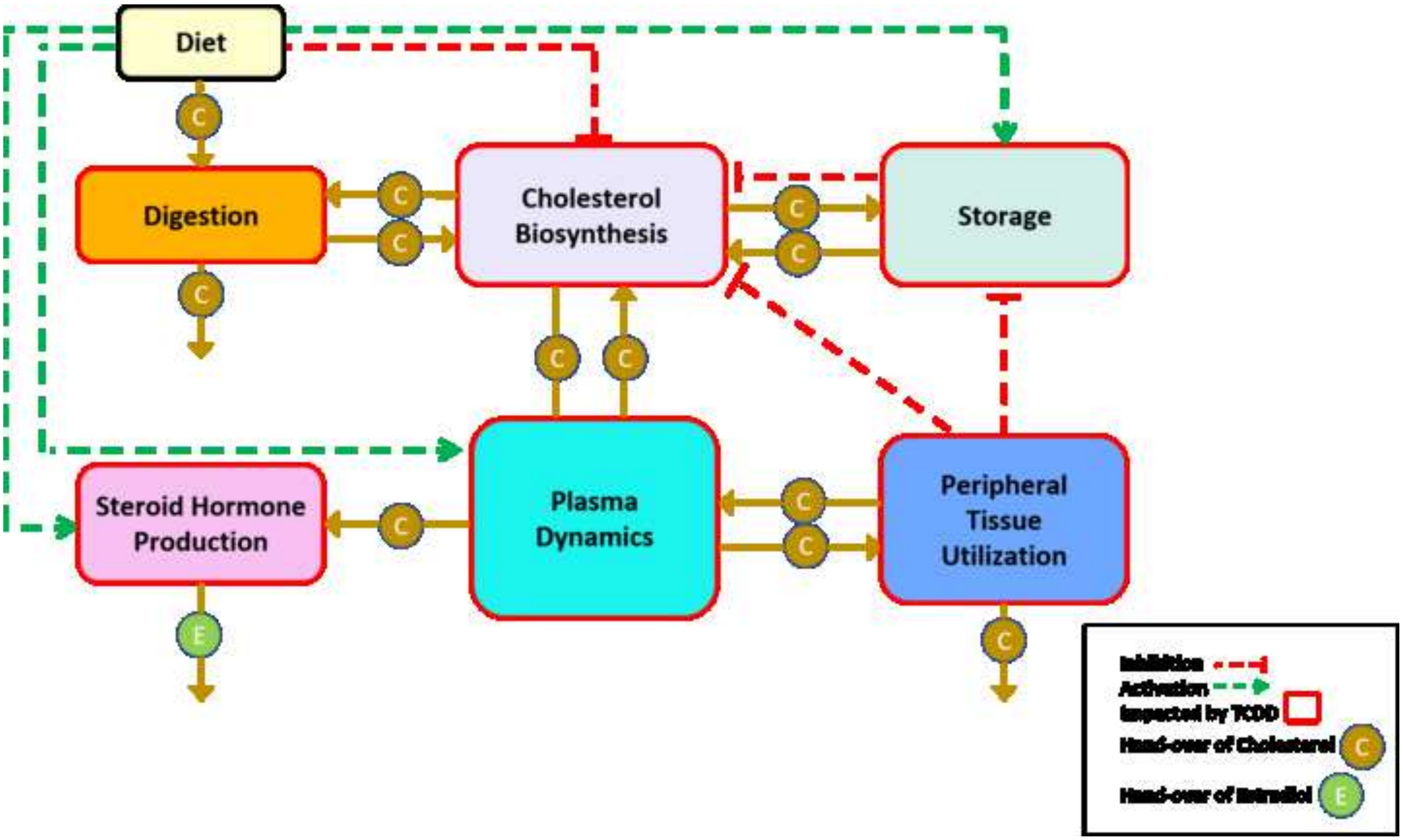

Figure 18

Click here to access/download;Figure;Figure 18.tif

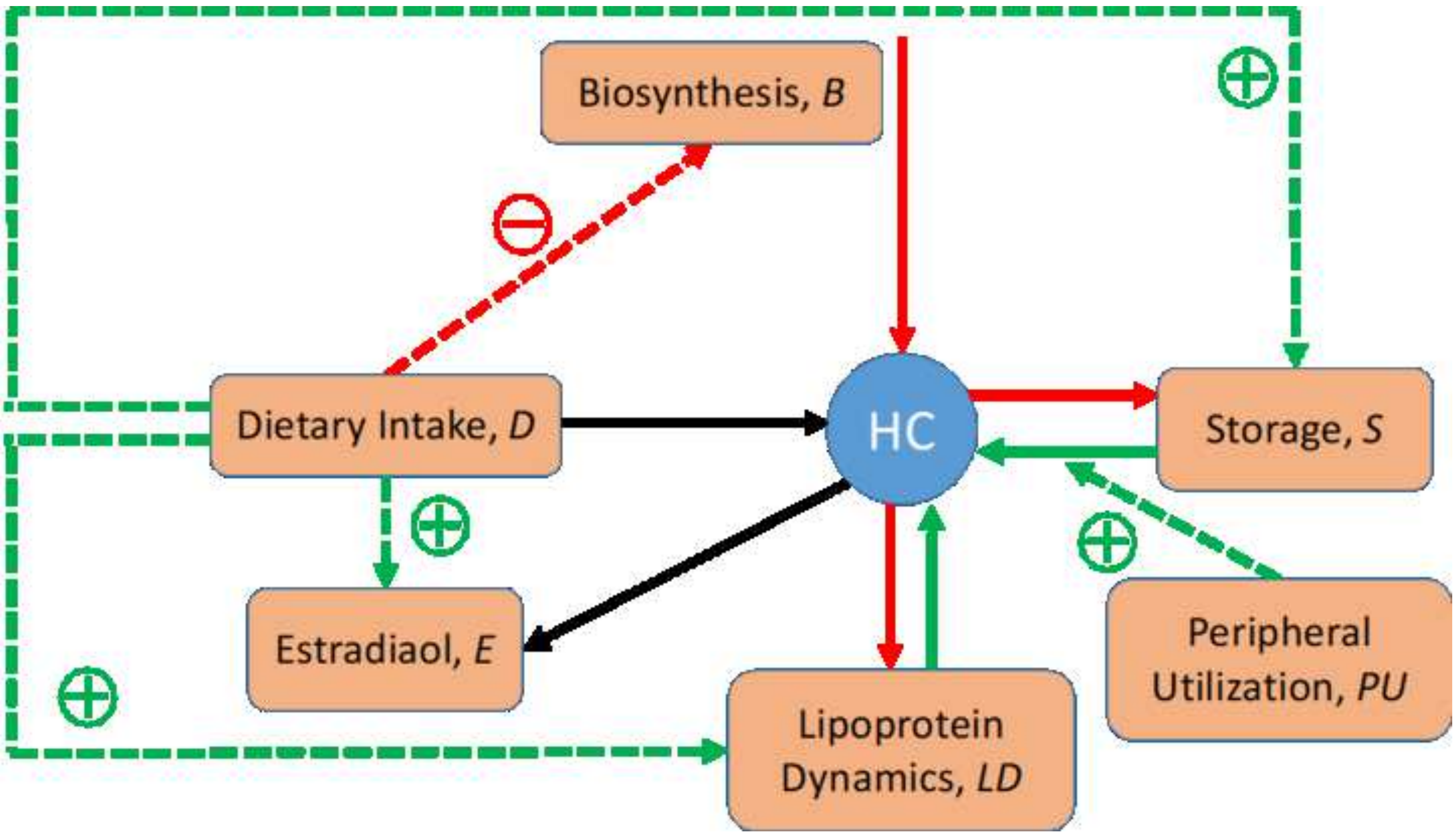

Click here to access/download
Supporting Information
New Paper Supplementary Material - submitted .docx

Multiscale Biomedical Systems Analysis through Template-and-Anchor Modeling

I. Conceptual and Theoretical Considerations

Carla M. Kumbale*, Qiang Zhang,*** , and Eberhard O. Voit*,***,******

* Department of Biomedical Engineering, Georgia Institute of Technology, Atlanta, GA 30332, USA.
** Gangarosa Department of Environmental Health, Rollins School of Public Health, Emory University, Atlanta, GA 30322, USA.
*** Co-corresponding author.
**** Department of Biological Sciences, University of Texas at Dallas, Richardson, TX 75080-3021, USA.

Email Addresses: Ckumbale3@gatech.edu; Qiang.zhang@emory.edu; Eberhard.voit@bme.gatech.edu

Abstract: We address the well-known challenge of biomedical multiscale analysis with a novel adaptation of the *Template-and-Anchor* (T&A) modeling paradigm. We consider a the template as a high-level, coarse-grained model that focuses exclusively on the main physiological components of a system and involves correspondingly few variables, processes, and parameters. Anchor models are modules of component sub-systems that provide more elaborate descriptions of specific biological details. This conceptual framework does not attempt to capture simultaneously all details within a single computable structure as many other multiscale models do. Instead, the often-overwhelming multilevel task is dissected into smaller, stand-alone models that are analyzed separately. Following the analysis of the lower-level anchors, results from this analysis are used to inform the template model. This new adaptation of the T&A approach offers substantial advantages without unduly losing resolution. First, the divide-and-conquer method enhances computational efficiency. Second, unlike other methods, each anchor is analyzed individually, producing a record of crucial input-output relationships that are ultimately used in the template model. Third, an anchor models can be interchanged replaced with alternate models without affecting the structure of the template or other anchors. Fourth, the T&A model provides flexible guidelines for its setup and for defining variables across scales.

TIn this article, we discuss concepts and theoretical aspects of th T&A approach and describe in broad strokes the setting up and analysis of the template and anchorT&A models. In the companion article [1], we illustrate the approach with a detailed investigation of he detailed methodology and the formalization of the T&A framework concepts are presented in our companion paper. Here, we demonstrate how the T&A framework facilitates a holistic evaluation of dioxin's impact on the liver, which can be translated into a comprehensive computational health risk assessment tool. Using this approach, we investigate the health implications of exposure to 2,3,7,8-tetrachlorodibenzo-p-dioxin (TCDD), a persistent organic pollutant which that can severely affect health, depending on the magnitude of exposure. We We first present a template capturingrepresenting the handling of cholesterol in the body and the overall effects of TCDD on the this dynamicsnetwoek of processes of cholesterol, a prominent target of TCDD. We then introduce several anchor models and demonstrate how information, in the form of dose-response curves, informs the template model.

We then briefly highlight one representative anchor model representing cholesterol handling by hepatocytes. The detailed methodology and the formalization of the T&A framework concepts are

Formatted: Font: 14 pt, Bold
Formatted: Normal
Formatted: Font:
Formatted: List Paragraph, Indent: Left: 0.75"
Formatted: Font color: Auto
Formatted: No underline, Font color: Auto
Formatted: No underline, Font color: Auto
Formatted: No underline, Font color: Auto
Formatted: No underline, Font color: Auto
Formatted: No underline, Font color: Auto
Formatted: No underline, Font color: Auto
Formatted: No underline, Font color: Auto
Formatted: Font color: Auto

~~presented in our companion paper. Here, we demonstrate how the T&A framework facilitates a holistic evaluation of dioxin's impact on the liver, which can be translated into a comprehensive computational health risk assessment tool.~~ ~~Overall, multiscale~~ Multiscale modeling, and ~~more~~ specifically T&A modeling, holds promise for a deeper understanding of complex systems and for advancing personalized medicine and health risk assessment, interspecies translation, and virtual clinical trials.

Keywords: Agent-Based Models; Dioxin; Dose-Response-Curve; Multiscale Modeling; Line Digraph; PBPK Model; Template-and-Anchor Model; Time Scale Separation

Background

Biological phenomena occur and interact across multiple levels of organization, from molecules and cells to tissues, organs, organisms, and ecosystems. Each level is intricately connected and may influence others, making the study of these multiscale systems complex and challenging. As a generic example, molecular interactions govern the behavior of proteins, nucleic acids, lipids, and other biological macromolecules. These molecular interactions manifest in cellular functions such as signal transduction, metabolic processes, and cell cycles ~~ligand binding and signal transduction~~. At the tissue level, cells interact through specific characteristics and play crucial roles in phenomena such as morphogenesis ~~hormonal signaling~~, which governs many aspects of anatomy and physiology. Thus, the collective behaviors of the various molecular processes within and among cells affect tissues, organs, organ systems and ultimately organisms that respond adequately to their environments.

Further complexity arises if one considers the interplay among different cell types, tissues, and organs, where a solid understanding of these relationships is key to deciphering biological processes such as digestion, circulation, and disease, to name just a few [2]. The intricacies of these processes are not only manifest in individual multi-cellular organisms, but quickly arise if one considers the interactions between organisms, populations, and entire ecosystems, wherein interactions among different environments and species introduce even greater complexity. These interactions in turn influence numerous phenomena, from population growth to the spread of infectious diseases and environmental changes.

The often-encountered multilevel complexity implies that traditional reductionist methods alone do not provide a comprehensive understanding of biomedical phenomena and mandates an integrated approach that takes into account interactions and dependencies across multiple scales [2-4]. For example, unraveling diseases such as cancer requires knowledge of how molecular-level interactions translate into higher-level effects within the body. Accordingly, cancer is often rightfully described as a systemic disease, due to the intricate interplay of factors at multiple levels that contribute to its onset and progression [5]. Namely, while cancer is known to be characterized by ill-regulated cell proliferation and the invasion into healthy tissues, its manifestation and behavior are influenced by a vast network of cellular and physiological interactions, such as immune responses. Furthermore, tumors are heterogeneous cellular assemblies, whose growth and metastatic potential depend upon dynamical interactions among the cancer cells themselves and their constantly changing microenvironment. Additional systemic factors, such as hormonal responses, have profound effects on the behavior of cancers [6, 7]. This complex web of interactions, which spans from the molecular to the organismal level, necessitates an integration of diverse processes on multiple levels for a better understanding and treatment of this disease [8].

Not surprisingly, substantial challenges exist for the design and development of computational models describing multiscale phenomena. The fundamental reason is the

Commented [KCM1]: Reviewer Comment: Create a set of sections or a detailed methodology (or a second accompanying manuscript) where the concepts are properly formalized.

Commented [KCM2R1]: Response: Thank you for your suggestion. We have structured a detailed methodology and formalized the concepts of the T&A framework, specifically modeling the TCDD-mediated effects on cholesterol dynamics and steroidogenesis, in a second accompanying manuscript.

Formatted: Font:

Formatted: Normal

Formatted: Indent: First line: 0"

Commented [ZQ3]: Ligand binding belongs to molecular interactions

vastly increased number of components at various scales, both temporally and spatially, and the need to define, quantify, and often adjust numerous parameters of different types that possibly interrelate with one another across scales. As a part of this challenge, defining the linkage between different scales is a substantial hurdle. Often, these links are bidirectional, implying that variables, parameters, and functions characterizing the biomedical system influence one another [8] [8]. The new added complexities associated with multiscale models confound the challenges already occurring when large numbers of variables, nonlinear interactions and emergent behaviors are at play on each level. These challenges, in turn, are manifest in cause increased computational demands, validation, and standardization. S, as simulating the dynamics across various scales can be computationally intensive, requiring specialized algorithms. A separate issue is the validation of these models, which may need unique criteria at each level, thereby potentially leading to compounded uncertainties. Finally, the lack of standardized methodologies across different domains complicates the analysis, comparison, and interpretation of multiscale systems [9].

Multiscale Modeling Approaches

In an overview on multiscale modeling in biology [2], the authors discussed the scientific progression and advances in scientific tools and technologies that have enableding the research community to explore complex biological systems that are inherently multi-scaled in nature. Future progress will require not only advancements of these methods, but also transdisciplinary collaboration among researchers in molecular and cellular biology, physiology, evolution, ecology, mathematics, bioinformatics [10] [Nersessian, 20222 #268][ ] and what is now called data science [11][10]. [ ]. This collaboration is needed not just to obtain, organize, and analyze the massive volumes of data generated through experimentation, but for constructing, improving, and standardizing comprehensive models that can elucidate the functioning of life across different temporal and spatial scales. With this mandate for collaboration comes the need for improved transdisciplinary communication (Chapter 19 of [12]).

Numerous structured and *ad hoc* approaches to multiscale modeling have been proposed to capture the intricate interactions and phenomena occurring across different scales of biological systems. Two very different yet representative examples are physiologically based pharmacokinetic (PBPK) models and agent-based models (ABM).

Physiologically based pharmacokinetic (PBPK) models simulate the absorption, distribution, metabolism, and excretion of drugs or chemicals toxicants within living organisms (**Figure 1**). These models are structured to represent in a somewhat simplistic manner the actual physiological architecture of the organism and take the format of a hybrid between a compartment model at the organismal level and a set of kinetic models within the compartments [13, 14]. What renders PBPK models inherently multiscale is their ability to bridge different levels of biological organization. At the molecular level, they address interactions like drug lipid partitioning into lipid, as it occurs in fatty tissue, while they account at the cellular level for processes like drug uptake and metabolism, for instance, in the liver. Moving up the scale, they define compartments for specific organs or tissues, each with its distinct drug-related parameters. Collectively, the integrative modeling of these different components enables a holistic view of how a substance behaves across the entire organism, from initial drug administration to eventual elimination, thus offering a comprehensive perspective that spans multiple biological scales and—on the practical side—offers guidance regarding drug doses and administration regimens.

Formatted: Indent: First line: 0"

Commented [VEO4]: Baumer, B.S., D.T. Kaplan, and N.J. Horton, *Modern Data Science with R (2nd Ed.)*. 2021: CRC Press.

Formatted: Highlight

Formatted: Not Highlight

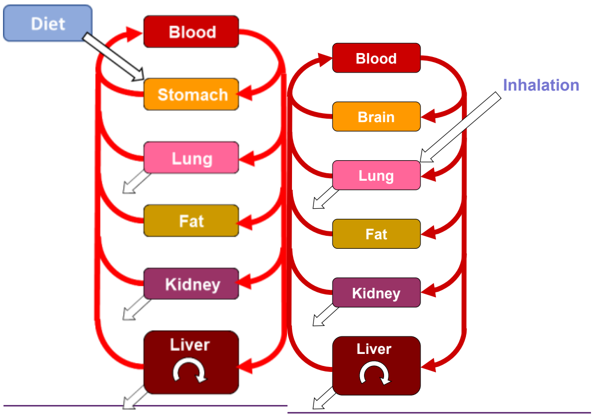

Figure 1. Generic PBPK model as a multiscale structure. The primary scale of granularity reflects organs and tissues, but the drug concentrations in these organs are subject to molecular and cellular events, such as sequestration and degradation in the liver. In this illustration, the drug or toxicant enters the body per inhalation.

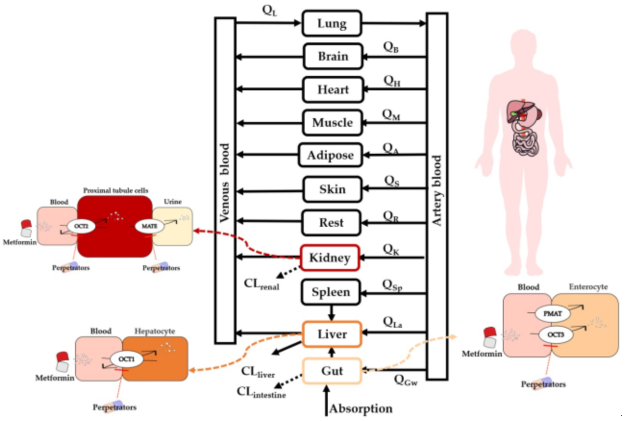

The second example is an agent-based model (ABM), which is a generic stochastic approach that simulates the actions and interactions of autonomous agents, which are individual entities with distinct attributes and behaviors [13, 15]. The agents typically move on a grid and can represent a myriad of entities, ranging from molecules and cells in biological systems to individuals in social scenarios. Governed by predefined rules, ABM agents can adapt, learn, and evolve based on their experiences and interactions within their environment. This modeling approach is inherently multiscale because it can span a wide range from the micro-level where, for instance, agents might represent cells or individuals, to the macro-level, where tissue function or societal dynamics emerge. The temporal aspect of ABMs can capture both immediate agent decisions and long-term system evolutions. Further, ABMs can involve spatial differences and capture behaviors that arise from numerous individuals dynamically interacting according to preset collective properties [16, 17]. More generally, multiscale modeling often adopts a “hybrid” approach in

Formatted: Centered

which the actions of migrating agents may be governed by systems of differential equations, which are updated at every discrete step of the ABM [18-21]. Several other types of hybrid models have been discussed in the literature [22-24]. Due to their intuitive nature and flexibility, ABMs are increasingly used as teaching tools in biology classes [25].

Technical Challenges of Multiscale Modeling

The organization of biological systems as distinct but connected layers poses one of the grand challenges for biomathematical modeling, because processes occurring at the various layers often have different time scales and almost always focus on different types of variables. The higher layers usually correspond to a "big picture" of physiological events, whereas the lower levels account for increasing granularity and detail. When investigating a system at a high level, it is often infeasible to carry along all details from lower levels, partly for technical reasons, but also because they would overwhelm insights at the higher level due to their sheer numbers and the fact that they typically run on much faster time scales. For instance, the growth of an embryo from a fertilized oocyte throughout the phases of blastula, gastrula and further development may span several weeks, during which uncounted metabolic and cellular processes occur at a much more rapid pace.

Formatted: Indent: First line: 0"

A partial solution that was proposed a long time ago is time-scale separation, which yields a reduction in model size (*e.g.*, [26]). As an explanation for the validity of this reduction, it has been argued ~~(*e.g.*, [26, 27])~~ that the processes at lower levels are so fast that they are essentially always in a quasi-steady state, which permits their conversion from differential equations to explicit algebraic equations that may be entered as constraints in a much smaller set of governing differential equations (*e.g.*, [27, 28]). As an example, the clouding of the human lens is a very slow, life-long process that is caused by clearly identifiable biochemical reactions that occur on an ongoing basis at the sub-second scale. In this case, the biochemical aspects may be analyzed separately from the slow clouding process during aging [29]. While separation of time scales is certainly an option for processes with time constants at different orders of magnitude, such a separation is not feasible if the layers of biological organization are not all that distant. For instance, the dynamics of calcium exchanges in the heart is fast in comparison to macroscopic heart physiology, but it is close to impossible to separate the two [30]. In another example, ~~discussed later in detail,~~ the direct effects of ~~dioxin~~ exposure to an environmental toxicant on human health are often manifest at the transcriptional and metabolic levels, which have similar time scales, although their consequences may, over the span of years, lead to ~~liver~~ a spectrum of disease~~, reproductive dysfunction, and possibly cancers~~.

Template and Anchor (T&A) Modeling~~:~~

To address the various challenges inherent in multiscale modeling, we propose a modeling paradigm suggested over two decades ago, but seldom actually implemented, namely Template-and-Anchor (T&A) modeling [31]. A template is a high-level model that focuses exclusively on the main physiological components of a system and involves correspondingly few variables, processes, and parameters. It provides a coarse-grained representation of the system under investigation. Anchor models provide more elaborate views of the specific biological details characterizing the mechanisms that govern the system and are represented in the template model as variables.

Formatted: Indent: First line: 0"

The ~~original~~ authors and proponents of the original T&A ~~modeling~~concept, Full and Koditschek [31], considered this approach most useful not for multiscale modeling *per se*, but for the embedding of a generic design principle ~~into~~ within a space of alternate implementations. Expressed differently, they considered the template model as a general design principle and the anchor models as differently structured implementations of this common design. Their key example assessed the locomotion of terrestrial animals, which the authors demonstrated ~~to have~~ numerous, quite different manifestations (which they called "degrees of freedom"), such as the number and structure of legs. While distinctly different, all implementations had to comply with a common ~~det~~ set of biomechanical

Commented [QZ5]: What is det?

design features. Kvalheim and Revzen [32] formally investigated this aspect of the T&A approach from a standpoint of control theory. They embedded all anchors in a high-dimensional space such that their intersection consisted of the lower-dimensional template, which was thereby seen as the common design principle that all implementations had to satisfy. As the authors expressed it, "the template is not only a description of a typical subset of poses ((of a galloping horse)), but also a low-dimensional dynamical model that captures features of the aggregate behavior of the body."

Our focus here is not on an embedding of different implementations of a design feature but on a method for praegmatically tamingreducing complexity, which is accomplished by dissecting a complex modeling task into several smaller, better manageable tasks. The first of these is the formulation of a template model as a representation of the entire problem space of interest. It reflects processes at some fixed level of biological organization. As is typical in dynamic modeling, this template containsis composed of variables that in-teract dynamically and are formulated as explicit functions with appropriate parameters. The model may be assessed with the usual steady-state analyses (e.g., such as the characterizingation of fixed points and their stability and sensitivities), as well as with computational simulations of representative trajectories. The second task is the formulation of anchor models, which correspond to the variables of the template and capture in detail underlying processes in detail.

Formatted ...

As an example, suppose the template represents a typical PBPK model (Figure 1), where the components are specific organs or tissues. Each such component ultimately becomes an anchor. One of these will describe how a substancedrug enters the liver from the bloodstream, is partially degraded by enzymes, partially stored, and partially released into bile or again into the bloodstream. Suppose now that some perturbation, such as exposure to some toxicant, alters the enzymatic activities within the liver. This perturbation is analyzed at the molecular level with the anchor model, resulting in an altered distribution between degradation, storage, and release. The overall change in output, that is, release of the drug into the bloodstream, caused by the toxicant, is then used to inform the template PBPK model under exposure.

Formatted ...

WeAs is typical for about of any modeling effort, consider the first step of the model design and analysis is of any modeling effort to be the creation of a box-and-arrow diagram [13]m [Voit book], and T&A models are no exception. Consequentyllly, the T&A diagrams resembleve typicalAt first glance, the diagrams utilized in Template and Anchor (T&A) models may look somewhat similar to block diagrams that are common utilized in control theory to model engineered dynamical systems. However, theThese block diagrams in control theory schematically depicts how an input signal to a system undergoes various transformations through blocks of transfer functions, including the potential forward flow of a signal or feedback signals. These transfer functions are nothing other than typically modeled in the signal domain as the Laplace Transform transforms of the mathematical functions that transmuteits an input signal to an output signal in S the signal domain. The Due to the assumed linearity of the engineered system, the transformations of an input signal to an output in a block diagrams are relatively simple and may include feedback signals or the as a forward flow of a signalinformation or signals with perhaps certain feedback taken into account. Such signals could also apply to biomedical systems, but these are almost always nonlinear, thereby excluding Laplace transforms from the repertoire of applicable methods. In particular, bothOn the other hand, the interaction of various biological systems are far more complex and even mutually influence each other. Thus, metabolic pathways are more complicated than the linear and forward flow of a signal undergoing relatively simple mathematical transformation in manmade control systems. Lastly, the template and anchor models capture the dynamic behaviour of processes that mutually interacting processes in biological systems in complicated ways.

Formatted ...

Of importance in our context is thatfor the T&A approach, the variables of the template model, when zoomed in, are fully functional, in themselves more finely grained dynamic models, which serve as anchor models. These anchor models provide a much more

Formatted ...

detailed view than typical a block diagrams, and a visualization would require something like a nested block diagram, with nonlinear influences flowing up and down the hierarchy of the diagram.

As a consequence of the T&A organization, tof specific subsystems or processes that are simplified and summarized within the template model. Intriguingly, within an anchor model, another set of anchor models can exist, transforming the original anchor model into a new template model for this new set of anchor models. The anchor models are can be analyzed separately across applicable scenarios, yielding results of various types. Many results of an anchor analysis, such as the concentrations of intermediates in a pathway, are important for the anchor itself, but not outside the anchor. By contrast, a few and the main outcomekey results of each anchor analysis are pertinent for the overall dynamics of the system. Theyse key results is become a functional or tabulated record of all pertinent input-output relationships. These relationships and arethat is subsequently used to connect toinform other anchors and , to calibrate the –template model (see companion article [1]).

In the end, the template model can be simulated without simultaneously simulating the anchor models while retaining and utilizing pertinent results from all anchors. This strategy of "logisticorganizational level separation" is different from typical multiscale models, which usually that attempt to represent all information at once and carry it forward in some fashion. By separating the levels, the T&A approach simplifies the overall modeling process, focusing on high-level processes while receivtaining detailed insightsinformation from more granular analyses. This separation not only aids in managing complexity but also enhances computational efficiency and model scalability.

Example

It is beneficial for further discussions to introduce the main example of this our analysis, the effect of TCDD (2,3,7,8-tetrachlorodibenzo-p-dioxin) on human health and, especially, on the dynamics handling of cholesterol and the sequelae of exposure (see below and [1] for details); for linguistic variationsimplicity, we use the terms *TCDD* and *dioxin* interchangeably.

TCDD Dioxin is known to affect the expression of several genes associated with cholesterol biosynthesis in a process mediated by the xenobiotic aryl hydrocarbon receptor AhR [33, 34]. The study of dioxin is important due to widespread processes generating dioxins as a by-products, such as municipal waste incineration and wildfires, which have resulted in about every person worldwide to be exposed to dioxins to some degree [35]. The problem is confounded by dioxin's long biological half-life, which causes it to remain and gradually accumulate in human tissues for several decades after exposure [36].

Several options are feasible to structure this problem space as a T&A system. A typicalThe template we chose for our illustration, specifically focusing focuses on the cholesterol handling by various organ systems in the human body and on alterations in these processes caused by dioxin, is shown in (**Figure 2**;). The boxes are represent the dependent variables of the template model, while the arrows indicate material fluxes among them. Ultimately, each box is will be modeled separately as an anchor model. A different template within the same general problem space could highlight the role of dioxin in carcinogenesis.

Figure 2 displays the components of the chosen template, and the text below provides a cursory overview of the problem space. The Specific technMethodological details regarding the desidesign and analysis of template and anchor and template models associated with this problem spacetopic at hand can be foundare provided in the companion paper [1]r [ ].

Formatted ...
Formatted ...
Formatted ...
Formatted ...
Formatted ...
Formatted ...
Formatted ...
Formatted ...
Formatted ...
Formatted ...
Formatted ...
Formatted ...
Formatted ...
Formatted ...
Formatted ...
Formatted ...
Formatted ...
Formatted ...
Formatted ...
Formatted ...
Formatted ...
Formatted ...
Formatted ...
Formatted ...
Formatted ...
Formatted ...
Formatted ...
Formatted ...
Formatted ...
Formatted ...
Formatted ...
Formatted ...
Formatted ...
Formatted ...
Formatted ...
Formatted ...
Formatted ...
Formatted ...
Formatted ...
Formatted ...
Formatted ...
Formatted ...
Formatted ...

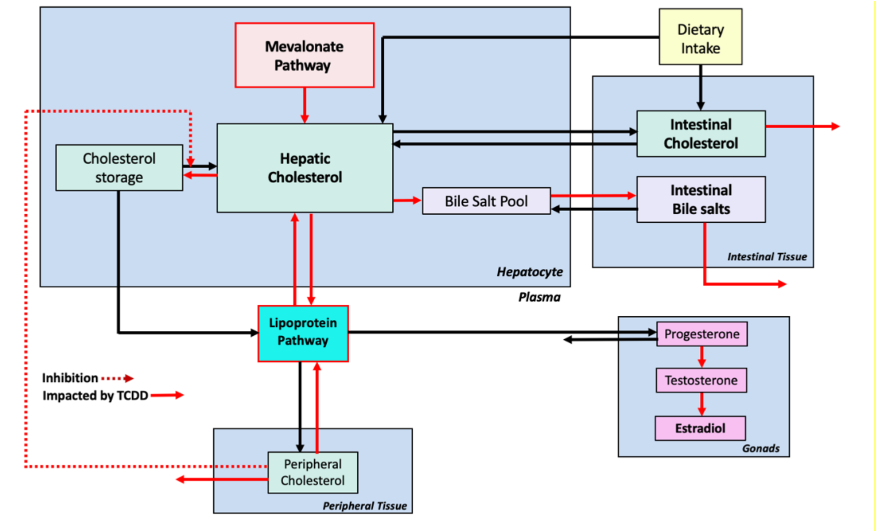

Figure 2. Template model of ~~TCDD~~ dioxin exposure affecting organism-wide cholesterol ~~dynamics~~ handling at a systemic level. ~~The main focus here is on the mevalonate pathway, which synthesizes cholesterol *de novo*.~~ Processes affected by ~~TCDD~~ dioxin exposure are indicated by red arrows. Adapted from [37].

As an illustration, we consider one anchor model, namely the mevalonate pathway in the liver, which is the main source of endogenous cholesterol. It is depicted in **Figure 3.** This anchor is particularly important because several steps within the mevalonate pathway are affected by dioxin exposure. Biochemical details of this anchor model were discussed in [37]~~[]~~; other anchors are discussed in the companion article [1]~~[]~~.

Formatted: Not Highlight

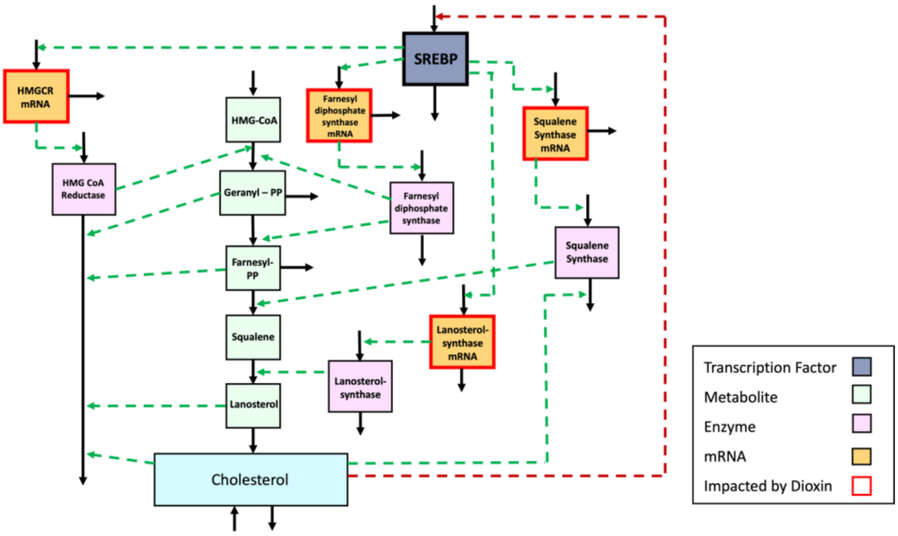

Figure 3. Hepatic cholesterol biosynthesis serves as a representative anchor model. The various components of the biosynthetic mevalonate pathway system are color-coded by type, as indicated. Metabolic reactions, transport steps, and the production of mRNAs and proteins are shown as solid lines, while regulatory signals, representing feedback inhibition (red) and modulation of enzyme activity [38], are given as dotted lines; mRNAs whose transcription is affected by dioxin, are indicated by boxes outlined in red. The key transcription factor SREBP binds to sterol regulatory elements (SREs) in the promoter regions of its target genes. It controls the activity of mevalonate pathway via these target genes, among which together with HMG CoA (3-hydroxy-3-methyl-glutaryl-coenzyme A) reductase, which is considered the rate-limiting step of the pathway, even though control of the pathway is distributed. Adapted from [37].

This anchor model contains as variables the metabolites of the pathway, as well as pertinent mRNAs, enzymes, and the important transcription factor SREBP, which is controlled by the amount of hepatic cholesterol through feedback inhibition. It is straightforward to set up a metabolic model for this system (see, *e.g.*, [39-41]). Literature searches directly or indirectly yielded parameter values, as well as information quantifying the effect of TCDDdioxin. The dDetails of this model design and calibration methods were are presented elsewhere in detail [1, 37] [39, 40]. Code for the models is available in the Supplements of the companion article and on GitHub. We will also submit representative models to Biomodels.

Various simulation scenarios are possible with T&Athis anchor models. For instance, one we can may simulate different magnitudes of dioxinTCDD exposure, analyze the effects of varying dietary cholesterol intake on the system, and test how these variations, combined with dioxinTCDD exposure, impact hepatic and serum cholesterol levels [37]. As an illustration, we present below one model result of the an analysis of different a series of dioxinTCDD concentrations on the steady-state concentration of hepatic cholesterol. Interpolating these results yieldsin the format of a dose-response relationship between dioxinTCDD and the anchor's main output variable, hepatic cholesterol (**Figure 4**).

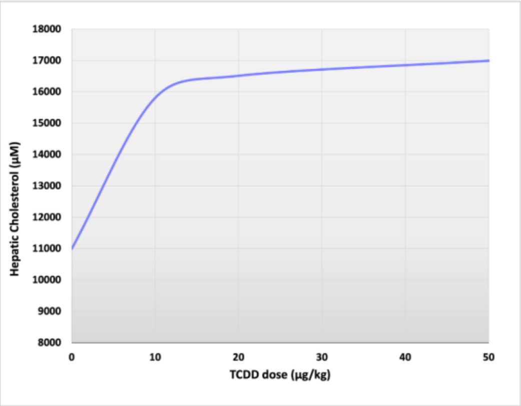

Figure 4. Simulation results with the model are formulated as a dose-response relationship characterizing the effect of dioxin (TCDD) on the steady-state amount of hepatic cholesterol produced by the mevalonate pathway. Adapted from [37].

The amount of cholesterol produced under continuous exposure to any relevant dioxin TCDD dose of interest can be extracted from this dose-response relationship and will eventually be handed off to other variables of the template model. Specifically, the dose-response relationship will be used as a dioxinTCDD-dependent modulator of the healthy

Formatted: Font color: Auto

Formatted: No underline, Font color: Auto

Formatted: No underline, Font color: Auto, Not Highlight

Formatted: No underline, Font color: Auto

Formatted: No underline, Font color: Auto, Not Highlight

Formatted: No underline, Font color: Auto

Commented [QZ6]: The citation should be our mevalonate pathway modeling paper, right?

Formatted: No underline, Font color: Auto, Not Highlight

Formatted: No underline, Font color: Auto, Not Highlight

Formatted: No underline, Font color: Auto

Formatted: No underline, Font color: Auto

Formatted: Font color: Auto

Formatted: Not Highlight

Formatted: Font color: Auto

baseline flux (TCDD = 0) between the mevalonate entering the pathway and hepatic cholesterol level (see Figure 2).

~~In the future, o~~Other anchor models ~~presented in~~ within the template ~~model will be developed~~are presented in the companion article [1]~~[,]~~; they include detailed models of lipoprotein dynamics and~~such as a~~ the reproductive endocrine ~~model~~system. ~~Substituting all variables of the template model with output information from these anchor models will ultimately allow us to assess the global effects of dioxin exposure on the human body, which has seldom been done before. Given adequate input data from individuals, the T&A approach will permit a personalized human health risk assessment.~~ These anchor models can be used to generate numerous results. ~~Of interest~~Important for the given task of assessing the effects of dioxin, only some key results are retained and used for calibration of the template model. Namely, we ~~will~~ record how dioxin affects the output of these anchors, which enter other anchors as inputs. Specifically, we record dose-response relationships between dioxin exposure levels and output, such as the ~~amount~~~~rate~~amount of cholesterol generated by the mevaolante pathway.

Collecting all these relationships, and using them as modulators of variables in the template model, ~~Substituting all variables of the template model with output information from these anchor models~~ will ultimately allow us to assess the ~~global~~systemic effects of dioxin exposure on the human body~~, which has seldom been done before~~. Given ~~adequate~~ input data from specific individuals that are of sufficient information content and quality, the T&A approach will permit a personalized human health risk assessment; methods for this type of personalization have been presented elsewhere [42-44]~~[Voit and Brigham; Davis et al.; Kumbale and Voit]~~.

Advantages of T&A Modeling

If sufficient data are available, the T&A approach offers substantial advantages without losing much resolution. First, the divide-and-conquer approach greatly increases computational efficiency, as the anchors are analyzed with methods devised for their specific levels; in other words, problems of data heterogeneity are minimized. Second, since anchor models can be designed and analyzed in isolation, the T&A structure can be developed gradually by involving output information from increasingly more—or more sophisticated—~~a~~ anchors, while ~~ill~~less well-defined components of the template remain as simple variables. Thus, the approach offers flexible guidance for setting up the model and for the definition of variables and processes at the different stages of the modeling process. Third, in contrast to other multiscale approaches, anchors are not repeatedly analyzed together with other anchors and the template. Instead, each anchor is analyzed separately for all applicable scenarios and the main output consists of a functional or tabular record of all pertinent input-output relationships, which are later used to inform other anchors and to calibrate the template model. Fourth, the anchor models can be "hot-swapped" in a sense that they may be replaced, if necessary, with more detailed models, while other anchors and the template remain intact. We indicate this in our companion paper by developing a detailed ovarian steroidogenesis~~estradiol synthesis~~ model, which we then simplified for the template analysis [1].

We have shown elsewhere [42, 43] how population-based models may be personalized, and the T&A approach could be personalized in a conceptually straightforward manner (even though substantial information would be needed for such an implementation). At any rate, the structure of the T&A approach is no hindrance toward personalization. As an indication in this direction, we previously studied interactions between dioxin exposure and dietary cholesterol uptake [37]~~[]~~, where available individual exposure and dietary information can be applied to the model.

Theoretical Considerations Regarding the Structure of T&A Models

Formatted: Not Highlight
Formatted: Not Highlight
Formatted: Not Highlight
Formatted: Highlight
Formatted: Not Highlight
Formatted: Indent: First line: 0"
Formatted: Not Highlight
Formatted: Indent: Left: 0", First line: 0"

We have been using T&A models in a ~~straightforward~~n intuitive manner, ~~according to the intuitive~~adopting the ~~-~~notion of a template model as a high-level, coarse-grained umbrella structure and the anchors as models elucidating parts of the template in greater detail. At first glance, the mathematical structures of template and anchor models seem to be simple variations of the same theme. Closer inspection, however, reveals an interesting combination of differences and correspondences between template and anchor models in general. This combination is most easily seen for a dynamic system operating at a steady state. At this prominent state, the structures of anchor and ~~the two types of~~template models ~~have the structures of~~conceptually resemble ~~a "regular directed~~dual graphs" ~~(anchor model) and an edge-to-vertex dual graph (template model), respectively [44]~~ (*e.g.*, [45] ~~[85]~~). The topic of dual graphs has been discussed in the graph theor~~yetical~~ literature for a long time, using different terms, primarily "line graphs," but also "edge graphs" and "edge-to-vertex duals," among others. Line graphs were arguably first mentioned by Whitney in 1931/1932 [46]~~[ ]~~. A good review is the 1972 book by Harary and, in particular, the chapter on line graphs [47]~~ within this book~~.

In the language of graph theory, the line graph G* of a graph G is constructed by exchanging edges and vertices. In our case, the model structures at steady state are of course directed graphs ("digraphs"), and the analogy in this situation is therefore called a "line digraph" [47, 48]~~[Harary and Norman (1960) and Hemminger and Beineke (1978)]~~. ~~In the language of graph theory, the dual graph G* of a primal (line) graph G is constructed by exchanging edges and vertices.~~ For directed graphs, ~~which are most pertinent here,~~ the vertex set of the line digraph G* corresponds to the edge set of G, and every directed edge in G* corresponds to an instance in G where the head of an edge e_i meets the tail of another edge e_j. **Figure 5** shows a generic example.

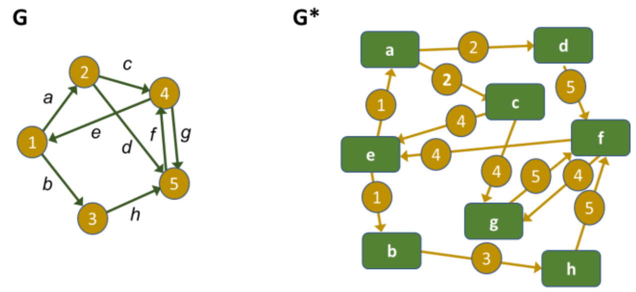

Figure 5. Example of a directed line graph G and its (edge-to-vertex) dual graph G*. The vertices of G* correspond directly to the edges in G, as indicated by letters a – h. The edges of G* correspond to vertices in G if one edge is entering this vertex and another one is leaving it. For instance, vertex 2 in G is represented in the directed dual graph G* through the edges from a to c and from a to d, because these edges meet head to tail at vertex 2 in G.

~~In graph theory, the different types of graph duality have been a topic of discussion for many years (e.g., [85]).~~ In contrast to static graph theory, the literature on dual dynamic models is scattered, with rather different concepts using the term "dual" (*e.g.*, [49-51] ~~[160-162]~~). Closest in spirit to the situation of T&A models is an article on flux duality [52]~~[163]~~, where the exchange of variables and fluxes in certain types of primal and dual differential equation models was investigated. One must emphasize at this point that the notion of line graphs or line digraphs is only an analogy to our situation of T&A models. Nevertheless, this analogy helps us set~~ting~~ up anchors and templates in an appropriate manner, as described below.

Formatted: Indent: First line: 0"

Formatted: Font: Italic

Formatted: Not Highlight

Formatted: Font: Italic

Formatted: Highlight

Formatted: Highlight

A typical anchor model has the underlying graph structure of a common typical metabolic or physiological model. For instance, the variables (vertices) of the cholesterol biosynthesis model (**Figure 3**) are metabolites, and the fluxes (directed edges) among them represent (regulated) biochemical reactions, transport steps, or the formation or disassociation of complexes. This type of diagram represents the biosynthesis anchor well and, as a natural consequence of the typical model design applied here, there is perfect flux balance at any steady state: the fluxes into any of the variables collectively equal the fluxes leaving this variable in magnitude.

Surprisingly, these fundamental features are not necessarily presentdifferent in a template model. The variables (vertices) in a template model are usually physiological or biochemical *processes*, or conglomerations of such processes. In the case of the cholesterol template, for example, the variables are the mevalonate pathway, cholesterol storage, the dynamics various roles of (cholesterol-containing) plasma lipids, and so on (**Figure 2**), which are all processes altering the concentration of free or bound cholesterol. Concomitantly, the directed edges in the template model do not represent material fluxes in the traditional sense. Rather, they indicate modifying effects that may increase or decrease the activity of another template variable, which itself is a process. These effects either consist of handing molecules like cholesterol from one subsystem to another or they represent indirect modulations. Importantly, there is no material flow between template variables in a strict sense, because “Biosynthesis” does not become “Plasma Dynamics,” in analogy to “Lanosterol” becoming “Cholesterol” in the anchor model. Instead, the variable “Diet” increases the amounts of cholesterol in the variables “Plasma Dynamics” and “Storage,” and the variable “Biosynthesis” indirectly affects an increase in the input to the template variable “Sex Hormone Production.” Furthermore, material may be generated or disappear within boxes (template variables). For instance, cholesterol disappears within the box “Sex Hormone Production,” because it is converted into testosterone, estradiol, and other steroids [37]. In Within the template box variable “Biosynthesis”, cholesterol is apparently created out of nothinga source of no concernnothing. Thus, a more accurate, although slightly awkward depiction of the cholesterol template is shown in **Figure 6**. It is of note that this representation actually runs counter to the standards of typical biological diagrams (*e.g.*, [13]). As an For example, consider the signal emitted by a box like “Tissue Usage” on “Storage” within this diagram: It signifies that increased use of cholesterol in the peripheral tissues indirectly affects the storage of cholesterol in the liver, for instance by triggering its release from the storage compartment or by preventing available cholesterol from being stored.

triggers a release of cholesterol from storage in the liver.

Formatted: Font: Italic

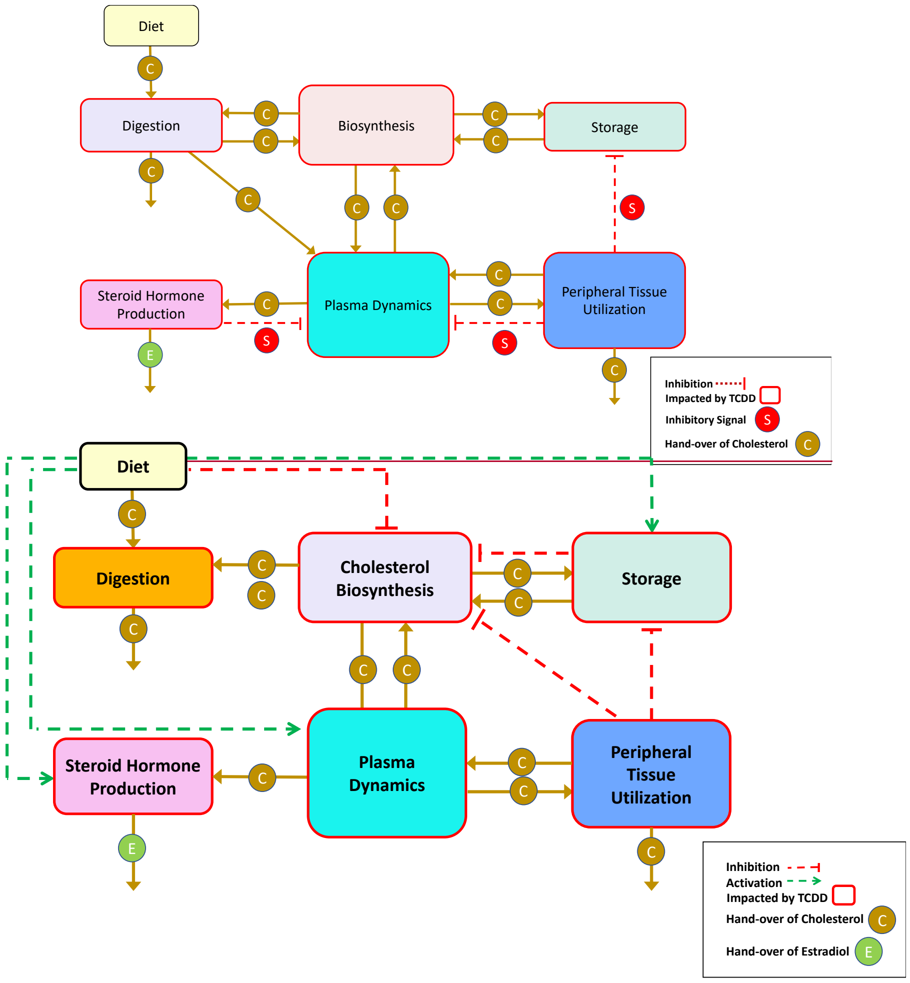

Figure 6. Cholesterol template model as dual graph. New representation of the cholesterol template model, where variables are in truth processes and edges either represent the moving of cholesterol among processes or indicate inhibitory signals . Note that the arrows defy the standards of typical proper model diagrams [13].

The stark difference in model structures has implications for the stacking of sub-models during the zooming between templates and anchors. Specifically, it is possible that "anchors" associated with a template model can be converted into ~~in fact have the structure of~~ lower-level templates. This change comes with a change in model structure. As an

example, “cholesterol storage in the liver” is currently an anchor model that contains as main variable the currently stored amount of cholesterol. However, if this anchor model is to become a template for an underlying model of finer granularity, the variables become the molecular and physiological processes of storing and releasing cholesterol in response to demands. The result could be a sub-template model, with its own anchors, for a lower organizational level.

As ~~an~~ a different example, suppose a high-level template represents some aspect of cellular physiology. The anchors then could represent mitochondrial energy handling, protein synthesis, generation of reactive oxygen species, and a feature like cell contraction. These anchors clearly have the structure of template models, as they represent systems of processes, rather than processes whose variables are biological entities like metabolites. Thus, in typical T&A models, the variables of a template models might be true anchor models, which are usually not further reducible to sub-models, or they are themselves lower-level template models with variables that may either be further templates or true anchors. For some psychological reasons, we tend to think downward, as from a template to anchors. Here, it might be more ~~convenient~~ instructive to start with pure anchors at the lowest level of interest, which become the variables of templates at a higher level, which in turn are integrated in template models at even higher levels until one reaches an over-arching umbrella template.

~~As an~~ To recall an earlier example as yet another ~~example~~illustration, ~~different from our case study,~~ consider again a typical ~~physiologically based pharmacokinetic (~~PBPK~~)~~ model (**Figure 1**). At first glance, the variables appear to be organs, but a ~~slightly misleading~~ moniker like “Liver” is misleading: The variable “Liver” in truth means the concentration of a drug or toxicant being processed by th~~is organ~~e liver at some point in time. The ~~value~~quantity (numerical value) of this variable is not only driven by the influx of the drug from the arterial and portal blood ~~-~~and its efflux into the venous blood but also by the enzymatic, transport, and storage activities that alter ~~(*e.g.*, bind, degrade or excrete) some~~ a portion of this drug within the organ by means ofdegradation, excretion, or binding. Without accounting for th~~is enzymatic activity or excretion~~ese processes within the given box (*i.e.*, variable), there is no valid flux balance at a steady state. This situation is reconciled if one recognizes that the variable “Liver” represents a system of processes in which the ~~overall~~ observed influx is converted into the ~~overall~~ observed efflux. ~~In other words~~Thus, this situation of a PBPK model is typical for a template model, whose anchor “Liver” is a lower-level template that contains subsystems of ~~biosynthesis~~uptake, degradation, excretion, ~~or~~ storage, and other processes. At the same time, each apparent flux (edge) in the PBPK model in truth represents the concentration of the drug or toxicant being transported from one organ to another. As yet a different example, we recently proposed a template model for cystic fibrosis, where the variables at first glance ~~are~~ appeared to be organs (*e.g.*, intestine), but in reality consisted of processes such as digestion [53].

By the same token, a template may become an anchor within a higher-level template model. In our example of the effect of dioxin on cholesterol handing, one could imagine that our current template might serve as an anchor in a higher-level model that investigates the effects of dioxin on the entire organism and contains additional physiological systems, such as the skin, or assesses dioxin-related carcinogenesis. Clearly, these effects are not independent from cholesterol handling, which we discuss here, and our current template could serve as an anchor within an overall model of dioxin effects in parallel to other anchors.

As a higher-level example, one could imagine a template describing the spread of an infection (with a structure like a sophisticated, detailed Susceptible-Infected-Recovered-Susceptible (SIRS) ~~SIRS~~ model, maybe for an age-stratified population). The anchors could be individuals who interact and thereby spread the disease. Each individual would be characterized by her/his social interactions with others and the potential of being infected. For each individual, one might model the inhalation processes and the immune system with individualized properties, which would determine the effectiveness of being infected, fighting the infection through a strong immune response and the concomitant

Formatted: Font: Italic

transmission potential of the disease. Under the same template, it could also be possible to create anchor models of bacteria or viruses causing the disease. Clearly, there are interactions between the pathogens and the individuals' immune systems and, thus among the anchors at this level. At a more granular level, these anchors could be templates for underlying physiological or molecular systems.

~~Taken together,~~To summarize this novel and initially confusing aspect of model structures, anchors have the ~~structure~~ format of typical biomathematical models. Their vertices~~constituents~~ are pools (~~(vertices,~~ *e.g.*, organic compounds~~)~~) and their edges are fluxes, visualized as arrows that, ~~(edges;~~ *e.g.*, represent enzymatic reactions~~,~~ or transport processes~~)~~. By contrast, typical template models have the structure of dual systems: The vertices are processes~~,~~ and the edges are entities like chemical compounds or signals. In the relationship between templates and anchors, the variables of templates ~~variables~~ become anchor~~models~~s. These anchors may be true, elementry anchors or turn out to be ~~new,~~ lower-level template models. A true anchor, like "Biosynthesis" in Figure 2, contains variables that are molecules (*e.g.*, lanosterol), which are handed off from one vertex to the next. Its processes are fluxes in the truest sense, as lanosterol is converted into—and thus becomes—cholesterol. However, if an anchor is a new template at a lower level, its variables are again processes or systems of processes. The edges of a template model, whether at the highest or a lower level, consist of signals or ~~of~~ entities (molecules) that are handed from one anchor to the next. Thus, the multiscale organization effort is complete once all anchor models are true anchors with variables that are entities rather than processes.

There are bound to be exceptions to this generalization of the T&A structure, but the feature of duality in typical T&A models highlights the fundamental difference between anchors and templates ~~and anchors~~. It is unclear whether the recognition of this duality has practical ramifications beyond academic insights and rules for setting up the different models, but it does demonstrate that the two model types in T&A structures contain intrinsic differences. These differences offer guideposts for an effective hierarchical model design that spans different levels.

Discussion

The T&A modeling approach, originally suggested over two decades ago, was used for the purpose of inferring a set of common design concepts of posture and movement from different modes of locomotion among terrestrial species [31]. The authors pursued this goal by creating a model on a conceptual low-dimensional manifold describing the key aspects of locomotion. This invariant manifold was conceived as being embedded within a much-higher-dimensional space describing the various observed modes of locomotion, and all species-specific implementations were seen as intersecting in the subspace of the common design [31, 32, 54].

We propose in this article the T&A paradigm for a different purpose, namely for analyzing multiscale biomedical models in an effective manner. As demonstrated here and in the companion article, the approach provides a systematic and intuitively organized strategy for multi-level analysis of complex biological systems. The template model serves as a high-level, coarse-grained representation that focuses on the main physiological components of a system and entails a ~~minimal~~ relatively small set of variables and parameters. It acts as an overarching view of the entire ~~system~~problem space, thus providing an overall snapshot of the dynamic interactions among its core constituents. By contrast, the anchor models provide a more detailed and finely grained view of specific biological details and mechanisms within the system. They allow the user to zoom into individual variables within the template, thereby permitting an account of intricate aspects of the underlying processes. Together, the template and anchor models form a hierarchical structure, allowing for both broad overviews and in-depth analyses, thereby offering a versatile tool for multiscale modeling.

Formatted: Indent: First line: 0"

As an illustration of the implementation of the T&A model, we describe the impact of dioxin exposure on hepatic cholesterol levels. Diverse simulation scenarios can be performed, such as different exposure scenarios, dietary cholesterol intake regimens, and ~~the~~ varied combinations~~-ed effect of dietary cholesterol variation with dioxin exposure on~~ to predict their effects on the hepatic and plasma cholesterol levels [37]. In this example, the dose-response relationship between dioxin exposure and the corresponding hepatic cholesterol concentration is the main output that ~~will~~ subsequently informs the template model and other anchor models. The technical intricacies of the T&A framework are discussed in the companion paper [1].

The T&A modeling approach carries distinct advantages in handling multiscale problems. In particular, the method enhances computational efficiency through a divide-and-conquer strategy and provides a flexible structure for model setup and the definition of variables across different levels. In contrast to other multiscale approaches, each anchor is separately analyzed, yielding a comprehensive record of essential input-output relationships that inform other anchors and calibrate the template model, while allowing for the interchangeability of anchor models without affecting the overall template structure.

Of course, the T&A approach is no panacea, and our dioxin exposure model, for example, is obviously simplified, like any other model. Even for the single anchor discussed here, the mevalonate pathway, much more ~~sophistication~~ detail could—and maybe should—be considered. The same is true for other anchors, such as our model of steroidogenesis in the companion article. For instance, it has been demonstrated that gene expression in the cholesterol biosynthetic pathway differs among hepatocytes depending on where they are located within the ~~micro~~ lobules, a microstructure- of the liver [34, 55, 56]. Also, we performed our analysis at the steady state of the pathway system, which may seem restrictive. However, it is expected that the ~~does~~dose-response curve we determined with the anchor model is similarly applicable to non-steady-state situations and that the pathway within the human body operates relatively close to the steady state. Despite these limitations, the proposed approach appears to be sufficient for revealing the essence of a complex phenomenon, as in our example the impact of dioxin on cholesterol biosynthesis. A different caveat is that even the best model is only as good as the supporting data, and the data need for a complete T&A analysis may greatly exceed reality. In our example, the amount of human data is scarce and our supplanting them with mouse data raises clear uncertainties. At a theoretical level, the duality between templates and anchors will require a deeper investigation and an exploration of its speculated generality.

Overall, multiscale models are clearly important, and the T&A approach is intended to provide a novel, effective tool for complex modeling tasks as it provides a comprehensive and holistic perspective that accommodates a range of organizational scales. It is quite evident that an approach like T&A can readily be tailored toward many different research goals and applications, which could, for instance, support traditionally difficult extrapolations of *in vitro* to *in vivo* results [57] and the important translation of observations between different species [58]. For our example of ~~TCDD~~ dioxin exposure, this translation poses an interesting challenge, as some enzyme activities and the crucial ~~TCDD~~dioxin-AhR mechanism are species-specific [59, 60].

~~Looking ahead~~As one future application, the T&A framework holds potential for advancing personalized risk assessments and personalized medicine [42, 43] in general. ~~By the same token, it~~It could also become relevant and effective for enhancing drug discovery and development. The current version of the model is mostly calibrated with average data, but also indicates how variability may be taken into account. We illustrate this aspect in the companion paper~~–~~, ~~considers~~with the example of inter-~~personal~~individual variability~~population variability, for instance,~~ in menstrual cycle length within a population, ~~whe~~where the production of estradiol is affected by~~n modeling the~~ dioxin ~~mediated effects on estradiol , as detailed in our companion paper~~.~~–~~ For future uses in personalized medicine, the model design ~~would have to be~~is readily customizable, according to published personalization methods [61, 62] ~~[174, 175]~~ that are based ~~allowing for the easy~~ on

Formatted: Not Highlight

Formatted: No underline, Font color: Auto

Formatted: No underline, Font color: Auto

Formatted: No underline, Font color: Auto

Formatted: No underline, Font color: Auto

Formatted: No underline, Font color: Auto

Formatted: No underline, Font color: Auto

Formatted: No underline, Font color: Auto

Formatted: No underline, Font color: Auto

individualized adjustments of relevant parameters based and thus on personalized data [174, 175]. As an example in this direction, tThe proposed T&A strategy indicates was employed to investigatehow to study the impact of simultaneous external inputs such as dietary cholesterol intake under exposure to and environmental toxins, notably dioxin [37]. These examples renferrender it evident thatObviously, the approach itself is independent of this application and it is quite evident that the T&A platform is versatile and can be expanded further in various directions to simulate the body's response to a wide array of pharmaceutical and toxic compounds, thereby potentially serving as a predictive tool for assessing drug the behaviors and interactions of drugs and environmental exposures within the body [63][176].

Formatted: No underline, Font color: Auto

Formatted: No underline, Font color: Auto

Formatted: No underline, Font color: Auto

Formatted: No underline, Font color: Auto

Formatted: No underline, Font color: Auto

Formatted: No underline, Font color: Auto

Code Availability

Modeling details and computer code can be found in the supplements of the companion article [1] [ ] and on GitHub GitHub.com/LBSA-VoitLab/TCDD_Chol######. Submission to Biomodels will follow after acceptance of the article.

Formatted: Indent: First line: 0"

Formatted: Not Highlight

Formatted: Not Highlight

Acknowledgments: This work was supported in part by NIEHS HERCULES grant P30ES019776-05 (PI: Carmen Marsit) and NIEHS Superfund Research grant P42ES04911 (PI: Norbert Kaminski). The funding agency is not responsible for the content of this article.

References

1. Kumbale, C.M., E.O. Voit, and Q. Zhang, *Multiscale Biomedical Systems Analysis through Template-and-Anchor Modeling 2. Application to Cholesterol Handling and the Effects of Dioxin.* 2025.
2. Schnell, S., R. Grima, and P. Maini, *Multiscale Modeling in Biology.* American Scientist - AMER SCI, 2007. **95**.
3. Savageau, M.A., *The challenge of reconstruction.* New Biol, 1991. **3**(2): p. 101-2.
4. Savageau, M.A., *Reconstructionist molecular biology.* New Biol, 1991. **3**(2): p. 190-7.
5. Kreeger, P.K. and D.A. Lauffenburger, *Cancer systems biology: a network modeling perspective.* Carcinogenesis, 2009. **31**(1): p. 2-8.
6. Anderson, A.R. and V. Quaranta, *Integrative mathematical oncology.* Nat Rev Cancer, 2008. **8**(3): p. 227-34.
7. Shurin, M.R., *Cancer as an immune-mediated disease.* Immunotargets Ther, 2012. **1**: p. 1-6.
8. Goetz, L.H. and N.J. Schork, *Personalized medicine: motivation, challenges, and progress.* Fertil Steril, 2018. **109**(6): p. 952-963.
9. Millar-Wilson, A., et al., *Multiscale modeling in the framework of biological systems and its potential for spaceflight biology studies.* iScience, 2022. **25**(11): p. 105421.
10. Nersessian, N.J., *Interdisciplinarity in the Making: Models and Methods in Frontier Science.* 20222: MIT Press.
11. Baumer, B.S., D.T. Kaplan, and N.J. Horton, *Modern Data Science with R (2nd Ed.* 2021: CRC Press.
12. Voit, E.O., *The Inner Workings of Life: Vignettes in Systems Biology*. 2016: Cambridge University Press.
13. Voit, E., *A First Course in Systems Biology (2nd ed.). Garland Science*. 2017.
14. Liu, R., et al., *A Physiologically Based Pharmacokinetic (PBPK) Modeling Framework for Mixtures of Dioxin-like Compounds.* Toxics, 2022. **10**(11).
15. Wilensky, U. and W. Rand, *An Introduction to Agent-Based Modeling Modeling Natural, Social, and Engineered Complex Systems with NetLogo*. 2015: The MIT Press.
16. Versypt, A.N.F., *Multiscale modeling in disease.* Curr Opin Syst Biol, 2021. **27**.
17. Glen, C.M., M.L. Kemp, and E.O. Voit, *Agent-based modeling of morphogenetic systems: Advantages and challenges.* PLOS Computational Biology, 2019. **15**(3): p. e1006577.

18. Kasereka, S., N. Kasoro, and P. Chokki, *A hybrid model for modeling the spread of epidemics: Theory and simulation*. 2014.
19. Bardini, R., et al., *Multi-level and hybrid modelling approaches for systems biology.* Comput Struct Biotechnol J, 2017. **15**: p. 396-402.
20. Garbey, M., S. Casarin, and S.A. Berceli, *A versatile hybrid agent-based, particle and partial differential equations method to analyze vascular adaptation.* Biomechanics and Modeling in Mechanobiology, 2019. **18**(1): p. 29-44.
21. Mu, D.P., et al., *A multiscale spatial modeling framework for the germinal center response.* Front Immunol, 2024. **15**: p. 1377303.
22. Cruz, D.A. and M.L. Kemp, *Hybrid computational modeling methods for systems biology.* Progress in Biomedical Engineering, 2022. **4**(1): p. 012002.
23. Cassidy, R., et al., *Mathematical modelling for health systems research: a systematic review of system dynamics and agent-based models.* BMC Health Services Research, 2019. **19**(1): p. 845.
24. Lättilä, L., P. Hilletofth, and B. Lin, *Hybrid simulation models – When, Why, How?* Expert Systems with Applications, 2010. **37**(12): p. 7969-7975.
25. Bodine, E.N., et al., *Agent-Based Modeling and Simulation in Mathematics and Biology Education.* Bulletin of Mathematical Biology, 2020. **82**(8): p. 101.
26. Liao, J.C. and E.N. Lightfoot, Jr., *Lumping analysis of biochemical reaction systems with time scale separation.* Biotechnol Bioeng, 1988. **31**(8): p. 869-79.
27. Savageau, M.A., *Growth of complex systems can be related to the properties of their underlying determinants.* Proceedings of the National Academy of Sciences, 1979. **76**(11): p. 5413-5417.
28. Gunawardena, J., *Time-scale separation--Michaelis and Menten's old idea, still bearing fruit.* Febs j, 2014. **281**(2): p. 473-88.
29. Ferreira, A.E.N., A.M. Ponces Freire, and E.O. Voit, *A quantitative model of the generation of N(epsilon)-(carboxymethyl)lysine in the Maillard reaction between collagen and glucose.* Biochem J, 2003. **376**(Pt 1): p. 109-21.
30. Collier, M.L., et al., *Calcium-induced calcium release in smooth muscle: loose coupling between the action potential and calcium release.* J Gen Physiol, 2000. **115**(5): p. 653-62.
31. Full, R.J. and D.E. Koditschek, *Templates and anchors: neuromechanical hypotheses of legged locomotion on land.* J Exp Biol, 1999. **202**(Pt 23): p. 3325-32.
32. Kvalheim, M., and Revzen, S, *Templates and Anchors; in M.A*. Bioinspired Legged Locomotion. Models, Concepts, Control and Applications, ed. M. Sharbafi and A. Seyfarth. 2017, Oxford, UK.
33. Angrish, M.M., C.Y. Dominici, and T.R. Zacharewski, *TCDD-elicited effects on liver, serum, and adipose lipid composition in C57BL/6 mice.* Toxicol Sci, 2013. **131**(1): p. 108-15.
34. Fader, K.A., et al., *2,3,7,8-Tetrachlorodibenzo-p-Dioxin Alters Lipid Metabolism and Depletes Immune Cell Populations in the Jejunum of C57BL/6 Mice.* Toxicol Sci, 2015. **148**(2): p. 567-80.
35. Institute of Medicine Committee to Review the Evidence Regarding the Link Between Exposure to Agent, O.D., in *Veterans and Agent Orange: Herbicide/Dioxin Exposure and Type 2 Diabetes*. 2000, National Academies Press (US) Copyright 2000 by the National Academy of Sciences. All rights reserved.: Washington (DC).
36. Tuomisto, J.T., et al., *Soft-tissue sarcoma and dioxin: A case-control study.* Int J Cancer, 2004. **108**(6): p. 893-900.

37. Kumbale, C.M., Q. Zhang, and E.O. Voit, *Hepatic cholesterol biosynthesis and dioxin-induced dysregulation: A multiscale computational approach.* Food and Chemical Toxicology, 2023: p. 114086.
38. Greene, J.F., S. Hays, and D. Paustenbach, *Basis for a proposed reference dose (RfD) for dioxin of 1-10 pg/kg-day: a weight of evidence evaluation of the human and animal studies.* J Toxicol Environ Health B Crit Rev, 2003. **6**(2): p. 115-59.
39. Voit, E.O., *The best models of metabolism.* Wiley Interdiscip Rev Syst Biol Med, 2017. **9**(6).
40. Voit, E.O., *Computational Analysis of Biochemical Systems: A Practical Guide for Biochemists and Molecular Biologists*. 2000: Cambridge University Press.
41. Voit, E.O., *Biochemical Systems Theory: A Review.* ISRN Biomathematics, 2013. **2013**: p. 897658.
42. Voit, E.O. and K.L. Brigham, *The Role of Systems Biology in Predictive Health and Personalized Medicine.* The Open Pathology Journal, 2008. **2**: p. 68-70.
43. Davis, J.D., et al., *Dynamical systems approaches to personalized medicine.* Curr Opin Biotechnol, 2019. **58**: p. 168-174.
44. Kumbale, C.M. and E.O. Voit, *Toward Personalized Medicine for HIV/AIDS.* J AIDS HIV Treat, 2021. **3**(2): p. 37-41.
45. Malhotra, P., et al., *Disturbances in Cholesterol Homeostasis and Non-alcoholic Fatty Liver Diseases.* Front Med (Lausanne), 2020. **7**: p. 467.
46. Whitney, H., *A logical expansion in mathematics.* Bulletin of the American Mathematical Society, 1932. **38**(8): p. 572-579, 8.
47. Harary, F. and R.Z. Norman, *Some properties of line digraphs.* Rendiconti del Circolo Matematico di Palermo, 1960. **9**(2): p. 161-168.
48. Beineke, L.W. and R.L. Hemminger, *Infinite digraphs isomorphic with their line digraphs.* Journal of Combinatorial Theory, Series B, 1976. **21**(3): p. 245-256.
49. Dassios, I. and F. Milano, *Singular dual systems of fractional ‐order differential equations.* Mathematical Methods in the Applied Sciences, 2021.
50. Rudolph, J., *Duality in time-varying linear systems: A module theoretic approach.* Linear Algebra and its Applications, 1996. **245**: p. 83-106.
51. Bruce, J.W. and F. Tari, *Duality and implicit differential equations.* Nonlinearity, 2000. **13**(3): p. 791.
52. Marin-Sanguino, A., E.R. Mendoza, and E.O. Voit, *Flux duality in nonlinear GMA systems: implications for metabolic engineering.* J Biotechnol, 2010. **149**(3): p. 166-72.
53. Olivença, D.V., et al., *Mathematical models of cystic fibrosis as a systemic disease.* WIREs Mech Dis, 2023: p. e1625.
54. Holmes, P., et al., *The Dynamics of Legged Locomotion: Models, Analyses, and Challenges.* SIAM Review, 2006. **48**(2): p. 207-304.
55. Nault, R., et al., *Dose-Dependent Metabolic Reprogramming and Differential Gene Expression in TCDD-Elicited Hepatic Fibrosis.* Toxicol Sci, 2016. **154**(2): p. 253-266.
56. Nault, R., et al., *Lipidomic Evaluation of Aryl Hydrocarbon Receptor-Mediated Hepatic Steatosis in Male and Female Mice Elicited by 2,3,7,8-Tetrachlorodibenzo-p-dioxin.* Chem Res Toxicol, 2017. **30**(4): p. 1060-1075.
57. Zhang, Q., et al., *Bridging the Data Gap From in vitro Toxicity Testing to Chemical Safety Assessment Through Computational Modeling.* Frontiers in Public Health, 2018. **6**.
58. Brubaker, D.K., et al., *An interspecies translation model implicates integrin signaling in infliximab-resistant inflammatory bowel disease.* Sci Signal, 2020. **13**(643).

59. Schulz, T.G., et al., *Inducibility of cytochromes P-450 by dioxin in liver and extrahepatic tissues of the marmoset monkey (Callithrix jacchus).* Biochimica et Biophysica Acta (BBA) - Protein Structure and Molecular Enzymology, 1996. **1298**(1): p. 131-140.
60. Fader, K.A. and T.R. Zacharewski, *Beyond the Aryl Hydrocarbon Receptor: Pathway Interactions in the Hepatotoxicity of 2,3,7,8-Tetrachlorodibenzo-p-dioxin and Related Compounds.* Curr Opin Toxicol, 2017. **2**: p. 36-41.
61. Chen, R. and M. Snyder, *Systems biology: personalized medicine for the future?* Curr Opin Pharmacol, 2012. **12**(5): p. 623-8.
62. Prague, M., D. Commenges, and R. Thiebaut, *Dynamical models of biomarkers and clinical progression for personalized medicine: the HIV context.* Adv Drug Deliv Rev, 2013. **65**(7): p. 954-65.
63. Schoeberl, B., *Quantitative Systems Pharmacology models as a key to translational medicine.* Current Opinion in Systems Biology, 2019. **16**: p. 25-31.
