# Supplementary material for: Analysis of systemic effects of dioxin on human health through template-and-anchor modeling
Source: PLoS Comput Biol. 2025 Mar 27;21(3):e1012840. doi: 10.1371/journal.pcbi.1012840 (PMC12005559; doi:10.1371/journal.pcbi.1012840)
Supplement: Attachment — Submitted filename: Rebuttal 111.docx [file pcbi.1012840.s004.docx]

Dear Professor McCulloch:

Thank you for the constructive reviewer comments and the opportunity to revise our work. While we are responding to the reviewer comments one-by-one below, the major aspect of the revision is that we have followed the first reviewer’s suggestion of creating an accompanying article that contains a comprehensive illustration of how a template-and-anchor (T&A) model is to be set up and analyzed. Importantly, it makes it clearer how information flows between the two levels. We are confident that this substantial change is a true improvement and hope that you and the reviewers will agree with our decision. In addition, we have made substantial editions to the present (first) paper, taking into consideration the constructive comments made by the reviewers.

Below, please find itemized responses to all specific comments and suggestions by the reviewers.

In addition to the two manuscript, we are submitting the original manuscript with Tracker changes for the reviewers’ perusal.

**Responses to the Reviewers’ Comments**

**Reviewer #1**

The manuscript presents a complex set of ideas with potential, but several questions arose as I finished reading it, mainly regarding clarity in exposition and organization.

Response: Thank you for the encouragement. In this revision, we have tried to reduce ambiguities in our description of the method.

While the paper introduces the Template and Anchor (T&A) approach for multiscale modeling, the conceptual framework of T&A modeling remains somewhat unclear. The distinction between template and anchor models seems inconsistently articulated throughout the paper, potentially causing confusion for both myself and readers unfamiliar with the concept. A clearer definition and differentiation between template and anchor models in the introduction section could help, but may not sufficiently elucidate the adaptation from biomechanics to biomolecular levels.

Response: Thank you for the suggestions. We have devoted substantial effort to clarifying concepts and methodological details throughout the manuscript. As part of this rewriting, we have amended the introduction by adding clarifying verbiage. Importantly, following your suggestion, we have created an entirely new companion article with a detailed example of a T&A analysis of dioxin-mediated effects on cholesterol dynamics and steroidogenesis. This example discusses all steps required for the set-up of the template model and of various anchor models and then illustrates how information is transmitted from the anchors to the template model. We refer to this example in the introduction and in other locations of the first article.

The transition from a mechanistic-like model to a graph-theoretical model needs clearer explanation.

Response: There is not really a transition, as the mechanistic model is not directly affected by the graph-theoretical model or *vice versa*. Instead, the reference to graph theory is purely an analogy, which may help some readers see the intriguing differences between template and anchor models. We have attempted to make this analogy clearer.

Duality of graphs is evoked only to demonstrate that the template model and the anchor models have a different character. In particular, a conventional model, including a typical anchor model, contains variables that represent molecules or other physical quantities. By contrast, we argue, that a template model contains variables that do not represent molecules or physical quantities but processes. This insight has consequences for setting up the different models and their constituents. Having said that, we propose that anchor models may serve as template models at lower levels. In this case, the character of the model changes. As an example, “cholesterol storage in the liver” is currently an anchor model that contains as main variable the currently stored amount of cholesterol. However, if this anchor model is to become a template for an underlying model of finer granularity, the variables become the molecular and physiological processes of storing and releasing cholesterol in response to demands. The result could be a template model, with its own anchors, for a lower organizational level.

In addition to amending the text, we refer to the companion paper, which describes the design and analysis of both model types and the conceptual interactions between them. We also provide additional references to papers on graph duality that are considered the first to discuss duality of graphs. Indeed, this topic has been discussed in the literature for a long time, using different terms, such as “line graphs,” “edge graphs” and “edge-to-vertex duals,” among others. Line graphs were arguably first mentioned by Hassler Whitney in 1931/1932. A good review is the 1972 book by Harary and, in particular, the chapter on line graphs within this book. In our case, the specific targets are actually directed graphs (“digraphs”) and the corresponding “line digraphs,” which were discussed in Harary and Norman (1960) and Hemminger and Beineke (1978).

Whitney, H. (1931) Non-separable and planar graphs. *PNAS* **17**, 125-127.

Whitney, H. (1932) Congruent Graphs and the Connectivity of Graphs. *American Journal of Mathematics* **54 (1)**, 150-168.

Harary, F. (1972) *Graph Theory*, Massachusetts: Addison-Wesley.

Harary, F., Norman, R.Z. (1960) Some properties of line digraphs. *Rend. Circ. Mat. Palermo* **9**, 161–168. <https://doi.org/10.1007/BF02854581>

Hemminger, R. L.; Beineke, L. W. (1978), "Line graphs and line digraphs", in Beineke, L. W.; Wilson, R. J., *Selected Topics in Graph Theory,* Academic Press Inc., pp. 271–305.

The current structure of the explanation gives the impression of a two-level modeling approach, rather than a multilevel one. Did I misunderstand this? For instance, how would one use the methodology to progress from molecular networks to tissues, organisms, and populations/epidemiological groups? How would more than two levels be addressed?

Response: It is true that the example we are analyzing primarily spans two levels. However, as we point out in the theoretical discussion, it is possible that an anchor model, *mutatis mutandis*, might in fact serve as the template model for lower-level anchors. By the same token, a template could become an anchor within a higher-level template model. In our example of the effect of dioxin on cholesterol handing, one could imagine that our template might serve as an anchor in a higher-level model that investigates the effects of dioxin on the entire organism and contains additional physiological systems, such as the skin, or assesses dioxin-related carcinogenesis. Clearly, these effects are not independent from cholesterol handling, which we discuss here, and our current template could serve as an anchor within an overall model of dioxin effects in parallel to other anchors.

At this point, our example in the companion article discusses the progression from molecular systems, such as metabolic pathways, to tissues and physiological systems, such as the liver and lipoprotein dynamics in the plasma. It also touches on reproductive dysfunction caused by dioxin, which clearly is a higher-level issue.

Nonetheless, the reviewer’s point is well taken. As detailed in the response to the previous comment, our current template could serve as an anchor in a higher-level template that might not only look at dioxin-induced cholesterol dysfunction but also at effects on other aspects of the organism, such as tumor formation. In terms of population-based phenomena, we mention the use of epidemiological data in the anchor of steroidogenesis. As a more generic example, one could imagine a template describing the spread of an infection (with a structure like a sophisticated, detailed SIRS model, maybe for an age-stratified population). The anchors could be individuals who interact and thereby spread the disease. Each individual would be characterized by her/his social interactions with others and the potential of being infected. For each individual, one might model the inhalation processes and the immune system with individualized properties, which would determine the effectiveness of being infected, fighting the infection through a strong immune response and the concomitant transmission potential of the disease. Under the same template, it could also be possible to create anchor models of bacteria or viruses causing the disease. Clearly, there are interactions between the pathogens and the individuals’ immune systems and, thus among the anchors at this level. At a more granular level, these anchors could be templates for underlying physiological or molecular systems.

We are sorry if all this sounds a bit vague, but the use of Full and Koditchek’s original T&A approach proposed here is new and so far untested in terms of possibilities and limitations.

While the paper discusses practical applications of the T&A approach, such as personalized medicine and translational research, it lacks specificity in describing how the approach can be applied in real-world scenarios. While the example presented throughout the paper is helpful, it is insufficient to fully illustrate the complex concepts. There are two way I can see to improve this. One is by creating a set of sections or a detailed methodology (or a second accompanying manuscript) where the concepts are properly formalized. The alternative, is providing more examples with similar depth to the one you use to enhance readers' understanding of the underlying concepts. Highlight specific challenges addressed and insights gained through T&A modeling in diverse contexts.

Response: Thank you for this suggestion, which we have wholeheartedly accepted. The new accompanying article presents a very detailed example that illustrates model design and analysis. We have shown elsewhere [Voit and Brigham, 2008; Davis *et al.*, 2019] how population-based models may be personalized, and the T&A approach could be personalized in a conceptually straightforward manner (even though substantial information would be needed for such an implementation). Nonetheless, the structure of the T&A approach is no hindrance toward personalization. As an indication in this direction, we previously studied interactions between dioxin exposure and dietary cholesterol uptake [Kumbale *et al.*, 2023].

We also mention that the well-known physiologically-based pharmacokinetic (PBPK) models can be seen as simple T&A models.

We have added a section to the discussion on how the T&A framework can be effectively utilized in personalized medicine and for drug discovery.

Voit, E. O. and K. L. Brigham: The role of systems biology in predictive health and personalized medicine. *The Open Path. J.* **2**, 68-70, 2008.

Davis, J.D., Kumbale, C.M., Zhang, Q. and E.O. Voit: Dynamical systems approaches to personalized medicine. *Curr. Opin. Biotechn.* **58**, 168-174, 2019.

Kumbale, C.M., Q. Zhang, and E.O. Voit: Hepatic cholesterol biosynthesis and dioxin-induced dysregulation: A multiscale computational approach. *Food Chem. Toxicol.* **181**:114086, 2023. doi: 10.1016/j.fct.2023.114086.

Additionally, while the paper acknowledges potential limitations and challenges associated with T&A modeling, the discussion remains relatively brief and lacks depth. Expanding the discussion section to include a more comprehensive analysis of the limitations and challenges of T&A modeling would be beneficial. This could be done by including additional examples that are unrelated to the system that is used throughout the paper.

More detailed sections regarding the limitations for each of our developed anchor models and the overall template model have been added to the discussion of the present paper and in the companion paper.

The paper emphasizes the importance of empirical validation for T&A models but falls short of providing concrete guidance on validation methodologies and best practices. Utilizing more than one example to illustrate the framework and validate its models may facilitate this. The paper could also benefit from a more comprehensive analysis of potential avenues for advancing T&A modeling in biomedical research.

Model validation is always a challenge. Here, the anchors and the template are well structured and thus provides avenues toward individual and overall validation. In terms of anchors, validation and best practices following traditional guidelines that have been governing mathematical modeling for a long time. For instance, the model should be assured to be stable and sufficiently insensitive at the nominal operating point to withstand moderate external perturbations. Also, it is in principle possible to measure details, *e.g.*, for the cholesterol biosynthesis model, with experimental means. Given the paucity of experimental and clinical data, much of the available information was used to implement the model, but other details could be measured and compared with model results. In terms of the template model, one could pursue measurements, for instance, targeting the combination of dioxin exposure and different amounts of dietary cholesterol. Similarly, model results regarding steroidogenesis could be validated in principle.

There are some additional comments and typo that you can find in the attached pdf.

Response: Thank you for your careful reading and suggestions. We have taken care of all issues mentioned.

**Reviewer #2**

The manuscript revisits the Template-and-Anchor (T&A) modeling framework, originally proposed for design concepts from various manifestations of biological locomotion. The manuscript emphasizes the T&A framework's utility in simplifying complex multiscale biomedical models by separating them into high-level template models and more detailed anchor models. The template model describes overarching bioprocess components with minimal variables, while anchor models delve into specific biological details of subsystems. This separation enhances computational efficiency, provides a structured approach to model development, and maintains flexibility by allowing anchor models to be independently updated or replaced. The manuscript illustrates this framework's application in the modeling of dioxin exposure and its effects on cholesterol dynamics, showcasing its potential.

The manuscript's exploration of the Template-and-Anchor (T&A) framework, particularly its application to biological systems modeling, is commendable. The presentation of the T&A framework is insightful, but its application to dioxin exposure needs clarifications (see Major Comments). I believe this work will likely be well-received by practitioners of biosystems modeling.

Response: Thank you for these encouraging comments.

Major comments:

1. The manuscript provides a general description of the Template-and-Anchor (T&A) framework. Unfortunately, it lacks detailed implementation specifics, particularly in the dioxin exposure case study. Essential elements such as the interaction between template and anchor models, specific equations governing the dioxin model, and the nature of information exchanged between template and anchor are notably absent. This omission hampers the reader's ability to fully understand and appreciate the model's complexity and operational mechanisms. For example, it is not clear how one would get the simulation result in Fig. 4 since no information of the model that was solved was provided.

Response: We hope that the extensive rewriting of the paper and the addition of the companion paper, as recommended by the first reviewer, offer sufficient methodological detail to support our claims. In particular, we have put much more emphasis outlining how the anchors and templates “interact” with each other. Also, we are now providing code on GitHub and in the Supplements of the companion paper that shows pertinent details.

2. Despite the mention of future code availability on GitHub, the current lack of access to the model's code restricts the ability to replicate the study and/or delve into the technical intricacies of the framework. The absence of code, coupled with the lack of mathematical detail and algorithmic descriptions in the manuscript, significantly detracts from the research's reproducibility, thus limiting its utility and impact within the scientific community.

Response: We are sorry that the previous version did not permit a reproduction of our results and are now providing code on GitHub and in the Supplements of the companion article.

3. The similarity between the Template-and-Anchor (T&A) framework and block diagrams in process control, particularly in how both describe input-output relationships through modular components, deserves a comparison. Like T&A template, block diagrams use blocks to represent the relationships between variables, where such relationships are modeled using transfer functions. The anchors could be seen as complex versions of block diagram transfer functions. A more explicit comparison highlighting the T&A framework's unique contributions and advantages in modeling biological systems, beyond what traditional block diagrams offer, would greatly enhance the manuscript's impact.

Response: Thank you for the insightful comment. In response, we have added new verbiage to the text.

In essentially all our modeling efforts, the first step is the construction of a block diagram or, as we often call it, a box-and-arrow diagram [Voit, 2000, 2017]. We consider this structuring and visualization of a biomedical problem as absolutely essential because, in a way, it replaces reality during the mathematical modeling and analysis process; for a somewhat philosophical account of this facet, please see [Voit, 2019].

While diagrams are crucial, they only go so far, because it is necessary to “bring them to life” by defining what exactly the boxes and the arrows mean. In the case of T&A models, this definition is very different, as the boxes (variables of the template model) turn out to be entire systems (anchors) at a different level of granularity. Thus, we most certainly agree that the first step is a block (box-and-arrow) diagram, but that the proposed approach deals with boxes that are more complex than in typical control theory. We have added verbiage to the text debating these issues.

Voit, E.O.: *Computational Analysis of Biochemical Systems. A Practical Guide for Biochemists and Molecular Biologists*, xii + 530 pp., Cambridge University Press, Cambridge, U.K., 2000.

Voit, E.O.: *A First Course in Systems Biology*. Second Edition, Garland Science, New York, NY, 2017.

Voit, E.O.: Perspective: Dimensions of the Scientific Method. *PLoS Comp. Biol.* **15(9)**: e1007279, 2019.

**Have the authors made all data and (if applicable) computational code underlying the findings in their manuscript fully available?**

Reviewer #1: **No:** Not yet. they say they will provide the code on GitHUb. I would not accept the paper before a link to the repository is provided.

Reviewer #2: **No:** The code generating the model simulations was not provided with the manuscript for review.

Response: We are now providing code on GitHub and in the Supplements of the companion paper.
