# Supplementary material for: Analysis of systemic effects of dioxin on human health through template-and-anchor modeling
Source: PLoS Comput Biol. 2025 Mar 27;21(3):e1012840. doi: 10.1371/journal.pcbi.1012840 (PMC12005559; doi:10.1371/journal.pcbi.1012840)
Supplement: Attachment — Submitted filename: Rebuttal EOV.docx [file pcbi.1012840.s007.docx]

**Responses to Reviewers of the Latest Revision**

Dear Drs. Mendes, Gabhann and Papin:

Thank you for your encouraging feedback on the previous revision of our article. We have followed the reviewers’ suggestions and further amended the article correspondingly. Please find point-by-point comments below. We hope our revisions will be satisfactory to you and look forward to a positive response.

On behalf of my coauthors,

Eberhard Voit

**Reviewer 1**

1) **Bibliography: The references need to be revised. The current numbering starts with [2], while [1] only appears after [13]. Although [1] is cited in the abstract, it is my understanding that references should not be included in the abstract. If I am mistaken, I apologize, and the numbering is correct as it stands**.

**Response:** Thank you for the suggestion. We have removed the reference from the abstract and renumbered the references in the text.

2) **Symbolic Representation of Template and Anchor Models: Conceptually, using the same symbolic notation for both the Template and Anchor models creates confusion and complicates the understanding of the methodology. While I do not have a definitive solution, I suggest two possible alternatives:

   a) Use the conventional representation for Anchor models, where state variables are represented as typical components, and processes are indicated by flux arrows. Then, adopt a modified graph-theoretical representation (such as those shown in Figures 16 or 17), but ensure that symbols or arrow types are distinct from the flux arrows used in the Anchor models.

   b) Alternatively, employ a simple graphical notation similar to the Systems Biology Graphical Notation (SBGN) for the Template model.**

**In my view, this is the primary methodological issue that needs improvement to enhance the overall clarity of the approach.**

**Response:** That is an interesting point. In response, we have altered the representation of the flux arrows in the template models (Figures 4 and 5) and updated the legend of Figure 5. Fluxes in the template model are now represented as hollow arrows.

3) **Parallel Analysis for Anchor Models: It would be beneficial to conduct parallel analyses for all three Anchor models as well as for the Template model. Performing a Monte Carlo-like analysis for both the first Anchor model and the Template model would allow for a more direct comparison with the other two Anchor models. These analyses could also be used to verify whether the differential sensitivity analysis presented in Supplementary Section S5 is consistent with finite changes.**

**Response:** The sensitivity analysis we present does reflect discrete changes (±10%). In other words, what we did is not a typical sensitivity analysis based on infinitesimally small variations. Instead, we investigate the effects of changes that are actually quite large. A Monte-Carlo analysis would theoretically be feasible but not practical, given the numerous parameters involved in the various models, which would require millions of iterations to capture the full range of overall variability. More importantly, the outcome would be marginal and most likely not justify the required effort, and we would expect the results to amount merely to a footnote-like comment in the *Supplements*.

**4)	Parameter Randomization Methodology: A subsection should be added to the Methods section detailing how the randomization of parameter values is conducted. This would improve transparency and replicability.**

**Response:** We have added a *Methods Section* (2.5) to explain the Monte-Carlo methodology that we used for parameter randomization. Further details are presented in a new table in the *Supplements* S2.3.

**5)	Annotated Equations and Figures: Please address the annotated corrections for the equations and figures/figure captions provided in the attached PDF.**

**>> p.5:**

**Response:** Thank you! We have addressed this comment in the paper.

**Fig.2:**

**Response:** We considered the suggestion of renaming the mevalonate pathway but decided against it, as this is the typical name in the literature.

**Fig.3: Check for genes.**

**Response:** The genes that pertain to the steroidogenesis anchor model are included in Table 1, and the specific genes that are affected are discussed in the text of Section 1.1.5. These genes include: CYP11A1, CYP17A1, HSD3B1, CYP17A1, CYP19A1and HSD17B1. Additionally, we mention that regulation is implemented as power laws within our ODEs. An additional description has been added to the text.

**>> Fig 4:**

**>> Fig.5:**

**Response:** We have updated the representation of the flux arrows in the template models (Figures 4 and 5). Fluxes are now represented as hollow arrows. Also, we have expanded the legend of Figure 5 to explain these issues.

**>> p.18/19**

**Response:** Thank you for your suggestion regarding the inclusion of an additional table in Section 2.12. To maintain conciseness to the manuscript we have opted not to include an additional table as it would largely duplicate information already present.

**>> p.19/20:**

**1. Good point, Do we have DR here?**

**add DRi to equations**

**Response:** Thank you for your comment and sorry for the oversight. The equations have been updated to include DRi in the equations. The complete set of ODEs can be found in section S2.1 of the *Supplements***.**

**2. Explain again how time scales affect a dynamic system**

**Response:** If an ordinary differential equation is multiplied with a positive factor, such as 0.1 or 10, its dynamics runs at a tenth of the speed or ten times as fast as the original. Thus, by choosing a non-zero, non-negative multiplier, the speed of the dynamics can be manipulated. As we only have steady-state information for the calibration of the model, the value of this multiplier is unknown.

We have added an explanation to the text.

**3.**

**>> p.21:**

**Response:** The location has been updated within the text. The complete set of ODEs can be found in section S2.1 of the *Supplements*.

**>> p.23:**

**Response:** A Monte-Carlo analysis would practically not be feasible, given the numerous parameters involved in the various models, which would require millions of iterations to capture the full range of overall variability. We decided that the additional insights from the study would be marginal advances and not warrant the effort.

**>> p.26 and page 28:**

**Response:** We have added a section to discuss the Monte Carlo simulation presented in the paper (see *Methods Section* 2.5).

**>> p.29: A sensitivity analysis, either open loop or monte carlo-like would be nice to have in order to have some measure of the uncertainty in folding results from the anchor to the template models**

**Response:** We appreciate the suggestion to include additional analyses. However, after careful consideration, we have decided not to incorporate this material, as it would introduce additional volume that we believe would distract from the primary focus of the paper, which already is quite long. While this analysis could provide valuable insights, we feel that such an investigation might warrant a separate study in the future.

**>> Fig. 17:**

**Response:** Thank you for the observation. We have updated the figure legend to replace each S with a dashed, blunted arrow.

**Reviewer 2:**

**1. Does the T&A modeling paradigm require a time-scale separation between the dynamics at the template level and those at the anchor level? The example provided seems to use this separation, where the dynamics or response at the anchor level is faster compared to the template level, allowing it to be approximated as being at a steady state.**

**Response:** That is a very interesting comment. Thank you! In our case, the expectation is that A will usually have a faster dynamics than T. Whether this relationship is mathematically necessary though is a research question with an unknown answer; it should be investigated in the future. We have added a sentence to the Discussion.

2. When calibrating dose responses to data, it would be helpful to include the data on the associated plots. This would allow readers to assess the quality of the fitting more effectively.

**Response:** The data for calibration are scarce, but we have now included a set in the Methods Section.
